# On-surface synthesis and characterization of nitrogen-substituted undecacenes

**DOI:** 10.1038/s41467-022-27961-1

**Published:** 2022-01-26

**Authors:** Kristjan Eimre, José I. Urgel, Hironobu Hayashi, Marco Di Giovannantonio, Pascal Ruffieux, Shizuka Sato, Satoru Otomo, Yee Seng Chan, Naoki Aratani, Daniele Passerone, Oliver Gröning, Hiroko Yamada, Roman Fasel, Carlo A. Pignedoli

**Affiliations:** 1 Empa, Swiss Federal Laboratories for Materials Science and Technology, Überlandstrasse 129, 8600 Dübendorf, Switzerland; 2 IMDEA Nanoscience, C/ Faraday 9, Campus de Cantoblanco, 28049 Madrid, Spain; 3 Division of Materials Science, Nara Institute of Science and Technology (NAIST), 8916-5 Takayama-cho, Ikoma, 630-0192 Japan; 4 Istituto di Struttura della Materia-CNR (ISM-CNR), via Fosso del Cavaliere 100, 00133 Roma, Italy; 5 Department of Chemistry, Biochemistry and Pharmaceutical Sciences, University of Bern, Freiestrasse 3, 3012 Bern, Switzerland

**Keywords:** Scanning probe microscopy, Electronic properties and materials

## Abstract

Heteroatom substitution in acenes allows tailoring of their remarkable electronic properties, expected to include spin-polarization and magnetism for larger members of the acene family. Here, we present a strategy for the on-surface synthesis of three undecacene analogs substituted with four nitrogen atoms on an Au(111) substrate, by employing specifically designed diethano-bridged precursors. A similarly designed precursor is used to synthesize the pristine undecacene molecule. By comparing experimental features of scanning probe microscopy with ab initio simulations, we demonstrate that the ground state of the synthesized tetraazaundecacene has considerable open-shell character on Au(111). Additionally, we demonstrate that the electronegative nitrogen atoms induce a considerable shift in energy level alignment compared to the pristine undecacene, and that the introduction of hydro-aza groups causes local anti-aromaticity in the synthesized compounds. Our work provides access to the precise fabrication of nitrogen-substituted acenes and their analogs, potential building-blocks of organic electronics and spintronics, and a rich playground to explore π-electron correlation.

## Abstract (web summary)

Heteroatom substitution in larger acenes represents a fundamental step towards precise engineering of the remarkable electronic properties of the acene family. Here, the authors present an on-surface synthesis strategy and detailed characterization for three undecacene analogs substituted with four nitrogen atoms.

## Introduction

Acenes, polycyclic aromatic hydrocarbons (PAHs) consisting of linearly fused benzene rings, have attracted a great deal of interest for their remarkable electronic properties. As the length of the acene is increased, the energy gap between its highest occupied molecular orbital (HOMO) and lowest unoccupied molecular orbital (LUMO) reduces rapidly, with already anthracene and tetracene (three and four linearly fused benzene rings, respectively) possessing semiconducting properties^1–3^. The small electronic gap together with high-electron mobility renders acenes attractive materials in organic electronics^4,5^, for applications, such as organic field-effect transistors (OFETs)^6^, organic light-emitting diodes (OLEDs), organic photovoltaic (OPV) devices^7^, and spintronic devices^8^. Acenes have been shown to exhibit singlet fission^9^, which could considerably increase OPV photoconversion efficiencies. In addition to the many potential applications, acenes, and especially higher acenes, provide a rich playground to explore electronic correlation and magnetism effects due to their unique π-bond topology. There is still no clear scientific consensus on the nature of the ground state of higher acenes (from hexacene onwards). A multitude of computational studies has found the ground state to be open-shell polyradical^10–12^, while a recent quantum Monte Carlo study predicted a closed-shell ground state^13^.

Heteroatom substitution is an effective method to tune the electronic, magnetic, and physico-chemical properties of graphene-based materials^14,15^. When applied to acenes, replacing CH groups with heteroatoms (B, P, O, S, N) produces oligoheteroacenes, whose properties depend on the type, number, and position of the heteroatoms^16,17^. For example, boron and nitrogen substitution has been shown to offer precise control over the radical character and chemical stability of acenes^18^. If all the heteroatoms are nitrogen, the compounds are called azaacenes (or N-heteroacenes). Azaacenes inherit the remarkable properties of acenes due to the nitrogen atom leaving the topology of the π-system equivalent with respect to the substituted CH group. The electronegative nitrogen atoms allow for finely tunable frontier molecular orbital energy alignment. Azaacenes have been shown to exhibit n-type semiconducting behavior and have been used in thin-film transistors, OLEDs, and in OPV devices^19,20^.

Synthesis and characterization of larger acenes (from pentacene onwards) has been a challenge for solution chemistry due to their low solubility and high reactivity^21,22^. Nevertheless, successful solution synthesis protocols are available for higher acenes by functionalizing them with stabilizing and protecting groups^23–25^. Furthermore, the Strating–Zwanenburg reaction^26^ has been successfully used for the synthesis of hexacene to undecacene^27–30^ (except for decacene), although such compounds need to be stabilized in a matrix in order to keep them from decomposing after a few hours. In addition, solution chemistry has also been applied to successfully synthesize azapentacenes^31^, azahexacenes^32^, and azaheptacenes^33,34^ by utilizing various bulky protecting groups^19^.

On-surface chemistry has emerged as an alternative, powerful method that allows synthesizing new organic materials by one- or two-dimensional spatial confinement on single-crystal substrates under ultrahigh vacuum (UHV) conditions^35^. The molecular structure and electronic properties of the targeted nanomaterials can be identified via advanced scanning probe techniques, such as scanning tunneling microscopy/spectroscopy (STM/STS) and non-contact atomic force microscopy (nc-AFM). In the last decade, the research field of on-surface synthesis has experienced a considerable expansion and provided an appealing playground for the design and investigation of organic materials that have remained elusive due to synthetic limitations in solution chemistry. For instance, the on-surface synthesis of graphene nanoribbons (GNRs)^36^, the discovery of novel chemical reactions^35,37^, or the synthesis of carbon-based nanographenes with intriguing electronic properties^38–42^ have contributed significantly toward the development of the on-surface chemistry toolbox. Particular attention has recently been paid to the synthesis and characterization of higher acenes on surfaces^43–49^, with the largest synthesized members being decacene^50^, undecacene^46^, and dodecacene^51^. Herein, the use of α-diketone-, epoxy- and hydrogen-protected acene precursors, which provide them with the required solubility and stability, followed by thermal or light-induced activation once deposited on the metal substrate, have typically been the synthesis strategies employed for the successful formation of pristine higher acenes. Interestingly, the experimental frontier orbital gap for the series of acenes of increasing length is monotonically decreasing to around 1.0 eV for undecacene and unexpectedly increasing to around 1.4 eV for dodecacene, which the authors attribute either to a charge transfer or an electronic correlation effect^51^. Despite these successful cases exist for the synthesis of higher acenes, and despite the existence of on-surface approaches to synthesize heteroatom-substituted graphene-derived systems^14,15,52–58^, the investigation of higher heteroacenes is so far unexplored.

**Fig. 1:**
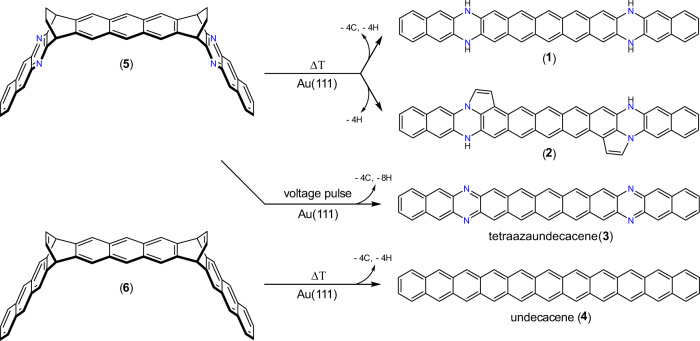
On-surface synthetic strategy. Schematic representation of the synthesis of N,N′,N″,N‴-tetrahydro-6,12,19,25-tetraazaundecacene **1**, its analog with two edge-fused five-membered rings **2**, 6,12,19,25-tetraazaundecacene **3**, and the pristine undecacene **4** from the corresponding specifically designed precursors **5** and **6**.

Here, we introduce the on-surface formation of hydrogenated tetraazaundecacene (N,N′,N″,N‴-tetrahydro-6,12,19,25-tetraazaundecacene) **1**, its analog with two edge-fused five-membered rings **2**, and the 6,12,19,25-tetraazaundecacene **3** via thermal annealing and STM tip manipulation of a novel-design precursor molecule on a coinage metal surface under UHV conditions (Fig. 1). We also demonstrate that a similarly designed precursor can be used to synthesize the pristine undecacene **4**. For this purpose, diethano- and dietheno-bridged precursors (**5** and **6**, respectively), which are thermally stable upon sublimation, were synthesized in solution (see Supplementary Methods: Precursor Synthesis and Characterization). In addition to the detailed chemical and structural properties unveiled via STM and nc-AFM measurements, we report on STS experiments to characterize the electronic structure of the synthesized compounds. In combination with theoretical calculations, this allows us to determine whether the obtained compounds present an open or closed-shell electronic ground state on the substrate. Additionally, ab initio computational methods are used to characterize and compare the electronic properties, such as the radical character, ionization potential, and aromaticity, of the synthesized compounds in detail.

## Results and discussion

### Deposition of precursor 5 on Au(111) and thermally induced transformations

**Fig. 2:**
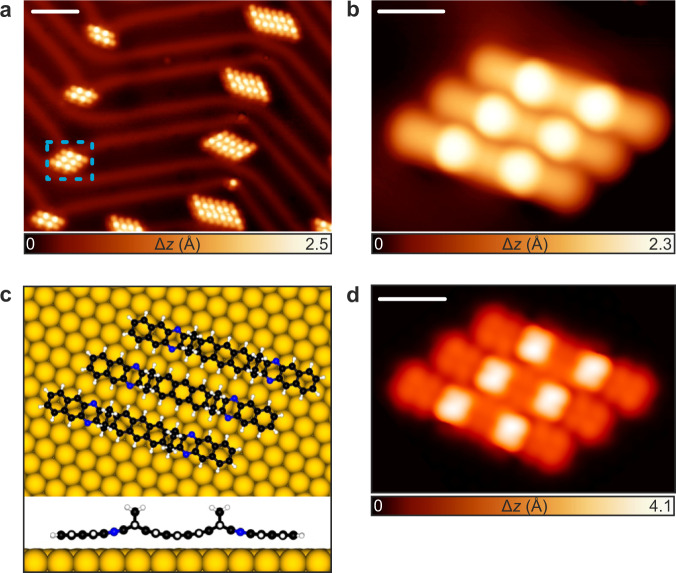
Identification of the precursor **5** after deposition on Au(111). **a** Large-scale STM topography image of the surface after room temperature deposition of **5**. *V*_b_ = −1 V, *I*_t_ = 100 pA, scale bar = 5 nm. Δ*z* denotes the relative height w.r.t. clean Au(111) surface. **b** Magnified view of one of the self-assembled structures shown in panel **a** containing three molecules. *V*_b_ = −0.5 V, *I*_t_ = 50 pA, scale bar = 1 nm. **c** Top and side views of the DFT equilibrium geometry of a cluster of three molecules **5** on Au(111). **d** DFT-simulated constant-current STM image of the three self-assembled molecules shown in panel **a**, *V*_b_ = −0.5 V, scale bar: 1 nm.

In order to study the on-surface reactions described in Fig. 1, a submonolayer coverage of **5** was sublimed onto an Au(111) surface held at room temperature. Figure 2a shows a representative large-scale STM image revealing the presence of several rod-like self-assembled molecules mainly located at the face-centered cubic (fcc) regions of the Au(111) surface. High-resolution STM images (Fig. 2b) allow us to discern the intramolecular features of **5**, where two bright protrusions per molecule of the similar apparent height of 2.3 Å (measured at a sample bias of −0.5 V) are observed. According to structural models, the self-assembled molecules are stabilized by N···H interactions between adjacent molecules^59^ with an experimental projected average distance of 3.2 ± 0.3 Å. Such experimental features, observed in Fig. 2b, are in good agreement with the DFT-optimized geometry and the corresponding STM simulation of **5** on the Au(111) surface (Fig. 2c, d), which indicate that **5** adopts a *syn* conformation with the two out-of-plane ethano bridges pointing upwards and an adsorption height of 2.8 Å with respect to the underlying surface.

**Fig. 3:**
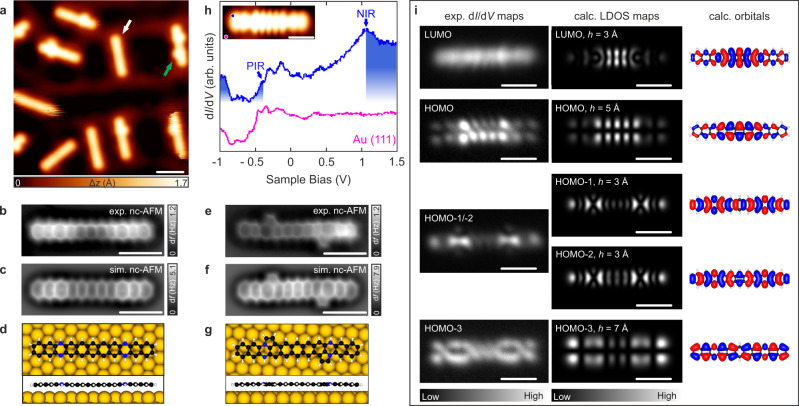
On-surface synthesis of the hydrogenated tetraazaundecacene **1** and the analog with edge-fused five-membered rings **2** by annealing the sample at 280 °C. **a** Overview STM topography image of the surface after annealing, showing multiple linear uniform species (one highlighted by the white arrow) and species with laterally extending features near one or both (highlighted by green arrow) of the nitrogen sites. *V*_b_ = −0.2 V, *I*_t_ = 70 pA, scale bar: 2 nm. Δ*z* denotes the relative height w.r.t. clean Au(111) surface. **b**, **e** Constant-height frequency-shift nc-AFM images of the two highlighted cases acquired with a CO-functionalized tip (*z* offset −25 pm below STM set point: 5 mV, 30 pA), scale bar: 1 nm. **c**, **f** Simulated nc-AFM images for **1** and **2** based on the DFT equilibrium geometry. **d**, **g** DFT equilibrium adsorption geometry of **1** and **2** on Au(111). **h** d*I*/d*V* spectrum acquired on **1** at the position indicated by the blue dot in the inset (inset shows an STM image with *V*_b_ = −0.4 V, *I*_t_ = 40 pA, scale bar: 1 nm). PIR and NIR stand for positive and negative ion resonance, respectively. Reference d*I*/d*V* spectrum taken on the bare Au(111) surface is depicted in pink. **i** Constant-height differential conductance (d*I*/d*V*) maps acquired at bias voltages 1.00, −0.45, −0.80, −1.20 V (first column; from top to bottom) together with the corresponding DFT-calculated LDOS maps (second column) and corresponding molecular orbital isosurfaces at isovalues ±0.01 a.u. (atomic units; third column). Scale bars: 1 nm. The experimental maps are assigned to molecular orbitals based on the matching with calculated maps.

Following the proven strategy for the formation of higher acenes on metallic surfaces^43,44,48–51^, we have annealed the substrate at 280 °C, which induces the planarization of most of the molecular species **5**, as judged from the absence of the bright protrusions of higher apparent height (Fig. 3a). This observation is attributed to the cleavage of the ethano protecting groups via retro-Diels–Alder reaction^60–62^, observed for ~90% of the molecules, which coexist with species where one or two bright protrusion per molecule are still present. Notably, a statistical analysis of the sample (out of >200 molecules) shows that 25% of the new species appear as smooth rod-like structures, while the remaining molecules present one (33%) or two (42%) lateral protrusions. To characterize the chemical structure of the observed species, nc-AFM measurements using a CO-functionalized tip were performed^63^. The constant-height frequency-shift image together with the simulated nc-AFM image depicted in Fig. 3b, c confirms the successful formation of the hydrogenated tetraazaundecacene species **1**. The corresponding DFT equilibrium geometry, shown in Fig. 3d, indicates physisorption with an adsorption height of 3.1 Å. Furthermore, Fig. 3e, f allow us to elucidate the chemical structure of the lateral protrusions (highlighted with a green arrow in Fig. 3a), which are attributed to five-membered rings (see Supplementary Fig. 1 for a zoom-in nc-AFM image of **2**, where the newly formed five-membered rings are clearly discerned). They hypothetically derive from the bond cleavage at the bridgehead position of bicyclo[2.2.2]octadieno unit, with the terminal part of the resulting partial structure re-bonding to the neighboring nitrogen atom. These steps lead to the removal of two protons of the ethano group by dehydrogenative aromatization. Again, for product **2**, the DFT equilibrium geometry shows physisorption with an adsorption height of 3.1 Å (Fig. 3g).

The observed hydrogenation of the edge nitrogen atom(s) might appear surprising at first sight, but it must be noted that the deprotection reaction takes place on a surface exhibiting a significant density of mobile hydrogen adatoms. Furthermore, under UHV conditions, hydrogen is the predominant compound in the rest gas of the vacuum chamber, and hydrogen atoms may also be produced by surface-promoted dissociation after cleavage of the ethano protecting groups. Similar passivation of reactive molecules or GNRs on surfaces has been reported before^40,43,64–66^.

Next, we have probed the electronic structure of the obtained species **1** via scanning tunneling spectroscopy (STS) measurements. Figure 3h shows the voltage-dependent differential conductance spectrum (d*I*/d*V* vs *V*) with peaks in the density of states at −0.45 V and +1.10 V, which we assigned to the positive and negative ion resonances (PIR and NIR, respectively). The spectrum has spurious peaks between −0.35 and −0.10 V, but these do not show features in the d*I*/d*V* maps of the molecule and therefore are attributed to tip states (Supplementary Fig. 2). Using a CO-functionalized tip in constant-height mode, we mapped the d*I*/d*V* signal at the energetic positions of the NIR, PIR, and two subsequent ion resonances detected at −0.80 and −1.20 V, shown in Fig. 3i, left column, respectively from top to bottom. These d*I*/d*V* maps show an excellent agreement with the DFT-calculated local density of states (LDOS) maps of the frontier orbitals (Fig. 3i, middle and right column) and allow to assign the NIR to LUMO and PIR to HOMO of **1**. The resonance at −0.80 V can be considered to originate from hybridization of HOMO−1 and HOMO−2, and the resonance at −1.20 V can be assigned to HOMO−3. The LDOS maps for this system are remarkably sensitive to height variation and therefore the simulation height matching best to the experiment was chosen. For the height variation and comparison to experimental constant-current d*I*/d*V* maps, see Supplementary Fig. 3. The assignment of the ion resonances to the molecular orbitals confirms the chemical structure of **1**, and more specifically, the possibility of these species to be assigned to tetraazaundecacene **3** is ruled out at this stage by the presence of the HOMO−1/HOMO−2 resonance at −0.80 V (as this resonance is absent for **3**, see Supplementary Fig. 4). The assignment of PIR and NIR to HOMO and LUMO allows us to infer an experimental HOMO-LUMO gap of 1.55 eV of **1** on Au(111), which is in good agreement with the theoretically predicted gap of 1.77 eV, calculated using many-body perturbation theory within the GW approximation, including screening effects from the underlying gold substrate (for details, see the Calculation methods section and Supplementary Methods: Computational Details).

### Synthesis of tetraazaundecacene by STM tip-induced deprotection

**Fig. 4:**
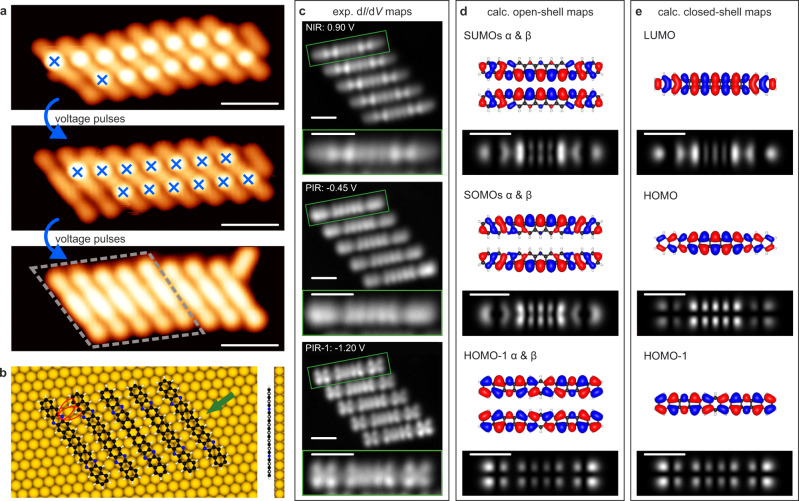
On-surface synthesis of tetraazaundecacene **3** via STM tip-induced cleavage of the ethano protecting groups of **5** and electronic characterization. **a** Successive STM topography images illustrating the cleavage process. Top image shows a cluster of **5** (leftmost molecule has already one bridge cleaved) with blue crosses indicating the locations where the voltage pulse is applied. Middle image shows the intermediate state after two voltage pulses, and bottom image shows the final result after applying a voltage pulse to all the molecules of the cluster. Scale bars: 5 nm. **b** DFT-calculated equilibrium geometry of a cluster of five molecules of **3**. Red ovals indicate hydrogen bonds. The side view is shown for a single molecule in the direction indicated by the green arrow. **c** Experimental constant-height d*I*/d*V* maps of the frontier states for a cluster of five tetraazaundecacene molecules. PIR−1, PIR, and NIR stand for the second positive, the positive, and the negative ion resonances, respectively. Each map includes a zoom-in of a single molecule indicated by the green rectangle. Scale bars: 1 nm. **d**, **e** DFT-calculated molecular orbitals (α, β denote the two spin channels) and LDOS mappings at height 5 Å for the broken-symmetry open-shell singlet and the closed-shell state, respectively. Scale bars: 1 nm.

Hydrogen passivation of the edge nitrogen atoms can be prevented if the annealing step is avoided and alternatively a tip-induced cleavage of the ethano bridges is applied. Figure 4a depicts the sequential tip-induced cleavage of the protecting groups from eight self-assembled molecules **1**, which is achieved by applying a voltage ramp (−1.0 to −3.0 V) to the STM tip with feedback loop off, as previously reported for the cleaving of α-diketone moieties equipped in heptacene and nonacene precursors^43,48^. Thus, tip-induced release of the protecting group can be employed to produce the targeted tetraazaundecacene species **3** (see Supplementary Fig. 5 for a comparison between experimental and simulated nc-AFM images between species **1** and **3**). The resulting tetraazaundecacene molecules **3** remain self-assembled, stabilized by N···H interactions with identical average intermolecular distances as those described for the precursor **5** in Fig. 2c. The existence of N···H hydrogen bonds for **3**, that enables the self-assembly, is also supported by analysis of the electrostatic potential (Supplementary Fig. 6), while the hydrogenated species **1** and **2**, and the pristine undecacene **4** lack this interaction.

Figure 4b shows the DFT-optimized geometry of the five self-assembled tetraazaundecacene molecules **3** highlighted by a gray dashed rhombus in Fig. 4a, with the N···H interactions indicated by red ellipses. The DFT calculated adsorption height of **3** within this self-assembled cluster structure is 3.1 Å. Constant-height d*I*/d*V* maps acquired on a similar cluster of five self-assembled **3** (Fig. 4c) reveal the NIR at 0.90 V, PIR at −0.45 V and an additional resonance at −1.20 V. The experimental ion resonance maps are in good agreement with the DFT calculated LDOS maps (Fig. 4d, e), further confirming the successful synthesis of **3**, and allow to assign the NIR and PIR to frontier orbitals and the resonance at −1.20 V to HOMO−1 (see Supplementary Fig. 4 for additional maps and calculated height dependence). The only qualitative difference between the LDOS maps calculated for the broken-symmetry open-shell singlet solution (Fig. 4d) and for the closed-shell solution (Fig. 4e) is the existence of the nodal plane along the center of the molecule in the HOMO map compared to the SOMO map. The corresponding experimental PIR map does not exhibit a reduction in the signal along the center, indicating that the experimental ground state has considerable open-shell character. The experimental frontier orbital gap, derived from the difference between PIR and NIR, is 1.35 eV. A GW calculation with image charge corrections for the open-shell ground state gives a gap value of 0.97 eV, which matches better with the experiment than the closed-shell gap of 0.74 eV, thus giving further evidence for a significant open-shell character in the ground state of tetraazaundecacene **3** on Au(111).

### Synthesis of pristine undecacene

In addition to the diethano-bridged precursor **5** affording the tetraazaundecacenes discussed above, we have expanded our on-surface synthesis protocol toward the formation of pristine undecacene **4**, taking advance of precursors containing dietheno protecting groups (compound **6**). Annealing of **6** on Au(111) to 220 °C results in a variety of products, including the targeted undecacene **4** (Supplementary Fig. 7). Experimental d*I*/d*V* maps acquired on **4** reveal PIR and NIR at −0.25 and 0.70 V, respectively, which can be assigned to frontier orbitals by comparing to DFT-simulated LDOS maps (Supplementary Fig. 7d–f). The experimental maps of the frontier orbitals show features of both, closed and open-shell calculated maps, which is again an indication of considerable open-shell character, similar to the case of tetraazaundecaene **3**. This is in agreement with the demonstration of radical contributions in the ground state of higher acenes in literature^51,67^, and computational studies predicting that undecacene tilts more to the open-shell side of the diradical continuum^11,12^. The experimental frontier gap for undecacene (difference between PIR and NIR) is 0.95 eV, in line with values reported in literature^46^. This experimental gap matches very well with the GW (plus image charge screening correction) calculation resulting in 1.03 eV, while the equivalent calculation for the closed-shell solution gives a gap of 0.70 eV, thus giving further evidence for the open-shell character of undecacene adsorbed on Au(111).

### Theoretical characterization of the synthesized compounds

To compare the electronic structure of the synthesized compounds in further detail, we performed gas-phase DFT geometry optimization (Supplementary Fig. 8) and calculations of the ground spin state, ionization potential, and radical character (Supplementary Table 1). As expected, due to the equivalent topology of the π-system and electron filling in the undecacene **4** and the tetraazaundecacene **3**, their electronic properties are practically equivalent except for the ionization potential and thus the energy alignment of the orbitals. For both systems, DFT with the B3LYP functional predicts an open-shell singlet ground state with the triplet state being 0.2 eV and the closed-shell state 0.5 eV higher in energy. Additionally, the ground state frontier gaps are ~1.7 eV, and unrestricted Hartree–Fock with spin-projection predicts a biradical character^68^ of 0.95 and a total of 3.7 unpaired electrons in both cases. The equivalence of the π-systems is also demonstrated in Supplementary Fig. 9, which shows that a wide range of frontier orbitals for the closed-shell solution are equivalent for **4** and **3**. To quantify the orbital energy alignment, we performed ΔSCF calculations that predict the ionization potentials of undecacene **4** and tetraazaundecacene **3** to be 5.63 and 6.12 eV, respectively, showing that the electronegative nitrogen atoms of tetraazaundecacene cause the electronic orbitals to be aligned 0.5 eV lower with respect to the vacuum level than those of pristine undecacene.

The hydrogenated species **1** and **2** are both predicted to be closed-shell by DFT (Supplementary Table 1) with similar electronic properties: triplet state energy is respectively +0.94 and +1.08 eV higher than the closed-shell state, and the ground state frontier gaps are 2.2 and 2.3 eV, respectively. Also, the ionization potential predicted by ΔSCF is similar for both: 5.47 and 5.42 eV for **1** and **2**, respectively. Interestingly, the geometry of **1** and **2** is fully planar (Supplementary Fig. 8), which indicates that the nitrogen atoms do not break the π-conjugation and each of them provides 2 electrons to the π-system of the molecule. In the case of **1**, this is also confirmed by comparing the DFT-predicted electronic orbitals to those of **4** and **3** (Supplementary Fig. 9). The orbitals of **1** exhibit the same nodal structure as those of **4** and **3**, with 2 extra orbitals occupied by the 4 extra electrons provided by the NH groups. The lower-lying orbitals show the same energy-ordering for the three systems, but the extra occupations in **1** cause the orbitals from HOMO−2 (number 151) onwards to reorder in energy with respect to **4** and **3**. Interestingly, the reordering happens such that the same orbital stays HOMO in all three cases.

**Fig. 5:**
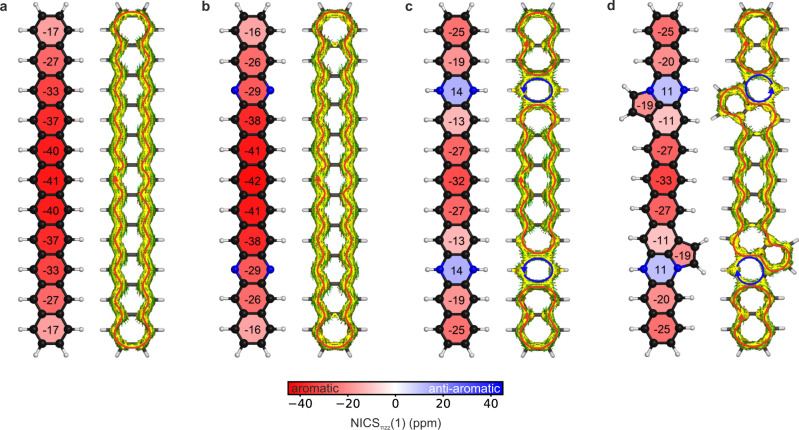
Computational characterization of the aromaticity of the synthesized undecacene and tetraazaundecacenes. **a** NICS_πzz_(1) patterns (each cycle is represented by the NICS_πzz_(1) evaluated at the center) and π-ACID plot (magnetic field is applied perpendicular to the plane of the system, pointing toward the reader; red arrows indicate aromatic diatropic and blue arrows anti-aromatic paratropic ring current) for the closed-shell solution of the undecacene **4**. **b** NICS_πzz_(1) pattern and π-ACID plot for closed-shell solution of the tetraazaundecacene **3**. **c** NICS_πzz_(1) pattern and π-ACID plot for the hydrogenated tetraazaundecacene **1**. **d** NICS_πzz_(1) pattern and π-ACID plot for the hydrogenated tetraazaundecacene with edge-fused five-membered rings **2**. All π-ACID plots are showing the isosurface at isovalue 0.05 a.u.

In order to further characterize the π-system and the impact of the nitrogen substitution, we calculated the aromaticity pattern of the systems using the nucleus independent chemical shift (NICS) method^69^. More specifically, we used the NICS_πzz_(1) variant, which considers the out-of-plane component of the magnetic shielding tensor due to the π orbitals at 1 Å above the center of each ring^70^. To characterize electron delocalization in the π-system and the aromatic ring currents, we performed anisotropy of the induced current density (ACID) calculations on the π orbitals of the systems (π-ACID)^71^. The results are shown in Fig. 5. The aromaticity patterns for the undecacene **4** and the tetraazaundecacene **3**, calculated for the closed-shell solution, show a similar behavior: the central region of the acene is strongly aromatic and a strong diatropic current flows around the whole periphery of the molecule. The open-shell solution for both cases, however, exhibits a positive NICS_πzz_(1) value in the central region (Supplementary Fig. 10). This behavior has been reported in literature^72,73^, although for shorter acenes and therefore showing a less significant effect. The positive NICS_πzz_(1) value is an artificial effect introduced by the contamination of the open-shell singlet ground state with the triplet state (Supplementary Table 1), which exhibits Baird’s anti-aromaticity^74^. The π-ACID analysis for the open-shell singlet shows a diatropic current, although weaker than in the closed-shell solution, but still indicating aromaticity in the system. The hydrogenated tetraazaundecacene **1** and its analog with edge-fused five-membered rings **2** show almost equivalent NICS_πzz_(1) pattern on the acene backbone (Fig. 5c, d, Supplementary Fig. 10 and 11), in line with the similarity of the electronic properties previously discussed, showing that the five-membered rings, which are aromatic themselves, have negligible effect on the aromatic pattern of the remaining system. Interestingly, for both systems, the cycles containing the nitrogen show sharp antiaromaticity^75^. This further confirms the picture that the nitrogen atoms provide 2 electrons each to the π-system, making the nitrogen-containing cycles have 8 π electrons, which are formally anti-aromatic according to Hückel’s rule. The π-ACID analysis confirms this nicely: the rings containing the two nitrogen atoms show a clear paratropic contribution in the delocalized π-system, while the remaining parts show a diatropic current around their peripheries.

In conclusion, the on-surface synthesis of tetraazaundecacene, hydrogenated tetraazaundecacene and its analog with edge-fused five-membered rings reported in this article represents a fundamental step toward the precise engineering and tailoring of the unique electronic properties of the (hetero)acene family. Nc-AFM, STM, and STS differential conductance map unambiguously demonstrate the successful formation of the three targeted structures on an Au(111) surface. By comparing the experimental differential conductance maps and the frontier orbital gap with density functional theory LDOS maps and GW-calculated gap, we are able to assign an experimental ground state with considerable open-shell character for the synthesized tetraazaundecacene on Au(111), while the other two compounds are found to be closed-shell. Calculations based on DFT demonstrate that the tetraazaundecacene retains the remarkable electronic properties of the pristine undecacene with the exception of a considerable downshift in the orbital energies introduced by the electronegative nitrogen atoms. Additionally, characterization of the aromaticity shows that the hydrogenated tetraazaundecacene and its analog with edge-fused five-membered rings exhibit unique sharp anti-aromaticity in the rings containing the nitrogen atoms. Finally, the presented procedure offers a new perspective to investigate the complex electronic correlation effects in acenes by chemical substitution and opens up a new path toward precisely tailored building blocks of organic electronics and spintronics.

## Methods

### Sample preparation and STM/nc-AFM measurements

Experiments were performed under UHV conditions (base pressure below 4 × 10^−10^ mbar) with a commercial low-temperature STM/AFM from Scienta Omicron.

The Au(111) substrate was prepared by repeated cycles of Ar^+^ sputtering (*E* = 1 keV) and subsequent annealing to ~450 °C for 10 min. All STM images shown were taken in constant current mode, unless otherwise noted, with electrochemically etched tungsten tips at a sample temperature of 4.8 K. Scanning parameters are specified in each figure caption. Molecular precursors (**5** and **6**) were thermally deposited onto a clean Au(111) surface held at room temperature with a typical deposition rate of 0.5 Å min^−1^ and 0.8 Å min^−1^ (sublimation temperatures ~400 and 410 °C, respectively).

Nc-AFM measurements were performed with a tungsten tip attached to a Qplus tuning fork sensor^76^. The tip was a posteriori functionalized by the controlled adsorption of a single CO molecule at the tip apex from the previously CO-dosed surface. The functionalized tip enables the imaging of the intramolecular structure of organic molecules^63^. The sensor was driven at its resonance frequency (27,220 Hz) with a constant amplitude of ~70 pm. The shift in the resonance frequency of the tuning fork (with the attached CO-functionalized tip) was recorded in constant-height mode (Omicron Matrix electronics and HF2Li PLL by Zurich Instruments). The STM and nc-AFM images were analyzed using WSxM.

### Calculation methods

The CP2K software package^77^ was used to calculate the equilibrium adsorption geometries and to perform the many-body perturbation theory calculations in the GW approximation^78^. We used PBE functional with DFT-D3 dispersion corrections. The GW quasiparticle energy levels were renormalized according to the image charge model by Neaton et al. ^79^ to account for the screening by the metal substrate. The AiiDAlab platform^80^ was used to perform the calculations for the molecules adsorbed on Au(111).

The gas-phase electronic structure, LDOS maps, and aromaticity calculations were performed with the Gaussian software package^81^. The B3LYP functional was used in the spin-restricted and unrestricted formalism (respectively for the closed and open-shell states). The 6-311G** basis set was used for geometry optimizations, while single point and aromatic properties were calculated using the 6-311+G** basis set. The NICS_πzz_(1)^69,70^ was calculated with the GIAO-B3LYP method by applying the canonical molecular orbital (CMO) natural chemical shielding analysis (NCS) within the NBO program suite^82^. The ACID^71^ calculations were performed with the CSGT method.

Full details about the computational methodology can be found in the Supplementary Methods: Computational Details.

## Supplementary information

Supplementary Information

## Data Availability

The data that supports the results of this study are available on the Materials Cloud platform (10.24435/materialscloud:mc-kj).
