# Supplementary material for: On-surface synthesis and characterization of nitrogen-substituted undecacenes
Source: Nat Commun. 2022 Jan 26;13:511. doi: 10.1038/s41467-022-27961-1 (PMC8791976; doi:10.1038/s41467-022-27961-1)
Supplement: Supplementary file 1 — Supplementary Information [file 41467_2022_27961_MOESM1_ESM.pdf]

Supplementary information

On-Surface Synthesis and Characterization of Nitrogen-Substituted Undecacenes

Kristjan Eimre[1,Δ,*], José I. Urgel[1,2,Δ,*], Hironobu Hayashi[3,Δ], Marco Di Giovannantonio[1,4], Pascal Ruffieux[1], Shizuka Sato[3], Satoru Otomo[3], Yee Seng Chan[3], Naoki Aratani[3], Daniele Passerone[1], Oliver Gröning[1], Hiroko Yamada[3,*], Roman Fasel[1,5,*], Carlo A. Pignedoli[1,*]

[1]Empa, Swiss Federal Laboratories for Materials Science and Technology, Überlandstrasse 129, 8600 Dübendorf, Switzerland

[2]IMDEA Nanoscience, C/ Faraday 9, Campus de Cantoblanco, 28049 Madrid, Spain

[3]Division of Materials Science, Nara Institute of Science and Technology (NAIST), 8916-5 Takayama-cho, Ikoma, 630-0192, Japan

[4]Istituto di Struttura della Materia-CNR (ISM-CNR), via Fosso del Cavaliere 100, 00133 Roma, Italy.

[5]Department of Chemistry, Biochemistry and Pharmaceutical Sciences, University of Bern, Freiestrasse 3, 3012 Bern, Switzerland

[Δ]Contributed equally.

*Corresponding authors: Kristjan Eimre kristjan.eimre@empa.ch, José I. Urgel jose-ignacio.urgel@imdea.org, Hiroko Yamada hyamada@ms.naist.jp, Roman Fasel roman.fasel@empa.ch, Carlo A. Pignedoli carlo.pignedoli@empa.ch

Table of Contents

- Supplementary Figures 1-7 describing supporting nc-AFM, STM/STS experimental data and accompanying calculations.
- Supplementary Figures 8-11 and Supplementary Table 1 describing supporting computational characterization.
- Supplementary Methods: Computational Details
- Supplementary Methods: Precursor Synthesis and Characterization
    1. General methods and materials
    2. Synthetic procedures (Supplementary Figures 12, 13)
    3. X-ray single crystal structure measurements (Supplementary Figures 14, 15)
    4. Nuclear magnetic resonance (NMR) spectra (Supplementary Figures 16-37)
    5. Mass spectra and high-resolution mass spectra (Supplementary Figures 38-57)
- Supplementary References

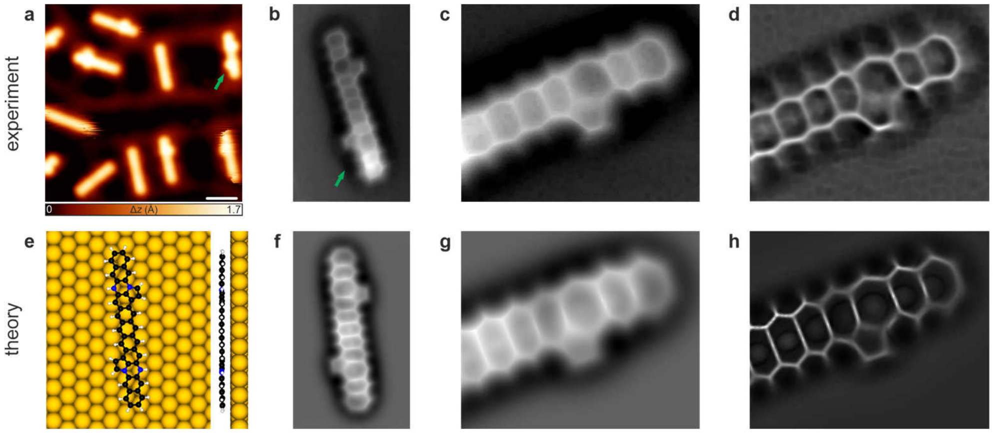

Supplementary Figure 1 Comparison of experimental and simulated constant-height frequency-shift nc-AFM images for the hydrogenated tetraazaundecacene species with edge-fused five-membered rings **2**. **a** Overview STM topography image of the surface after annealing **5** at 280 °C, showing a case with laterally extending features near one or both (highlighted by green arrow) of the nitrogen sites. V_b = -0.2 V, I_t = 70 pA, scale bar = 2 nm. **b** nc-AFM image of the highlighted case, showing the five-membered ring, acquired with a CO-functionalized tip. Open feedback parameters: V_b = 5 mV. **c** Zoomed-in nc-AFM image of the five-membered ring. **d** Laplace filtered nc-AFM image shown in panel (**c**). **e** DFT equilibrium adsorption geometry of **2** on Au(111). **f,g** Simulated nc-AFM images for **2** based on the DFT equilibrium geometry. **h** Laplace filtered simulated nc-AFM image shown in panel (**g**).

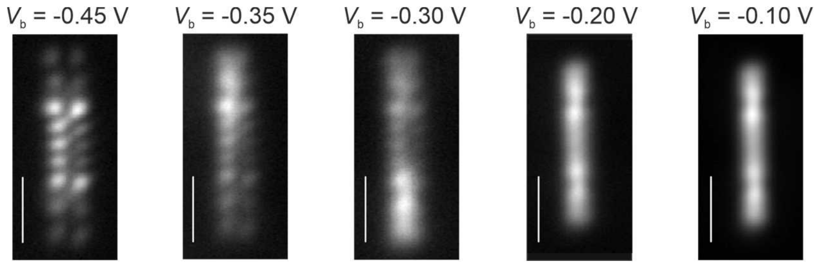

Supplementary Figure 2 Constant-height d*I*/d*V* maps of **1** recorded at different bias voltages V_b. No features are observed for maps recorded at -0.1 V and -0.2 V. Faint features shown in the d*I*/d*V* maps recorded at -0.3 V and -0.35 V correspond to the energy broadening of the state recorded at -0.45 V. All maps were acquired with an I_t = 300 pA. Scale bars: 1 nm

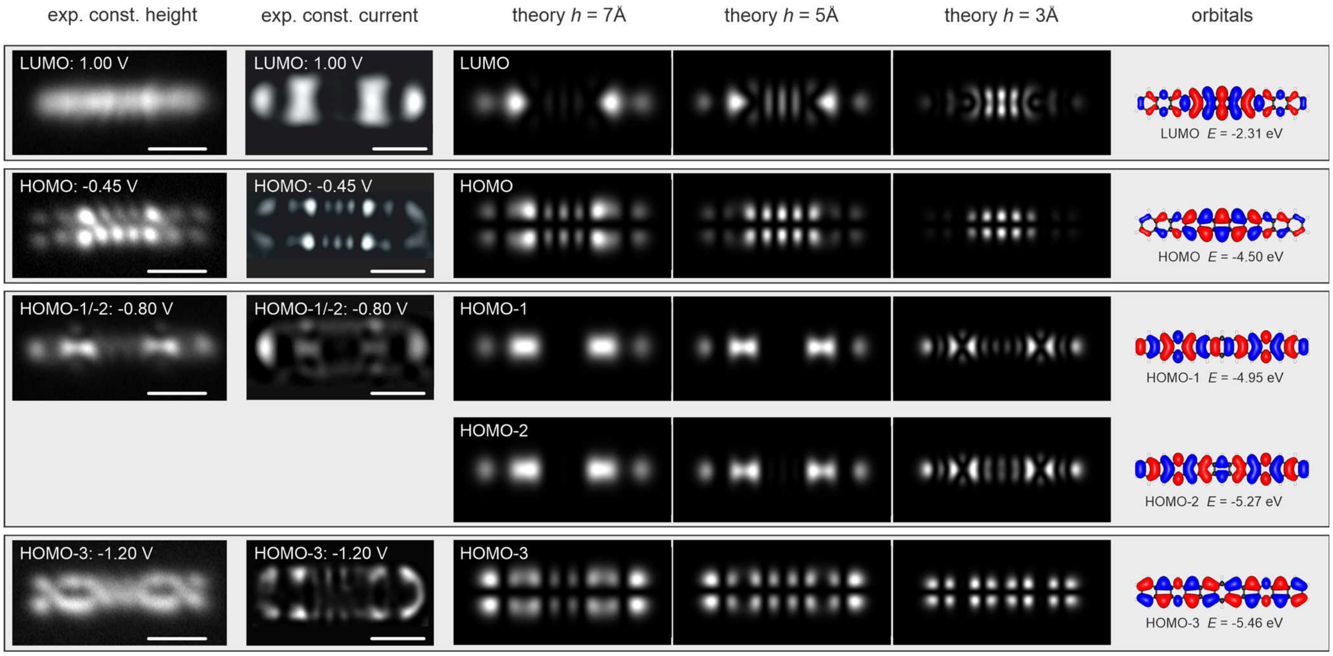

Supplementary Figure 3 Height variation for additional state maps for the hydrogenated tetraazaundecacene **1**. First and second column show the experimental constant height and constant current d*I*/d*V* maps at bias voltages where a state was discernible. Third, fourth and fifth column show the DFT-calculated state maps of HOMO-3 to LUMO at heights 7, 5 and 3 Å. Sixth column shows the corresponding orbital isosurfaces at isovalues ±0.01 a.u. Experimental signatures were assigned to molecular states based on the matching with theory. Scale bars: 1 nm.

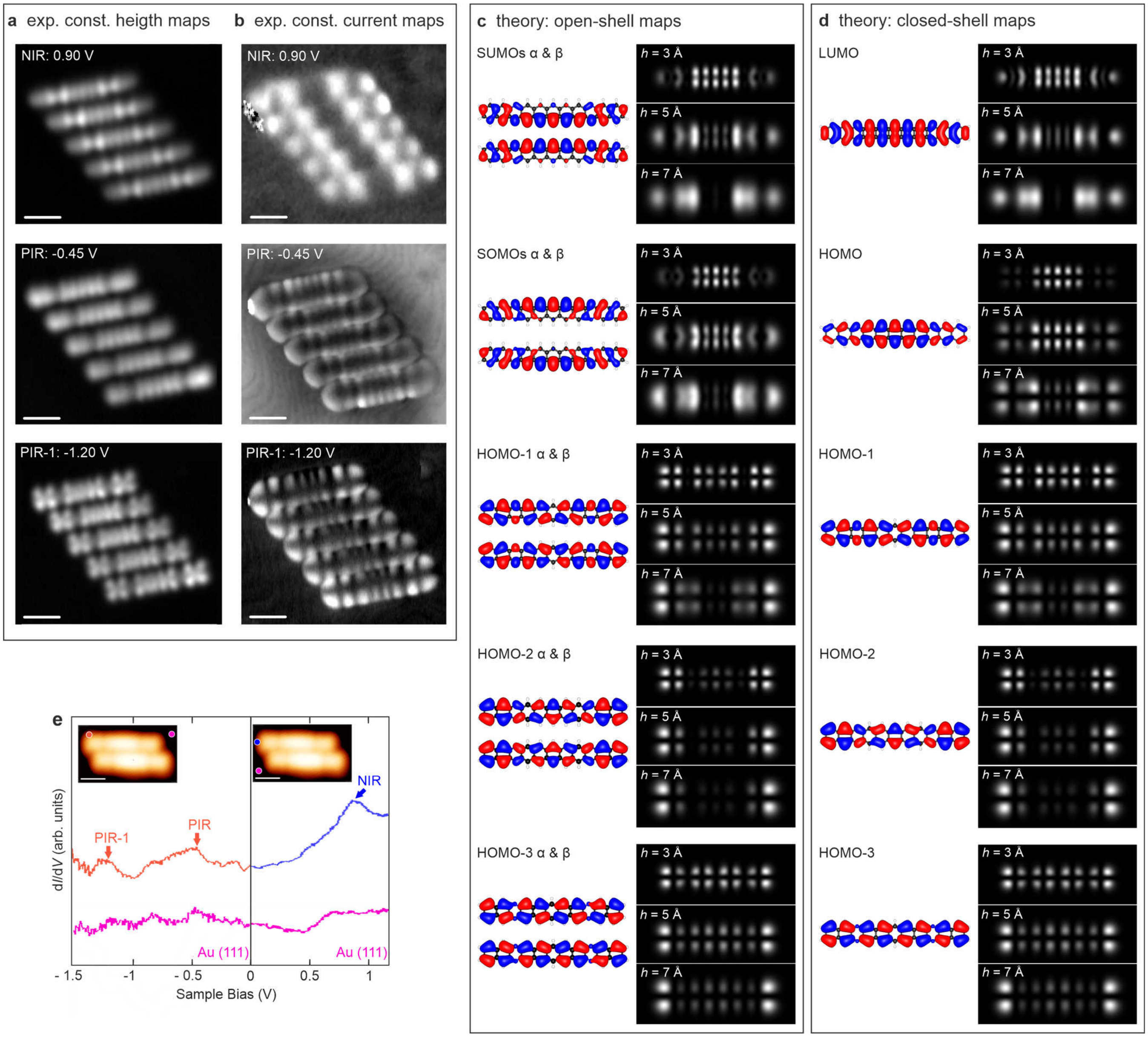

Supplementary Figure 4 Additional state maps for tetraazaundecacene **3**. **a,b** Experimental constant-height and constant current d*I*/d*V* maps at bias voltages where a state was discernible. Scale bars: 1 nm. **c,d** DFT-calculated orbitals and state maps of HOMO-3 to SUMOs/LUMO at heights 3, 5 and 7 Å for the broken-symmetry open-shell singlet (**c**) and the closed-shell singlet solution (**d**). Orbital isosurfaces are plotted at isovalues ±0.01 a.u. **e** d*I*/d*V* spectrum acquired on **3.** The red curves were acquired at the corner position while the blue curves were acquired at the apex of the molecule, as indicated in the constant-current STM image shown in the inset (V_b = -1.5 V, I_t = 200 pA, scale bars = 1 nm). PIR-1, PIR and NIR stand for the second positive, the positive and the negative ion resonances, respectively. Reference d*I*/d*V* spectrum taken on the bare Au(111) surface is depicted in pink in both graphs.

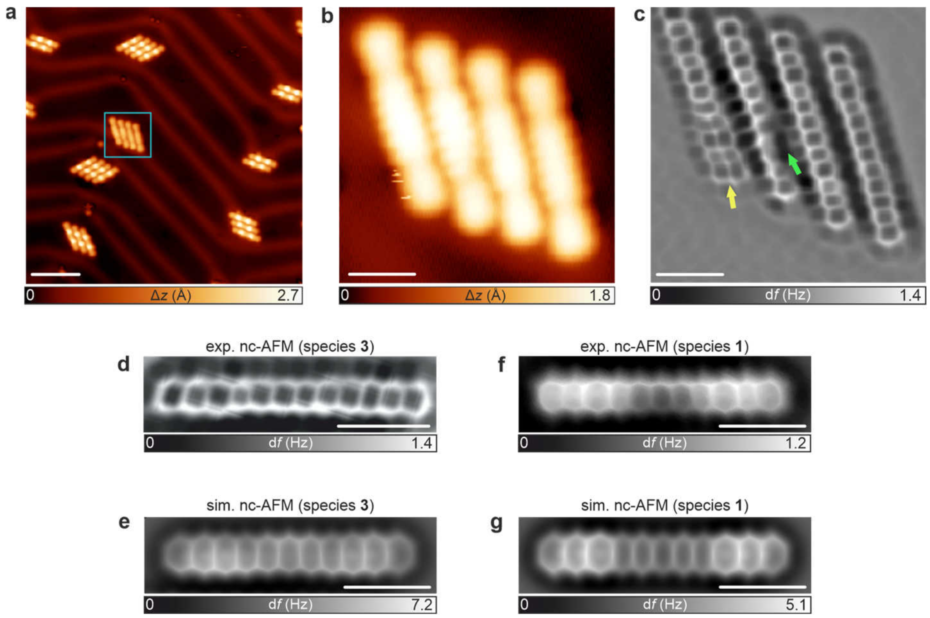

Supplementary Figure 5 STM and nc-AFM images of tetraazaundecacene **3**. **a,b** Overview and zoom-in STM topography images of four tetraazaundecacene molecules acquired after the tip-induced cleavage of the ethano protecting groups. **a** V_b = -0.30 V, It = 70 pA, scale bar = 5 nm. **b** V_b = -0.45 V, I_t = 100 pA, scale bar = 1 nm. **c** Constant-height frequency-shift nc-AFM image of the four tetraazaundecacene molecules shown in **a,b** acquired with a CO-functionalized tip (z offset −60 pm below STM set point: 5 mV, 100 pA). The yellow arrow highlights the dynamic displacement of one of the tetraazaundecacene molecules which occurred during the acquisition of the nc-AFM image. The green arrow indicates a structural modification observed in another tetraazaundecacene molecule which is tentatively assigned to a side effect of the tip-induced cleavage of the ethano protecting groups. **d,e** Constant-height frequency-shift and simulated nc-AFM images based on the DFT equilibrium geometry respectively for **3**. **f,g** Constant-height frequency-shift and simulated nc-AFM images based on the DFT equilibrium geometry respectively for **1** shown for comparison with **3**.

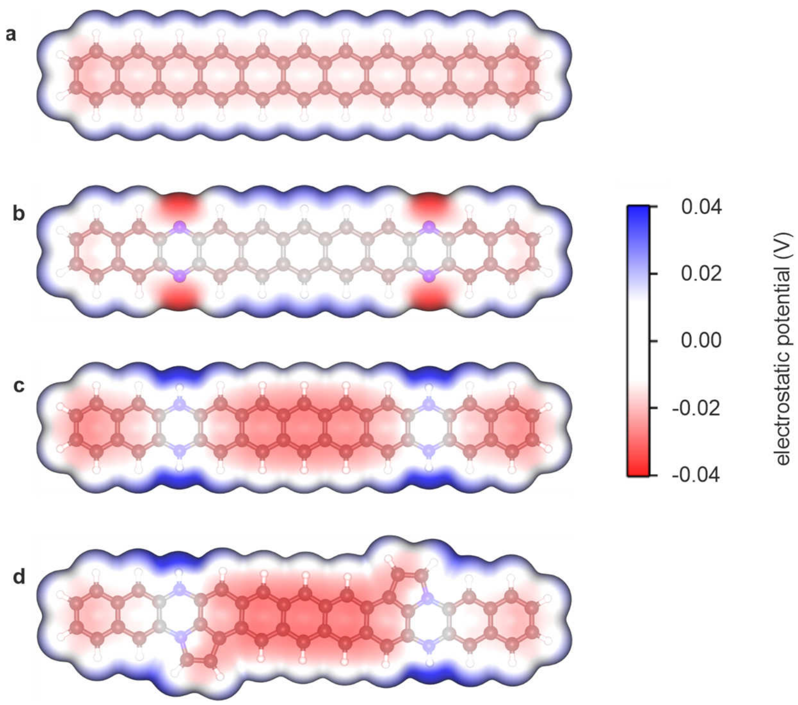

Supplementary Figure 6 Electrostatic potential (ESP) maps for the synthesized compounds. **a** ESP map for pristine undecacene **4**. **b** ESP map for tetraazaundecacene **3**. **c** ESP map for hydrogenated tetraazaundecacene **1**. **d** ESP map for hydrogenated tetraazaundecacene with edge-fused five-membered rings **2**. In all cases, the ESP map is plotted on an electron density isosurface at the isovalues of 0.001 a.u. Only the tetraazaundecacene exhibits positive and negative ESP regions at the periphery, enabling self-assembly on the surface.

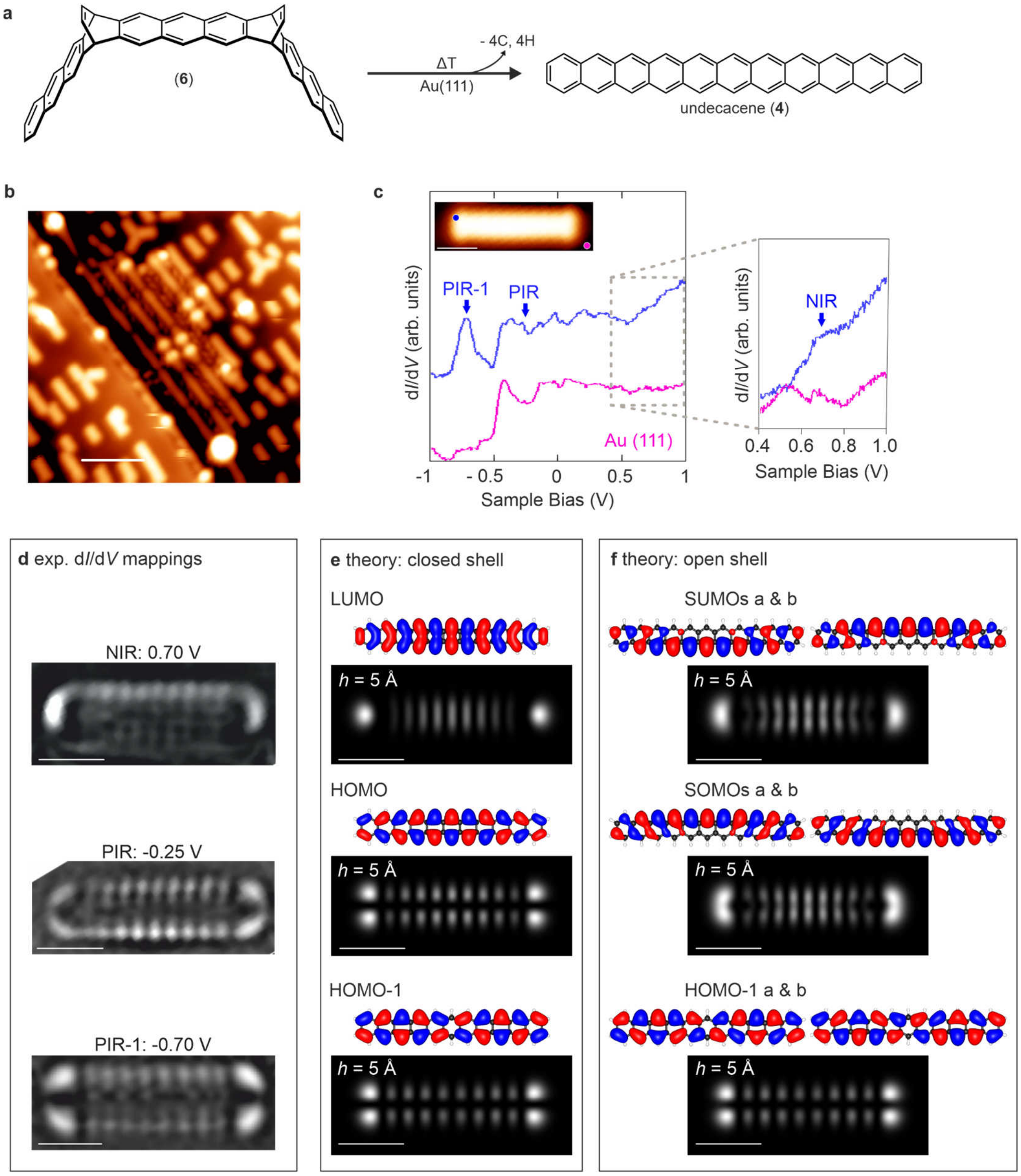

g

acene species	acene length (nm)	%
Anthracene	1.03	18
Tetracene	1.38	24
Pentacene	1.78	21
Hexacene	2.12	2
Heptacene	2.52	8
Octacene	2.80	7
Nonacene	3.07	2
Decacene	3.31	1
Undecacene	3.59	5
Kinked acenes	-	12

Supplementary Figure 7 Synthesis and characterization of undecacene **4**. **a** Scheme showing the on-surface reaction to synthesize undecacene. **b** Overview STM topography image after annealing the sample to 220 ºC. scale bar: 5 nm. **c** dI/dV spectrum acquired on **4** at the position indicated by the blue dot in the inset (inset shows an STM image with V_b = -0.75 V, I_t = 100 pA, scale bar: 1 nm). PIR-1, PIR and NIR stand for the second positive, the positive and the negative ion resonances, respectively. Zoomed-in dI/dV spectrum is taken where an abrupt stepwise change in conductance around 0.7 V is observed. The PIR peak is overlapping the Au(111) surface state and therefore difficult to detect in the point spectrum. Reference dI/dV spectrum taken on the bare Au(111) surface is depicted in pink in both panels. **d** Experimental constant current dI/dV maps of the PIR-1, PIR and NIR signatures of the undecacene**.** scale bars: 1 nm. **e,f** DFT-calculated molecular orbitals (isovalues ±0.01 a.u.) and LDOS maps at the height of 5 Å for the closed shell and the open shell broken-symmetry ground state. **g** Table showing the statistics (out of ~ 800 molecules) of the acene species found on the surface after the annealing step at 220 ºC. The cleavage of the different C(sp^3)-C(sp^2) bonds presents no clear selectivity due to the similarity of C(sp^2) in the C_2H_2 bridge compared to those sp^2-carbon atoms belonging to the backbone. Scanning parameters of the STM images employed to obtain the profile line measurements: V_b = -1 V, I_t = 10 pA.

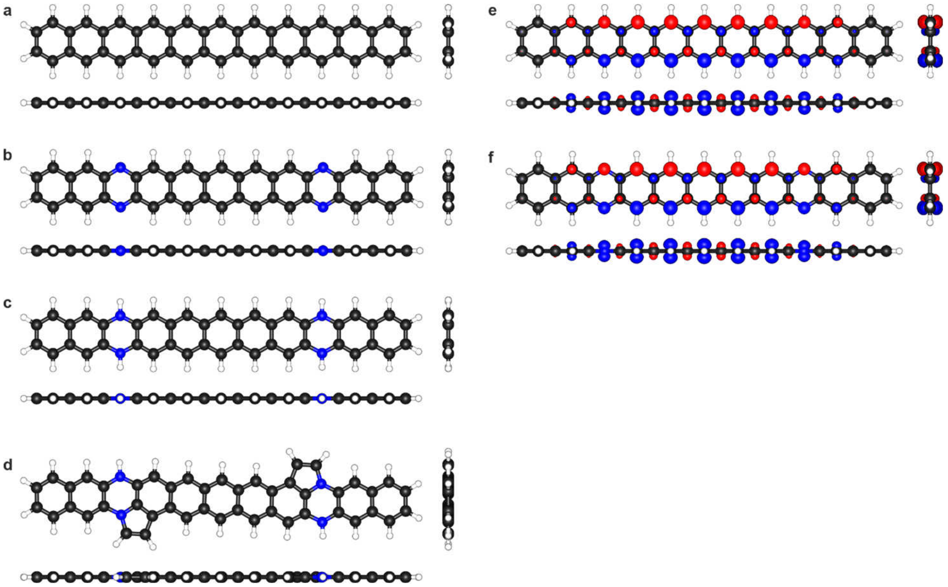

Supplementary Figure 8 Gas phase density functional theory equilibrium geometries and ground state spin densities for the synthesized species at the B3LYP/6-311+G** level of theory. **a** Optimized geometry of undecacene **4**. **b** Optimized geometry of tetraazaundecacene **3**. **c** Optimized geometry of hydrogenated tetraazaundecacene **1**. **d** Optimized geometry of hydrogenated tetraazaundecacene with edge-fused five-membered rings **2**. **e,f** Spin densities of open-shell ground states of undecacene and tetraazaundecacene, respectively. Spin density isosurfaces are plotted at isovalues ±0.01 a.u.

	Undecacene **4**	Tetraazaundecacene **3**	Hydrogenated tetraazaundecacene **1**	Hydrogenated tetraazaundecacene with five-membered rings **2**
Ground state (UB3LYP)	Open-shell singlet (CS +0.48 eV)	Open-shell singlet (CS +0.50 eV)	Closed shell	Closed shell
Ground state spin contamination ⟨S^2⟩	1.53	1.50	-	-
Triplet energy (UB3LYP)	+0.24 eV	+0.22 eV	+0.94 eV	+1.08 eV
H-L gap (RB3LYP)<br>H-L gap (UB3LYP)	0.77 eV<br>1.76 eV	0.72 eV<br>1.73 eV	2.19 eV<br>-	2.30 eV<br>-
<u>Ionization potential</u><br>Koopmans' (RHF)<br>ΔSCF (RB3LYP)<br>ΔSCF (UB3LYP)	 4.94 eV<br>5.14 eV<br>5.63 eV	 5.35 eV<br>5.62 eV<br>6.12 eV	 5.76 eV<br>5.47 eV<br>-	 5.70 eV<br>5.42 eV<br>-
<u>Radical character (UHF w/ sp)</u><br>biradical<br>tetraradical<br>hexaradical<br># unpaired electrons	 0.954<br>0.466<br>0.184<br>3.665	 0.955<br>0.462<br>0.185<br>3.680	 -<br>-<br>-<br>-	 -<br>-<br>-<br>-

Supplementary Table 1 Electronic properties of pristine and the synthesized tetraazaundecacenes. The ground state was determined to be closed-shell (CS) if the spin unrestricted calculation converged to the equivalent solution as the restricted one. Triplet and CS energies w.r.t. to the ground state are given as adiabatic excitation energies. H-L gap denotes HOMO-LUMO (SOMO-SUMO for open-shell cases) gap. The radical characters were calculated using unrestricted Hartree-Fock with spin-projection, as described in the computational methods section.

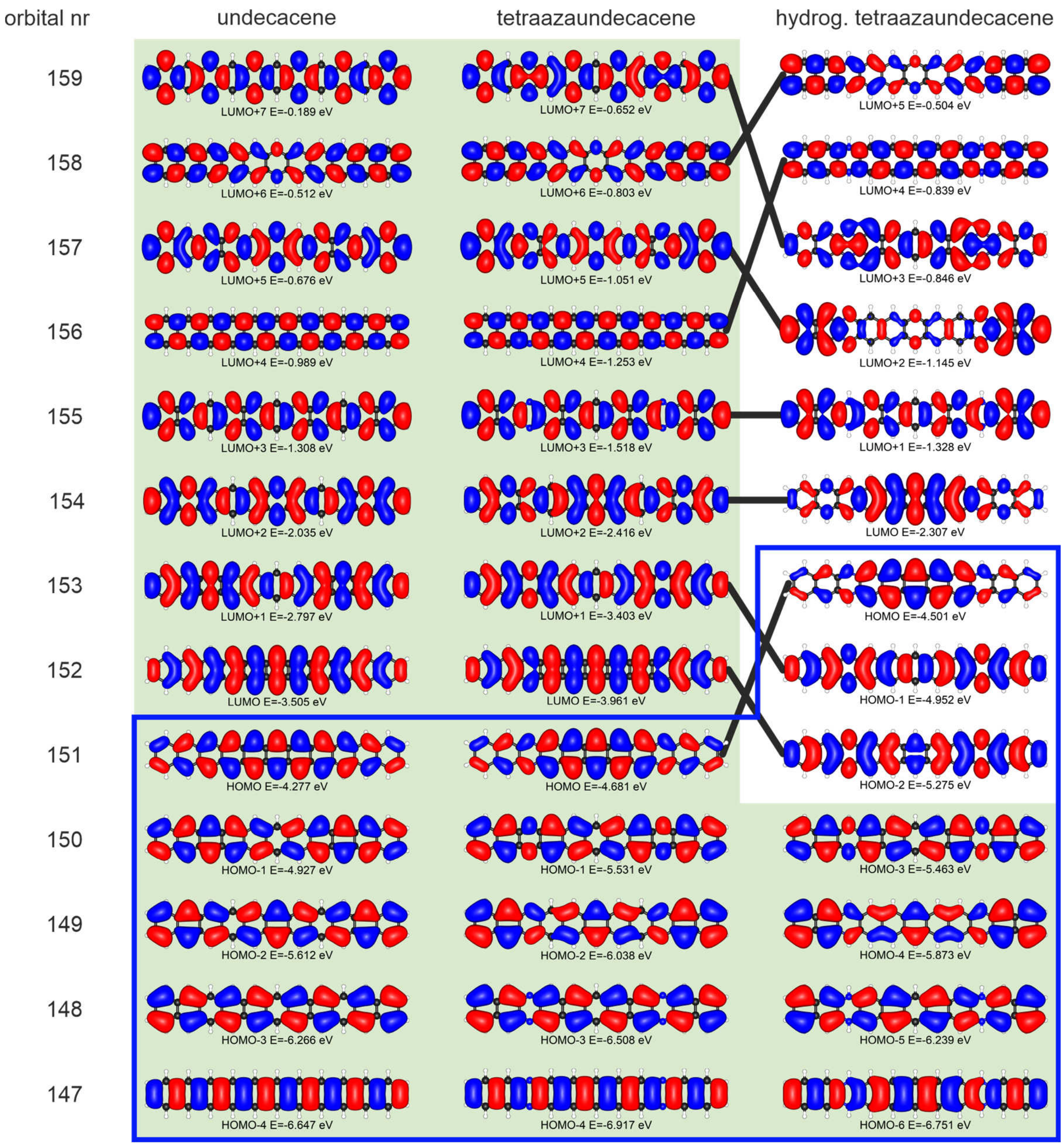

Supplementary Figure 9 Comparison of DFT molecular orbitals for the closed-shell solutions of **4**, **3** and **1**. Orbital number refers to the global ordering in energy of all the orbitals for a given system. The blue border refers to orbitals that are occupied. The green background indicates that the orbital symmetry matches with the pristine undecacene orbitals. Black lines show the symmetry matching for orbitals of **1**, which have a different ordering compared to **4** and **3**. All orbitals calculated at the B3LYP/6-311+G** level of theory and plotted at isovalues ±0.01 a.u.

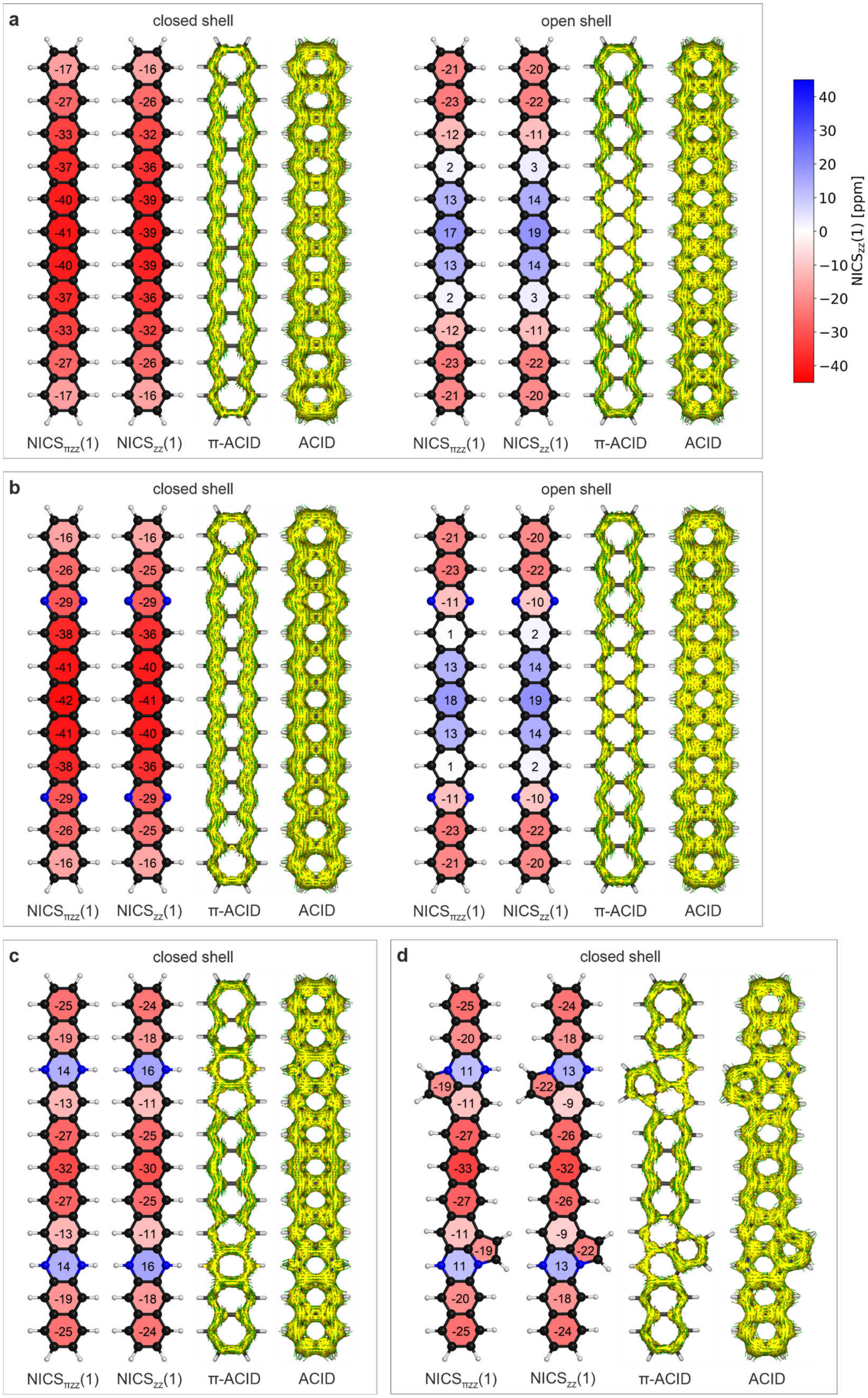

Supplementary Figure 10 Extended results for the NICS and ACID calculations. **a** $NICS_{\pi zz}(1)$ and $NICS_{zz}(1)$ patterns, π-ACID and full ACID plot (left to right) for the closed shell and the open shell solution of undecacene **4**. **b** $NICS_{\pi zz}(1)$ and $NICS_{zz}(1)$ patterns, π-ACID and full ACID plot (left to right) for the closed shell and the open shell solution of tetraazaundecacene **3**. **c** $NICS_{\pi zz}(1)$ and $NICS_{zz}(1)$ patterns, π-ACID and full ACID plot (left to right) for the hydrogenated tetraazaundecacene **1**. **d** $NICS_{\pi zz}(1)$ and $NICS_{zz}(1)$ patterns, π-ACID and full ACID plot (left to right) for the hydrogenated tetraazaundecacene with edge-fused five-membered rings **2**. All ACID plots are showing the isosurface at isovalue 0.05 a.u.

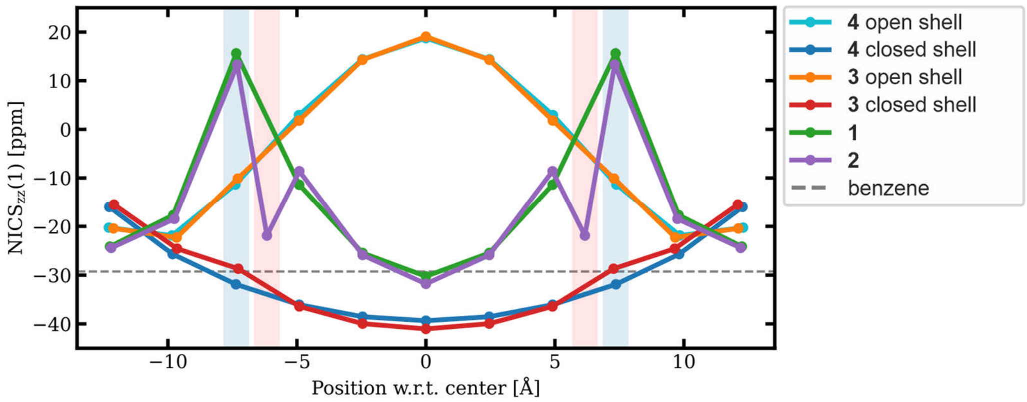

Supplementary Figure 11 NICS$_{zz}$(1) data at the centers of cycles for all the investigated systems together with the value at the center of benzene for comparison. Blue shaded areas represent the center of the cycles containing nitrogen and red shaded area represents the centers of the five-membered rings of **2**.

Supplementary Methods: Computational Details

The adsorption geometries on the Au(111) surface were calculated with the CP2K software package[1] using the PBE exchange-correlation functional[2] together with the DFT-D3 van der Waals scheme proposed by Grimme[3] and norm-conserving GTH pseudopotentials[4]. A TZV2P MOLOPT basis set[5] was used for N, C, H species and a DZVP MOLOPT basis set for the Au species together with a cutoff of 600 Ry for the plane wave basis set. The surface/adsorbate systems were modeled within the repeated slab scheme, with a simulation cell containing 4 atomic layers of Au along the [111] direction and a layer of hydrogen atoms to suppress one of the two Au(111) surface states. 40 Å of vacuum was included in the simulation cell to decouple the system from its periodic replicas. The gold surface was modeled by a supercell of 41.27 Å × 40.85 Å corresponding to 224 surface units. The adsorption geometry was optimized by keeping the positions of the two bottom layers of the slab fixed to the ideal bulk coordinates, while all the other atoms were relaxed until forces were lower than 0.005 eV/Å.

The eigenvalue self-consistent GW calculations[6] were performed with the CP2K code based on the isolated molecular geometry and the adsorption conformation. The calculation was performed based on the DFT PBE wavefunctions using the GTH pseudopotentials and analytic continuation with a two-pole model. The aug-DZVP basis set from Wilhelm et al.[7] was used. To account for screening by the metal surface, we applied the image charge model by Neaton et al.[8], and to determine the image plane position w.r.t. the molecular geometry, we used a distance of 1.42 Å between the image plane and the first surface layer, as reported by Kharche et al.[9]

Scanning tunnelling microscopy (STM) images were simulated within Tersoff-Hamann approximation[10] based on the Kohn-Sham orbitals. The orbitals were extrapolated to the vacuum region in order to correct the wrong decay of the charge density due to the localized basis set[11]. The Probe Particle model[12] was used to simulate the non-contact AFM images.

The gas-phase electronic structure, local density of states (LDOS) maps and aromaticity calculations were performed with the Gaussian software package[13]. The B3LYP functional was used in the spin-restricted and unrestricted formalism (respectively for the closed and open-shell states). The 6-311G** basis set was used for geometry optimizations, while single point and aromatic properties were calculated using the 6-311+G** basis set. The radical characters were calculated based on the natural orbital occupation numbers with the unrestricted Hartree-Fock method using Yamaguchi's spin projection scheme[14,15] and the number of unpaired electrons was found by the expression proposed by Head-Gordon et al.[16] The $NICS_{zz}(1)$[17,18] was calculated with the GIAO-B3LYP method as the negative of the magnetic shielding tensor component perpendicular to each local cycle evaluated at height 1 Å away from the center. The $NICS_{\pi zz}(1)$ was calculated using the canonical molecular orbital (CMO) natural chemical shielding analysis (NCS)[19] within the NBO program suite[20]. The $NICS_{\pi zz}(1)$ for the open-shell cases was calculated by subtracting the $NICS_{\sigma zz}(1)$ results of the closed-shell calculation from the open-shell $NICS_{zz}(1)$ as the CMO-NCS analysis does not support open-shell calculations. The anisotropy of the induced current density (ACID)[21] calculations were performed with the CSGT method[22]. The π orbitals were detected with the Multiwfn software[23].

The AiiDAlab platform[24] was used to perform the calculations for the molecules adsorbed on Au(111).

Supplementary Methods: Precursor Synthesis and Characterization

1. General methods and materials

1.1 Synthesis and characterization

Reagents for synthesis were purchased from Wako, Nacalai Tesque, and Sigma Aldrich, and were reagent-grade quality, obtained commercially, and used without further purification. For spectral measurements, spectral-grade solvents were purchased from Nacalai Tesque. Unless stated otherwise, column chromatography was carried out on silica gel 60N (Kanto Chemical, 40–50 µm). Analytical thin layer chromatography (TLC) was performed on Art. 5554 (Merck, KGaA). Melting points (mp) were measured with an SRS MPA100. Fourier-trnasform-infrared (FT-IR) spectra were recorded on a JASCO FT/IR-4200 and are reported as wavenumbers ν in cm^{-1} with band intensities indicated as s (strong), m (medium), w (weak). ^{1}H NMR (400, 500, and 600 MHz) and ^{13}C NMR (126 and 151 MHz) spectra were recorded (as indicated) either on a JEOL JNM-ECX 600 spectrometer and are reported as chemical shifts (δ) in ppm relative to TMS (δ = 0). Broad peaks are marked as br. High resolution MS was performed on a MALDI–TOF-MS (Bruker Autoflex II) or MALDI–Spiral-TOF-MS (JEOL spiralTOF, JMS-S3000) or ESI–TOF-MS (JEOL AccuTOF JMS-T100LC). X-ray crystallographic data were recorded at 90 K (compound 1) or 133 K (compound 2) on a Bruker APEX II X-Ray diffractometer equipped with a large area CCD detector by using graphite monochromated Mo-Kα radiation (λ = 0.71073 Å).

1.2 Abbreviations

AcOH: Acetic acid; Ar: Argon; *n*-BuLi: *n*-Butyllithium; DCM: Dichloromethane; DIPEA: *N*,*N*-Diisopropylethylamine; DMSO: Dimethyl sulfoxide; EtOAc: Ethyl acetate; HRMS: High resolution mass spectrometry; LAH: Lithium aluminum hydride; MALDI–TOF: Matrix assisted laser desorption ionization-time of flight; mp: Melting point; r.t.: Room temperature; MeOH: Methanol; NMO: *N*-Methylmorpholine *N*-oxide; TFAA: Trifluoroacetic anhydride; THF: Tetrahydrofuran; TLC: Thin layer chromatography; TMS: Tetramethylsilane.

2. Synthetic procedures

2-1. Synthesis of nitrogen-substituted undecacene precursor

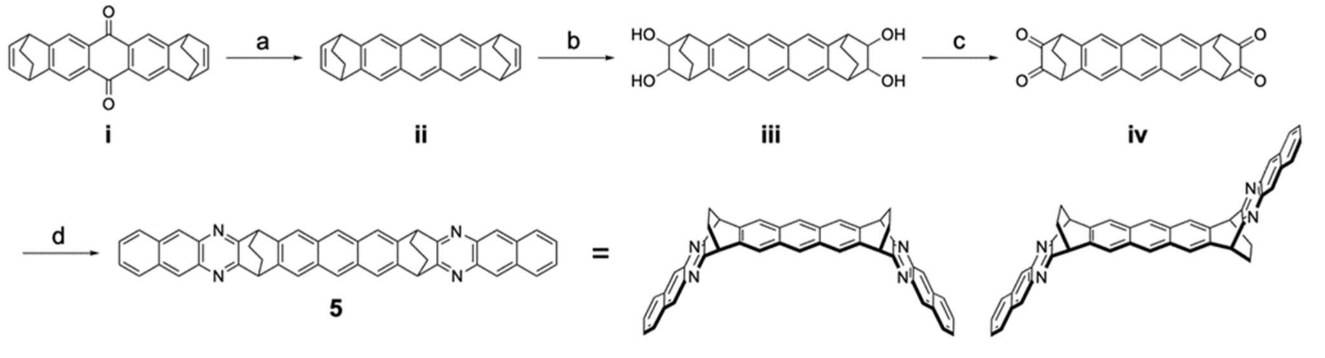

Supplementary Figure 12. Synthetic route for N-substituted undecacene precursor. Reaction conditions: a) 1) LAH, THF, reflux, 1 h, 2) 6 M HCl aq., reflux, 1 h, 3) LAH, THF, reflux, 1 h, 4) 6 M HCl aq., reflux, 1 h, 39%; b) OsO_4 (10% microcapsule), NMO, acetone, r.t., 4 d, 66%; c) DMSO, TFAA, DIPEA, DCM, –80 ºC, 6 h, 98%; d) 2,3-diaminonaphthalene, AcOH, 60 ºC, 1 h, 70%.

Compound i: was synthesized and its physical properties were identical with those reported in the literature.[25]

Compound ii: Under Ar atmosphere, a suspension of compound **i** (506 mg, 1.39 mmol) in dry-THF (50 mL) was cooled to 0 ºC with ice-water bath. LAH (545 mg, 14.3 mmol) was added to the mixture portionwise. The reaction mixture was heated to reflux for 1 h. After cooling to 0 ºC, the reaction was slowly quenched with 6 M HCl aq. (35 mL). The solution was heated to reflux for 1 h. After cooling to 0 ºC, the resulting precipitate was collected with filtration and washed with water and MeOH. Under Ar atmosphere, the suspension of crude products in dry-THF (50 mL) was cooled to 0 ºC with ice-water bath. LAH (552 mg, 14.5 mmol) was added to the mixture portionwise. The reaction mixture was heated to reflux for 1 h. After cooling to 0 ºC, the reaction was slowly quenched with 6 M HCl aq. (35 mL). The solution was heated to reflux for 1 h. After cooling to 0 ºC, the precipitation was collected with filtration and washed with water and MeOH. The crude

products were purified with silica gel column chromatography ($CHCl_3$/hexane = 1:1, R_f = 0.8) to give compound **ii** as a pale yellow solid (180 mg, 0.539 mmol, 39%). mp: 270–280 °C (decomp); ^{1}H NMR (400 MHz, $CDCl_3$): δ 8.20 (s, 2H), 7.64 (s, 4H), 6.58–6.56 (m, 4H), 4.00 (s, 4H), 1.67–1.62 (m, 8H); ^{13}C NMR (151 MHz, $CDCl_3$): δ 141.88, 135.14, 130.56, 124.79, 119.71, 40.03, 26.17; IR (KBr): 3445 cm^{-1} (br w), 3046 cm^{-1} (m), 3000 cm^{-1} (m), 2944 cm^{-1} (s), 2902 cm^{-1} (m), 2863 cm^{-1} (s), 1802 cm^{-1} (w), 1656 cm^{-1} (s), 1604 cm^{-1} (s), 1441 cm^{-1} (s), 1349 cm^{-1} (s), 1330 cm^{-1} (s), 1308 cm^{-1} (s), 1160 cm^{-1} (m), 1134 cm^{-1} (m), 1105 cm^{-1} (m), 969 cm^{-1} (w), 902 cm^{-1} (s), 832 cm^{-1} (s), 747 cm^{-1} (s), 689 cm^{-1} (s); HRMS (m/z): $[M]^+$ calcd. for $C_{26}H_{22}$, 334.1716; found, 334.1717.

Compound iii: To a suspension of compound **ii** (229 mg, 0.686 mmol) in acetone (250 mL), NMO (0.793 g, 6.77 mmol) and OsO_4 (10% microcapsule, 210 mg, 0.0826 mmol) were added. The solution was stirred for 4 days at r.t.. Then, saturated $Na_2S_2O_4$ aq. (50 ml) was added to the mixture, and stirred for 10 minutes. After removing acetone under reduced pressure, the crude products were extracted with EtOAc, and then the combined organic phase was washed with brine and dried over Na_2SO_4. The solution was removed by evaporation to give yellow solids. The crude products were suspended with a small amount of $CHCl_3$, and then the resulting suspension was filtered, and subsequently washed with MeOH to give compound **iii** as a white solid (183 mg, 0.455 mmol, 66%, R_f = 0.6 with $CHCl_3$/MeOH = 10 : 1). mp: 260–270 °C (decomp); ^{1}H NMR (400 MHz, $CDCl_3$): δ 8.34 (s, 2H), 7.68 (s, 4H), 4.40 (s, br, 4H), 4.00 (s, 4H), 3.05 (s, 4H), 1.77–1.76 (m, 4H), 1.40–1.36 (m, 4H); ^{13}C NMR (151 MHz, DMSO-d_6): δ 138.91, 131.02, 124.44, 123.67, 69.25, 40.46, 23.03; IR (KBr): 3384 cm^{-1} (br s), 3025 cm^{-1} (m), 2927 cm^{-1} (s), 2868 cm^{-1} (m), 1732 cm^{-1} (m), 1602 cm^{-1} (m), 1493 cm^{-1} (m), 1451 cm^{-1} (m), 1068 cm^{-1} (s), 909 cm^{-1} (m), 821 cm^{-1} (m), 756 cm^{-1} (m), 697 cm^{-1} (s); HRMS (m/z): $[M]^+$ calcd. for $C_{26}H_{26}O_4$, 402.1826; found, 402.1829.

Compound iv: In 300 mL three-neck flask, dry-CH_2Cl_2 (70 m) and dry-DMSO (1.4 ml, 20 mmol) were mixed under Ar atmosphere. After cooling to –80 ºC, TFAA (1.4 ml, 10.0 mmol) was slowly added to the solution over 20 min. After stirring for 1 h at –80 ºC, compound **iii** (129 mg, 0.333 mmol) in a mixture of dry-CH_2Cl_2 (45 ml) and dry-DMSO (7 ml) was slowly added to the solution over 3 h. The solution was stirred for 2 h at –80 ºC, and then distilled-DIPEA (3.5 ml, 20 mmol) was added. After stirring for 1 h, the solution was warmed to r.t. and stirred for 1 h. The solution was quenched with 3 M HCl aq. and extracted with CH_2Cl_2. The organic layer was washed with water and brine, and dried over Na_2SO_4. The organic solvent was removed under reduced pressure. The crude product was reprecipitated from DCM-hexane three times and heated with glass tube oven (50 ºC for 1 h) to give compound **iv** as an orange solid (124 mg, 0.315 mmol, 98%). mp: >300 °C; ^{1}H NMR (400 MHz, $CDCl_3$): δ 8.42 (s, 2H), 7.91 (s, 4H), 4.18 (s, 4H), 2.40–2.38 (m, 4H), 2.17–2.16 (m, 4H); ^{13}C NMR (151 MHz, $CDCl_3$): δ 192.10, 133.29, 131.93, 126.47, 125.33, 53.06, 22.98; IR (KBr): 3452 cm^{-1} (br m), 2945 cm^{-1} (m), 2871 cm^{-1} (m), 1731 cm^{-1} (s), 1672 cm^{-1} (w), 1638 cm^{-1} (w), 1465 cm^{-1} (w), 1449 cm^{-1} (w), 1227 cm^{-1} (m), 1081 cm^{-1} (m), 976 cm^{-1} (w), 909 cm^{-1} (m); HRMS (m/z): $[M+Na]^+$ calcd. for $C_{26}H_{18}O_4$, 417.1097; found, 417.1095.

7,11,20,24-Tetrahydro-7,24:11,20-diethano-6,12,19,25-tetraazaundecacene (Compound 5): Compound **iv** (79 mg, 0.198 mmol) and 2,3-diaminonaphthalene (31.3 mg, 0.594 mmol) were dissolved in a mixture of AcOH (35 ml) and toluene (52 ml). Then, the solution was heated to 60 ºC for 1 h. After cooling to r.t., the organic solvent was filtrated to give compound **5** as a white solid (89 mg, 0.139 mmol, 70%). mp: >300 °C; ^{1}H NMR (400 MHz, $CDCl_3$): δ 8.53 (s, 4H), 8.35 (s, 2H), 8.06–8.04 (m, 4H), 7.97 (s, 4H), 7.53–7.51 (m, 4H), 4.74 (s, 4H), 2.20 (m, 8H); ^{13}C NMR (151 MHz, $CDCl_3$): δ 157.92, 138.32, 137.90, 133.37, 131.21, 128.41, 127.06, 126.53, 125.84, 123.00, 46.55, 25.50; IR (KBr): 3408 cm^{-1} (br m), 3049 cm^{-1} (s), 2956 cm^{-1} (s), 2869 cm^{-1} (s), 1735 cm^{-1} (m), 1672 cm^{-1} (m), 1604 cm^{-1} (m), 1467 cm^{-1} (m), 1446 cm^{-1} (m), 1343 cm^{-1} (s), 1310 cm^{-1} (s), 1246 cm^{-1} (s), 1173 cm^{-1} (s), 1110 cm^{-1} (s), 973 cm^{-1} (m), 881 cm^{-1} (m), 745 cm^{-1} (s); HRMS (m/z): $[M]^+$ calcd. for $C_{46}H_{30}N_4$, 638.2465; found, 638.2468.

2-2. Synthesis of undecacene precursor

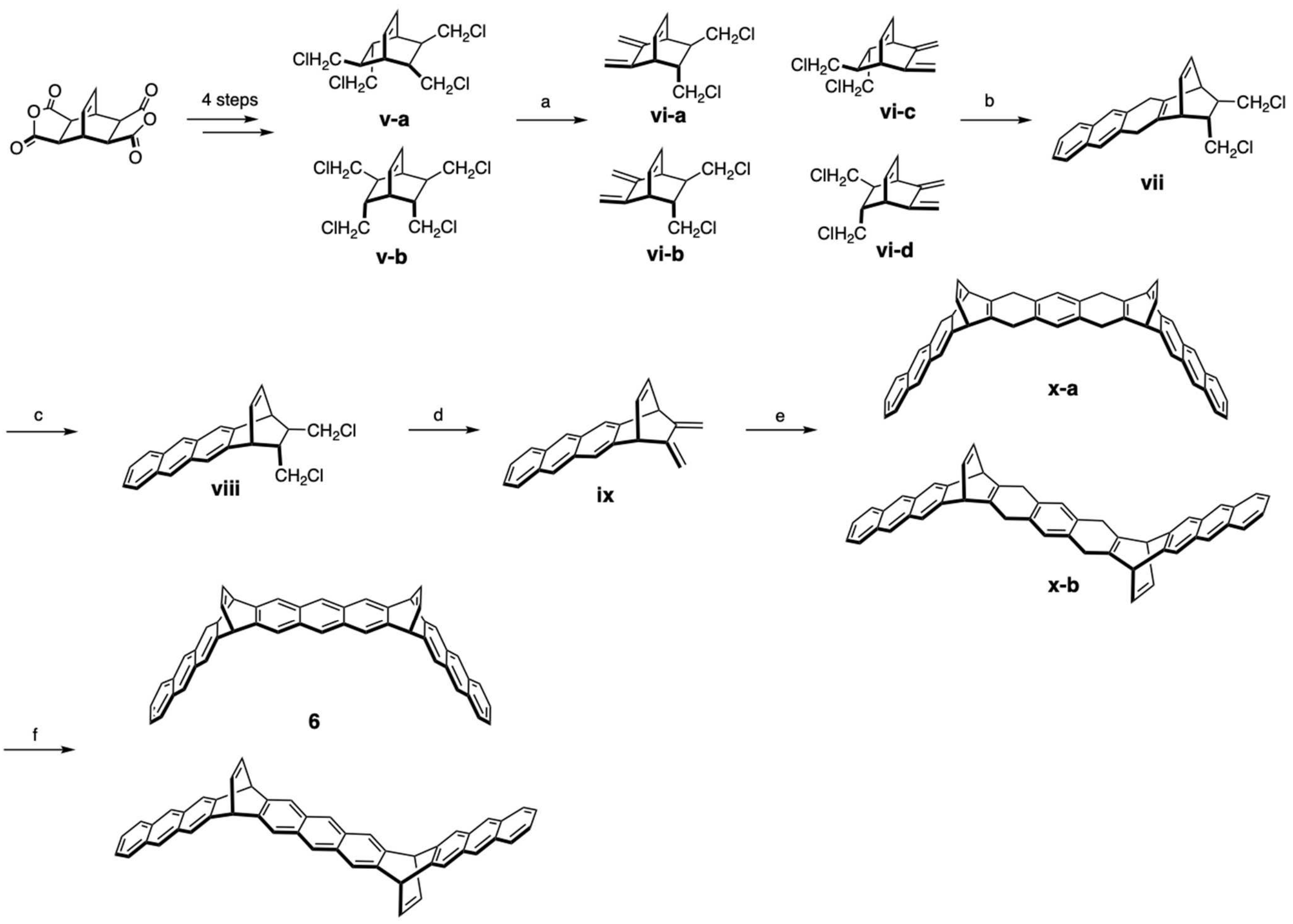

Supplementary Figure 13. Synthetic route for undecacene precursor. Reaction conditions: a) *t*-BuOK, THF, 50 ºC, 2 h, 99%; b) *n*-BuLi, 2,3-dibromonaphthalene, toluene, –10 ºC to r.t., overnight, 100%; c) *p*-chloranil, K_2CO_3, toluene, reflux, 12 h, 94%; d) *t*-BuOK, THF, reflux, 13 h, 87%; e) *n*-BuLi, 1,2,4,5-tetrabromobenzene, toluene, –10 ºC to r.t., overnight, 76%; f) *p*-chloranil, K_2CO_3, toluene, reflux, 20 h, 51%.

Compound v: was synthesized from bicyclo[2.2.2]oct-7-ene-2,3,5,6,-tetracarboxylic dianhydride and its physical properties were identical with those reported in the literature.[26]

Compound vi: *t*-BuOK (5.00 g, 44.6 mmol) was added portionwise to a THF solution (150 ml) of **v** (4.5 g, 15 mmol) under Ar atmosphere. Then, the reaction mixture was heated at 50 °C for 2 h. After cooling to r.t., the reaction was quenched by addition of water, and the solvent was concentrated under reduced pressure. Then, 2 M HCl aq. and dichloromethane was added to the mixture, and the combined organic phase was washed with water and brine. After dried over Na_2SO_4, the solvent was removed under reduced pressure, and the residue was purified by silica gel column chromatography (hexane/DCM = 9 : 1, R_f = 0.38) to afford compound **vi** (3.4 g, 99%) as an oil. ^{1}H NMR (400 MHz, $CDCl_3$): δ 6.44–6.40 (m, 1H), 6.25–6.22 (m, 1H), 5.39 (s, 1H), 5.26 (s, 1H), 4.93 (s, 1H), 4.90 (s, 1H), 3.60–3.56 (m, 1H), 3.40–3.27 (m, 5H), 1.69–1.65 (m, 1H), 1.54–1.49 (m, 1H); ^{13}C NMR (126 MHz, $CDCl_3$): δ 144.81, 141.15, 134.00, 131.20, 107.25, 104.69, 48.12, 47.33, 47.26, 45.24, 44.40, 43.85; IR (KBr): 1432 cm^{-1} (m), 1302 cm^{-1} (m), 1267 cm^{-1} (m), 893 cm^{-1} (s), 831 cm^{-1} (m), 723 cm^{-1} (s), 689 cm^{-1} (s); HRMS (m/z): [M]$^+$ calcd. for $C_{12}H_{14}Cl_2$, 228.0473; found, 228.0470.

Compound vii: A toluene (44 ml) solution of *n*-BuLi (6.2 ml, 1.6. M in hexane) was added dropwise to a toluene solution (320 ml) of **vi** (3.4 g, 15 mmol) and 2,3-dibromonaphthalene (2.0 g, 7.0 mmol) at –10 °C over 1.5 h under Ar atmosphere. After stirring for 30 min at –10 °C, the reaction mixture was gradually warmed to r.t., and was stirred overnight. Then, the reaction was quenched by adding MeOH, and the resulting mixture was concentrated under reduced pressure. The residue was purified by silica gel column chromatography (hexane/DCM =

5 : 1, R_f = 0.33) to afford **9** (2.7 g, 100%) as a viscous oil. Due to its susceptibility to oxidation, the compound was used for the next reaction without further purification. ^{1}H NMR (400 MHz, $CDCl_3$): δ 7.75–7.73 (m, 2H), 7.64–7.63 (m, 2H), 7.40–7.37 (m, 2H), 6.60 (t, J = 7.5 Hz, 1H), 6.39 (t, J = 6.3 Hz, 1H), 3.80–3.68 (m, 5H), 3.60–3.61 (m, 1H), 3.52–3.45 (m, 2H), 3.31–3.26 (m, 1H) , 3.18–3.14 (m, 1H), 1.63–1.59 (m, 1H), 1.59–1.50 (m, 1H); ^{13}C NMR (151 MHz, $CDCl_3$): δ 136.16, 135.58, 133.30, 132.68, 132.19, 127.10, 127.07, 126.83, 125.31, 49.23, 48.66, 48.38, 43.57, 43.26, 33.57, 31.77; IR (KBr): 3430 cm^{-1} (br m), 3052 cm^{-1} (s), 2951 cm^{-1} (s), 2854 cm^{-1} (s), 2814 cm^{-1} (m), 1600 cm^{-1} (m), 1506 cm^{-1} (m), 1439 cm^{-1} (s), 1423 cm^{-1} (s), 1324 cm^{-1} (s), 1296 cm^{-1} (s), 1266 cm^{-1} (s), 1151 cm^{-1} (w), 1102 cm^{-1} (w), 1012 cm^{-1} (w), 862 cm^{-1} (s), 747 cm^{-1} (s), 718 cm^{-1} (s), 696 cm^{-1} (s); HRMS (m/z): $[M]^+$ calcd. for $C_{22}H_{20}Cl_2$, 354.0949; found, 354.0942.

Compound viii: The suspension of **vii** (2.7 g, 7.5 mmol,), *p*-chloranil (1.8 g, 7.5 mmol) and K_2CO_3 (3.6 g, 27 mmol), in dry-toluene (700 ml) was heated to reflux for 12 h under Ar atmosphere. Then, hot-reaction mixture was filtered with hot-toluene. After cooling to r.t., the residue wad diluted with toluene and then washed with 10% NaOH aq., water and dried over Na_2SO_4. The organic phase was concentrated under reduced pressure. The filtrate was purified by silica gel column chromatography (hexane/DCM = 2 : 1, R_f = 0.55) to afford **viii** as a white solid (2.5 g, 94%). mp: 130–135 °C; ^{1}H NMR (400 MHz, $CDCl_3$): δ 8.35 (d, J = 6.3 Hz, 2H), 8.00–7.98 (m, 2H), 7.78 (d, J = 16.0 Hz, 2H), 7.47–7.44 (m, 2H), 6.75 (t, J = 7.0 Hz, 1H), 6.57 (t, J = 7.0 Hz, 1H), 4.19–4.18 (m, 2H), 3.58–3.55 (m, 1H), 3.50–3.46 (m, 1H), 3.38–3.34 (m, 1H), 2.92 (t, 10.0 Hz, 1H), 1.85–1.81 (m, 1H), 1.72–1.68 (m, 1H); ^{13}C NMR (126 MHz, $CDCl_3$): δ 140.27, 137.21, 136.11, 133.07, 131.6, 1301.57, 130.93, 130.62, 128.04, 127.99, 125.78, 125.76, 125.15, 125.13, 123.08, 120.89, 48.38, 48.31, 47.61, 46.63, 42.51, 42.18; IR (KBr): 3043 cm^{-1} (m), 2955 cm^{-1} (m), 2942 cm^{-1} (m), 2896 cm^{-1} (m), 1812 cm^{-1} (w), 1624 cm^{-1} (w), 1539 cm^{-1} (w), 1443 cm^{-1} (s), 1434 cm^{-1} (s), 1347 cm^{-1} (m), 1297 cm^{-1} (s), 1276 cm^{-1} (s), 1265 cm^{-1} (s), 1207 cm^{-1} (w), 1155 cm^{-1} (w), 1115 cm^{-1} (w), 1078 cm^{-1} (w), 1060 cm^{-1} (w), 1005 cm^{-1} (w), 985 cm^{-1} (w), 955 cm^{-1} (m), 901 cm^{-1} (s), 744 cm^{-1} (s); HRMS (m/z): $[M]^+$ calcd. for $C_{22}H_{18}Cl_2$, 352.0786; found, 352.0784.

Compound ix: *t*-BuOK (1.5 g, 13 mmol) was added portionwise to a THF solution (42 ml) of **viii** (1.0 g, 2.8 mmol) under Ar atmosphere. Then, the reaction mixture was heated to reflux for 13 h. After cooling to r.t., the reaction was quenched by addition of water, and the solvent was concentrated under reduced pressure. 2 M HCl aq. and DCM was added to the mixture, and the combined organic phase was washed with water and brine. After dried over Na_2SO_4, the solvent was removed under reduced pressure, and the residue was purified by silica gel column chromatography (hexane/DCM = 10 : 1, R_f = 0.33) to afford **ix** (690 mg, 2.46 mmol. 87%) as a white solid. mp: 170–183 °C; ^{1}H NMR (400 MHz, $CDCl_3$): δ 8.32 (s, 2H), 7.97–7.95 (m, 2H), 7.77 (s, 2H), 7.43–7.41 (m, 2H), 6.70–6.67 (m, 2H), 5.31 (s, 2H), 5.10 (s, 2H), 4.62–4.61 (m, 2H); ^{13}C NMR (126 MHz, $CDCl_3$): δ 143.85, 139.45, 134.42, 131.47, 130.98, 128.00, 125.70, 124.93, 120.47, 104.86, 51.30; IR (KBr): 3052 cm^{-1} (m), 2976 cm^{-1} (m), 2923 cm^{-1} (m), 2314 cm^{-1} (w), 1925 cm^{-1} (w), 1805 cm^{-1} (w), 1670 cm^{-1} (w), 1624 cm^{-1} (m), 1440 cm^{-1} (m), 1415 cm^{-1} (w), 1333 cm^{-1} (m), 1288 cm^{-1} (m), 1257 cm^{-1} (w), 1226 cm^{-1} (w), 1172 cm^{-1} (w), 1101 cm^{-1} (w), 956 cm^{-1} (m), 895 cm^{-1} (s), 875 cm^{-1} (s), 859 cm^{-1} (s), 790 cm^{-1} (s), 741 cm^{-1} (s), 716 cm^{-1} (m), 667 cm^{-1} (s), 632 cm^{-1} (m); HRMS (m/z): $[M]^+$ calcd. for $C_{22}H_{16}$, 280.1252; found, 280.1252.

Compound x: A toluene (250 ml) solution of *n*-BuLi (20 ml, 1.6. M in hexane, 31 mmol) was added dropwise to a toluene solution (350 ml) of **11** (1.7 g, 6.0 mmol) and 1,2,4,5-tetrabromobenzene (1.2 g, 3.0 mmol) at –10 °C over 65 min under Ar atmosphere. After stirring for 1 h at –10 °C, the reaction mixture was gradually warmed to r.t., and was stirred overnight. Then, the reaction was quenched by adding MeOH, and the resulting mixture was filtrated. The filtrate was purified by reprecipitation with chloroform and MeOH to afford a **x** (2.9 g, 76%). mp: >300 °C;); ^{1}H NMR (500 MHz, $CDCl_3$): δ 8.17 (s, 4H), 7.90–7.88 (m, 4H), 7.58 (s, 4H), 7.37–7.35 (m, 4H), 6.90–6.88 (m, 4H), 6.85 (s, 2H), 4.66–4.65 (m, 4H), 3.58–3.53 (m, 8H); ^{13}C NMR (126 MHz, $CDCl_3$): δ 142.64, 138.55, 137.71, 131.55, 131.42, 130.16, 128.48, 127.85, 125.23, 124.63, 118.56, 51.18, 31.74; IR (KBr): 3421 cm^{-1} (br w), 3048 cm^{-1} (m), 2999 cm^{-1} (m), 2959 cm^{-1} (m), 2854 cm^{-1} (br m), 2812 cm^{-1} (m), 1508 cm^{-1} (s), 1436 cm^{-1} (s), 1319 cm^{-1} (w), 1164 cm^{-1} (w), 1084 cm^{-1} (w), 952 cm^{-1} (w), 895 cm^{-1} (s), 862 cm^{-1} (s); HRMS (m/z): $[M]^+$ calcd. for $C_{50}H_{34}$, 634.2655; found 634.2648.

7,11,20,24-tetrahydro-7,24:11,20-dietheno-undecacene (Compound 6): A suspension of **x** (115 mg, 0.181 mmol,), *p*-chloranil (440 mg, 1.79 mmol) and K_2CO_3 (850 mg, 6.15 mmol) in dry-toluene (170 ml) was heated to reflux for 20 h under Ar atmosphere. Then, after cooling to r.t., the residue was diluted with toluene and then washed with 20% NaOH aq. (200 ml) and water. The organic phase was concentrated under reduced pressure. The residue was rinsed with chloroform and hexane to afford compound **6** (58 mg, 51%). mp: >300 °C; ^{1}H NMR

(500 MHz, $CDCl_3$): δ 8.24 (s, 4H), 8.14 (s, 2H), 7.92–7.90 (m, 4H), 7.83 (s, 4H), 7.82 (s, 4H), 7.38–7.36 (m, 4H), 7.04–7.03 (m, 4H), 5.31–5.30 (m, 4H); ^{13}C NMR (126 MHz, $CDCl_3$): δ 140.90, 140.44, 137.58, 131.61, 130.51, 130.48, 127.96, 125.58, 125.12, 124.88, 120.94, 120.92, 49.80; IR (KBr): 3442 cm^{-1} (br m), 3050 cm^{-1} (s), 3003 cm^{-1} (s), 2957 cm^{-1} (s), 2927 cm^{-1} (s), 2855 cm^{-1} (s), 1936 cm^{-1} (w), 1792 cm^{-1} (w), 1646 cm^{-1} (m), 1599 cm^{-1} (m), 1545 cm^{-1} (m), 1432 cm^{-1} (s), 1324 cm^{-1} (s), 1287 cm^{-1} (m), 1165 cm^{-1} (s), 1007 cm^{-1} (m), 951 cm^{-1} (m), 897 cm^{-1} (s), 854 cm^{-1} (m), 769 cm^{-1} (s), 740 cm^{-1} (s), 688 cm^{-1} (s); HRMS (m/z): $[M]^+$ calcd. for $C_{50}H_{30}$, 630.2342; found, 630.2342.

3. X-ray single crystal structure

Compound 5

CCDC	2096408
Empirical formula	$C_{46}H_{30}N_4$
Formula weight	638.74
Temperature	90 K
Wavelength	0.71073 Å
Crystal system	Hexagonal
Space group	$P6_3/m$ (No. 176)
Unit cell dimensions	a = 24.433(9) Å
	c = 11.164(5) Å
Volume	5771(5) $Å^3$
Z	6
Density (calculated)	1.103 Mg/m^3
Absorption coefficient	0.065 mm^{-1}
F(000)	2004
Crystal size	0.200 × 0.050 × 0.050 mm^3
Theta range for data collection	0.962 to 23.500°
Index ranges	$-27 \le h \le 27$, $-27 \le k \le 20$, $-12 \le l \le 12$
Reflections collected	27480
Independent reflections	3017 [R(int) = 0.1748]
Completeness to theta = 23.500°	99.8%
Absorption correction	Semi-empirical from equivalents
Max. and min. transmission	0.997 and 0.854
Refinement method	Full-matrix least-squares on F^2
Data / restraints / parameters	3017 / 0 / 226
Goodness-of-fit on F^2	1.028
Final R indices [I > 2sigma(I)]	R_1 = 0.0952, wR_2 = 0.2168
R indices (all data)	R_1 = 0.2050, wR_2 = 0.2657
Extinction coefficient	n/a
Largest diff. peak and hole	0.303 and –0.246 e.$Å^{-3}$

* The contributions to the scattering arising from the presence of the disordered solvent molecules in the crystal were removed by use of the utility SQUEEZE program in the PLATON software package.[27]

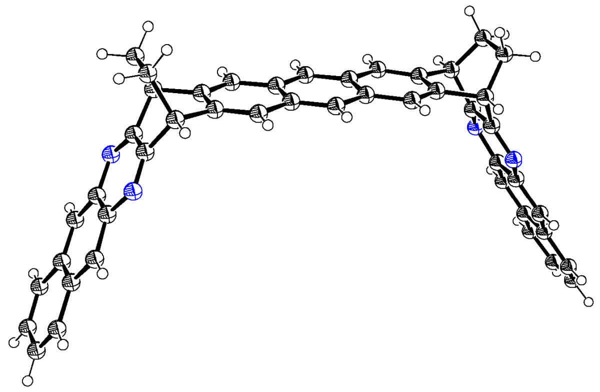

Supplementary Figure 14. Single-crystal X-ray structure of **compound 5**. Thermal ellipsoids represent 50% probability.

Compound 6

CCDC	2096406
Empirical formula	$C_{58}H_{30}$
Formula weight	726.82
Temperature	133 K
Wavelength	0.71073 Å
Crystal system	Hexagonal
Space group	$P6_3/m$
Unit cell dimensions	a = 24.455(4) Å
	c = 11.3292(19) Å
Volume	5868(2) Å^3
Z	6
Density (calculated)	1.234 Mg/m^3
Absorption coefficient	0.070 mm^{-1}
F(000)	2268
Crystal size	0.300 × 0.050 × 0.050 mm^3
Theta range for data collection	1.923 to 25.000°
Index ranges	$-24 \leq h \leq 29$, $-28 \leq k \leq 29$, $-13 \leq l \leq 13$
Reflections collected	31121
Independent reflections	3630 [R(int) = 0.1066]
Completeness to theta = 23.500°	99.7%
Absorption correction	Semi-empirical from equivalents
Max. and min. transmission	0.997 and 0.919
Refinement method	Full-matrix least-squares on F^2
Data / restraints / parameters	3630 / 0 / 265
Goodness-of-fit on F^2	1.037
Final R indices [I > 2sigma(I)]	R_1 = 0.0807, wR_2 = 0.2080
R indices (all data)	R_1 = 0.1583, wR_2 = 0.2853
Extinction coefficient	n/a
Largest diff. peak and hole	0.502 and –0.304 e.Å^{-3}

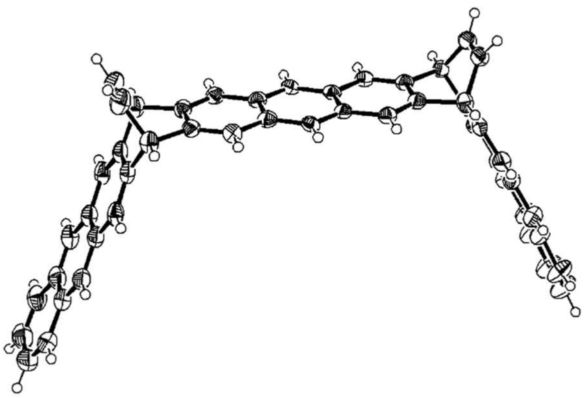

Supplementary Figure 15. Single-crystal X-ray structure of **compound 6**. Thermal ellipsoids represent 50% probability. Solvent molecules are omitted for clarity.

4. NMR spectra

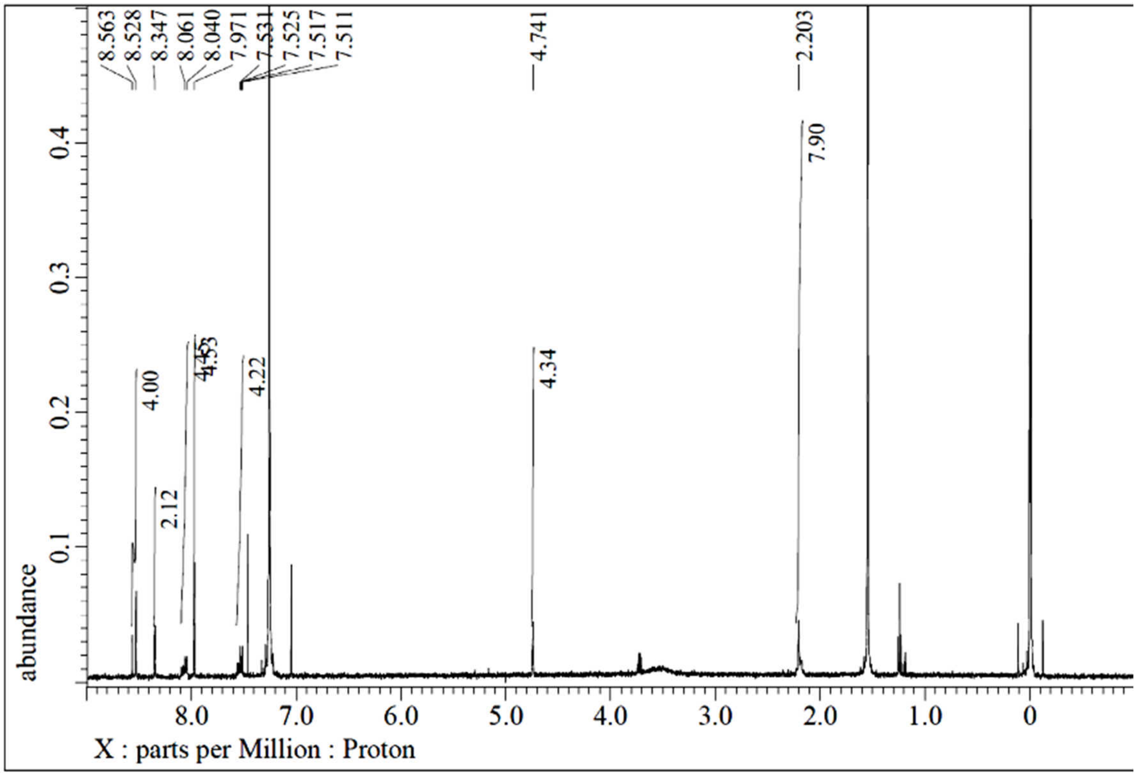

Supplementary Figure 16. ^{1}H NMR spectrum of **compound 5** in $CDCl_3$ at r.t..

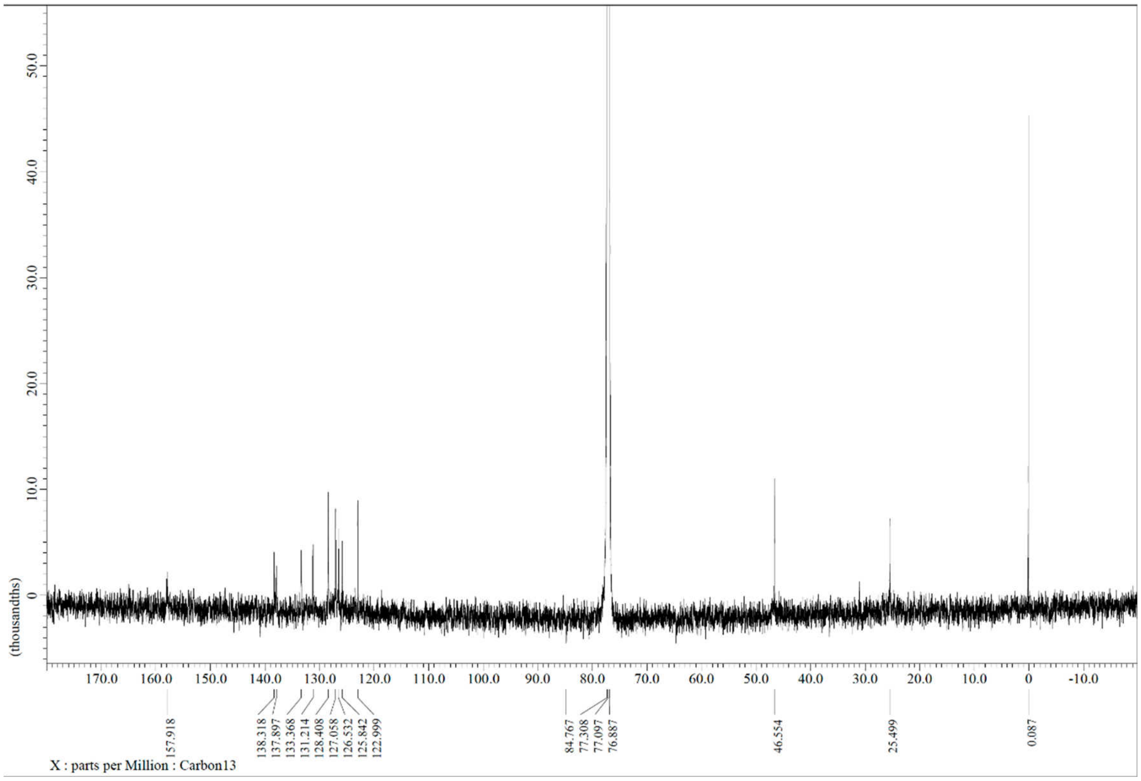

Supplementary Figure 17. ^{13}C NMR spectrum of **compound 5** in $CDCl_3$ at r.t..

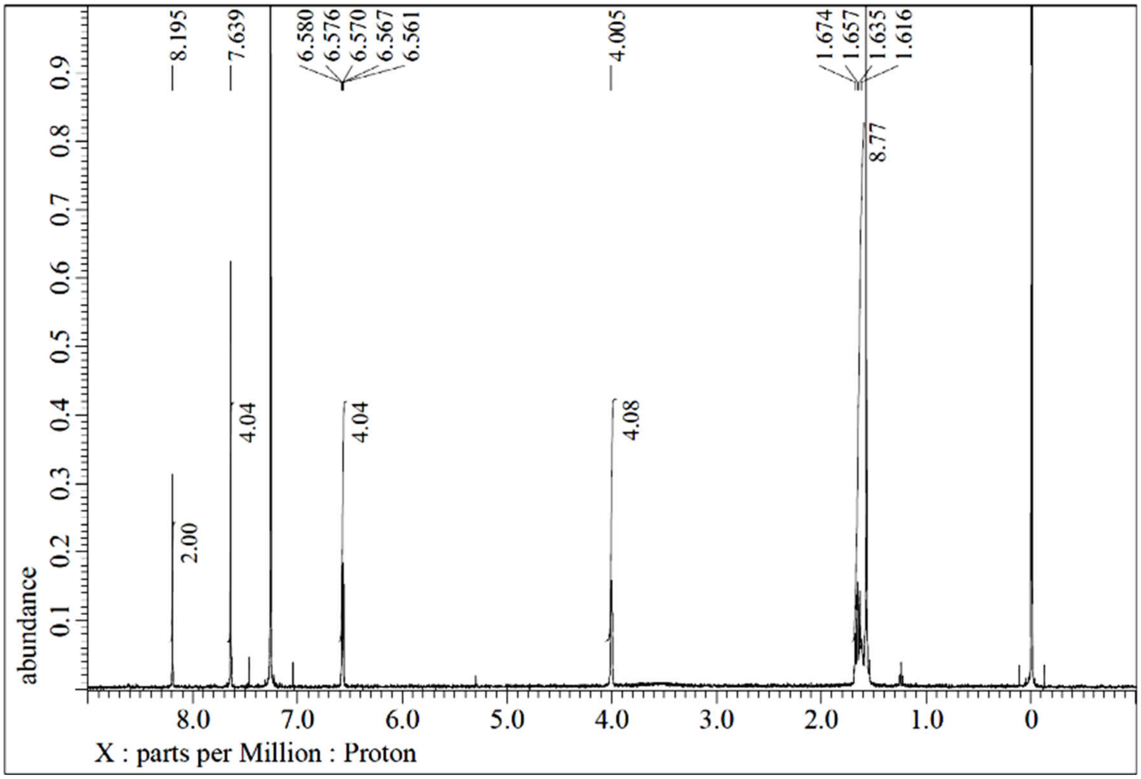

Supplementary Figure 18. ^{1}H NMR spectrum of **compound ii** in $CDCl_3$ at r.t..

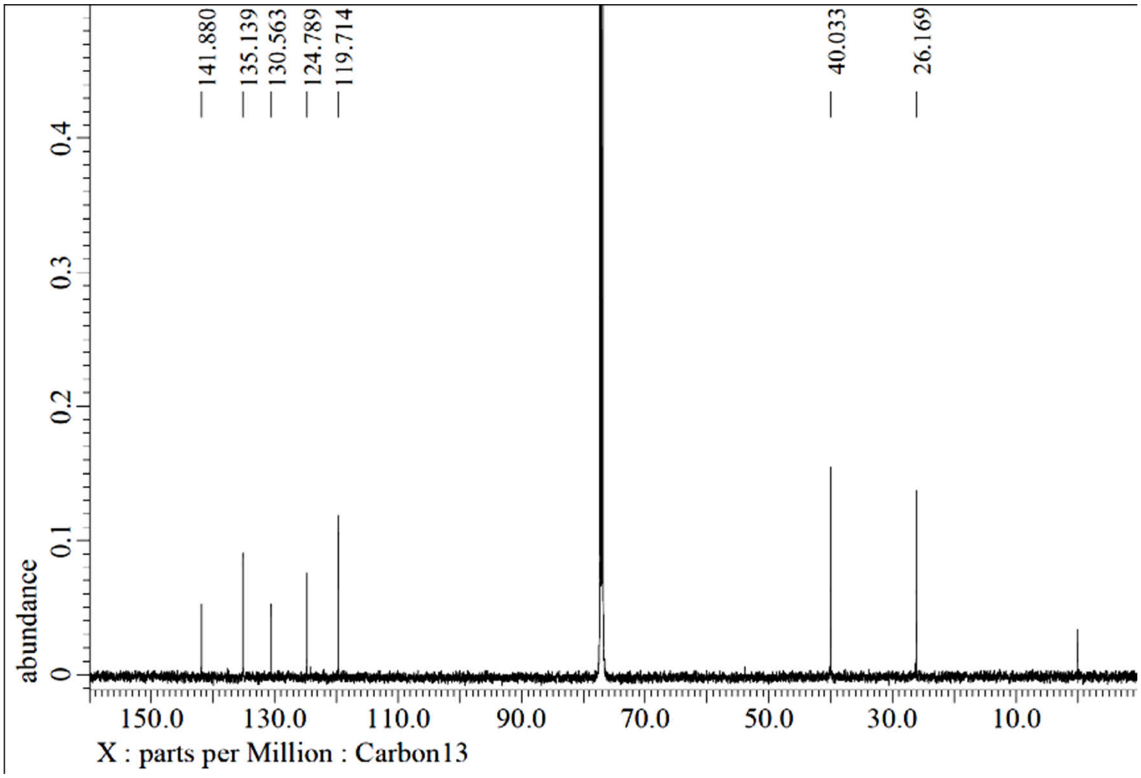

Supplementary Figure 19. ^{13}C NMR spectrum of **compound ii** in $CDCl_3$ at r.t..

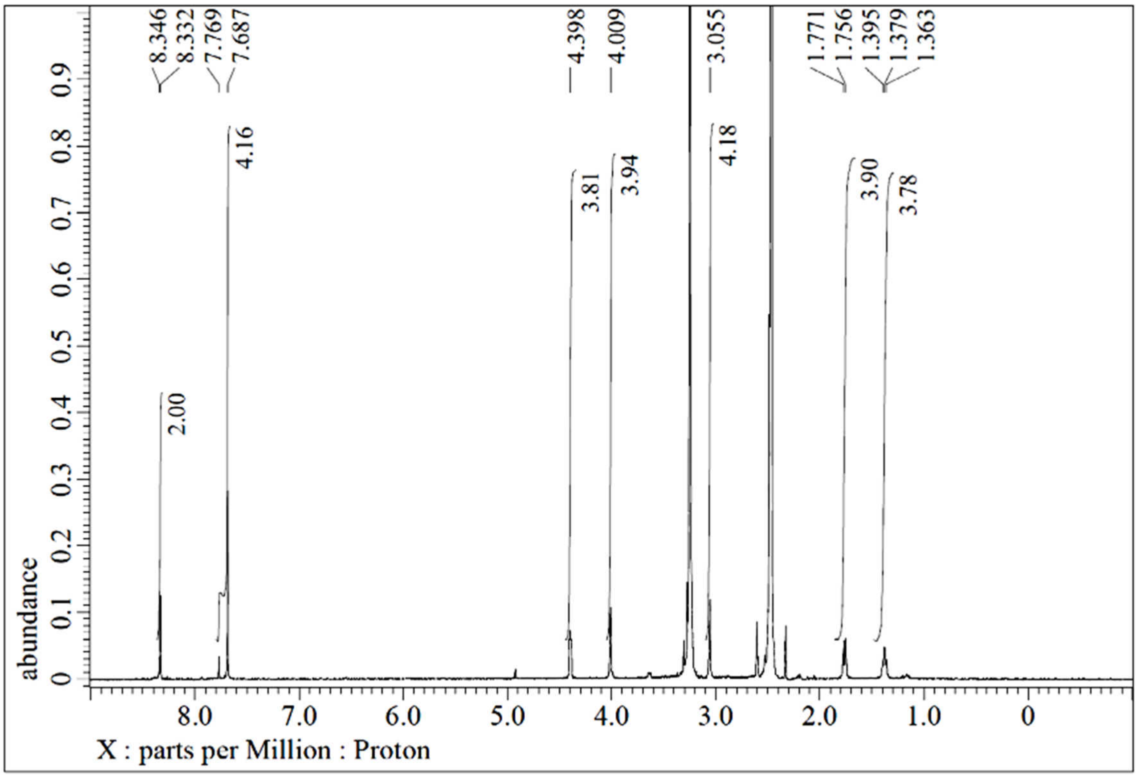

Supplementary Figure 20. ^{1}H NMR spectrum of **compound iii** in $CDCl_3$ at r.t..

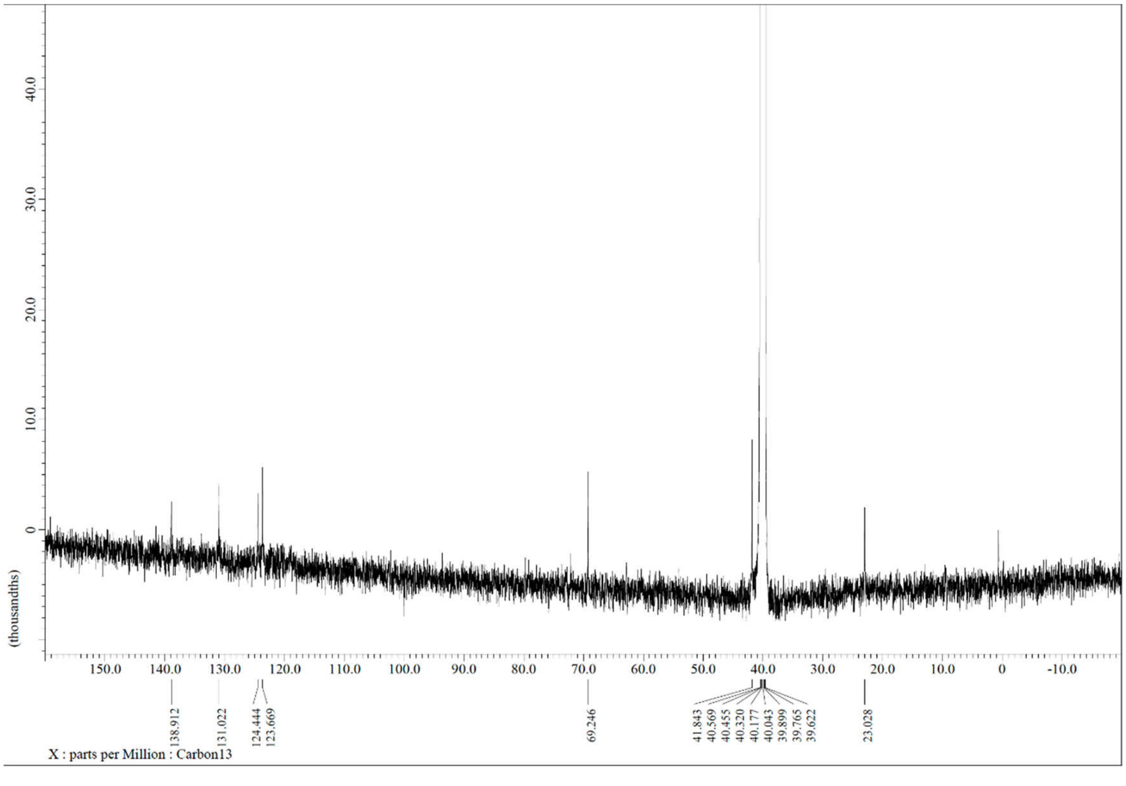

Supplementary Figure 21. ^{13}C NMR spectrum of **compound iii** in $CDCl_3$ at r.t..

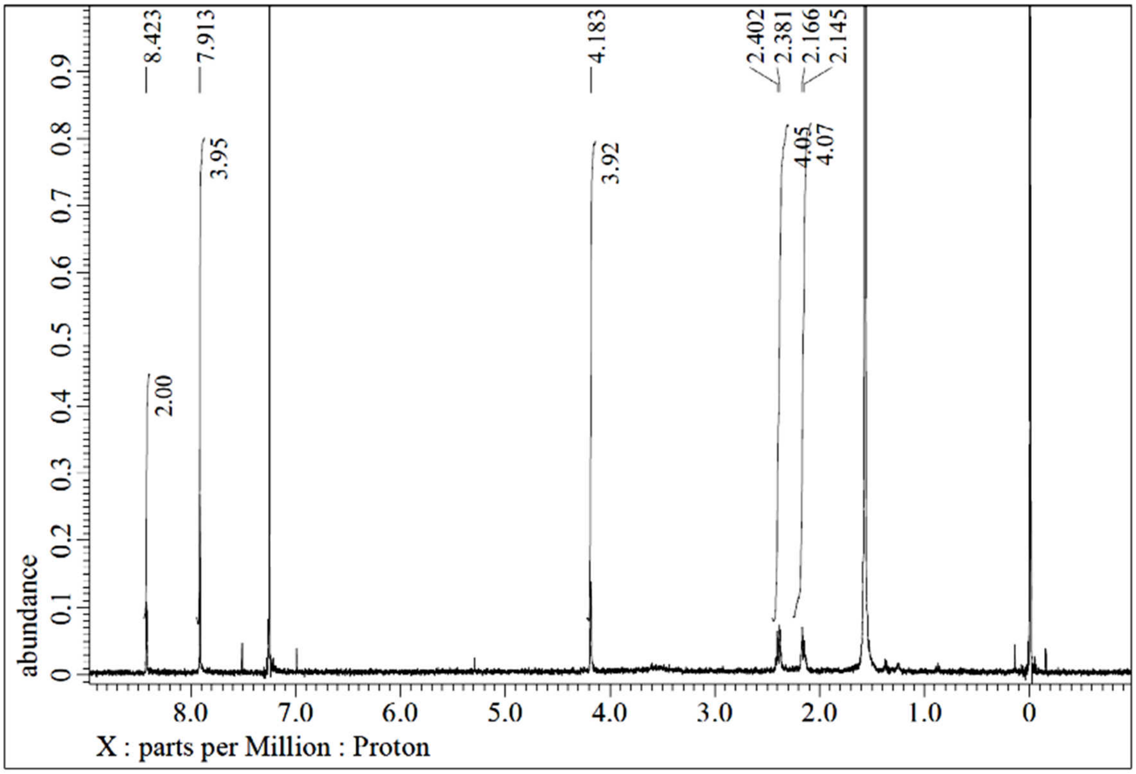

Supplementary Figure 22. ^{1}H NMR spectrum of **compound iv** in $CDCl_3$ at r.t..

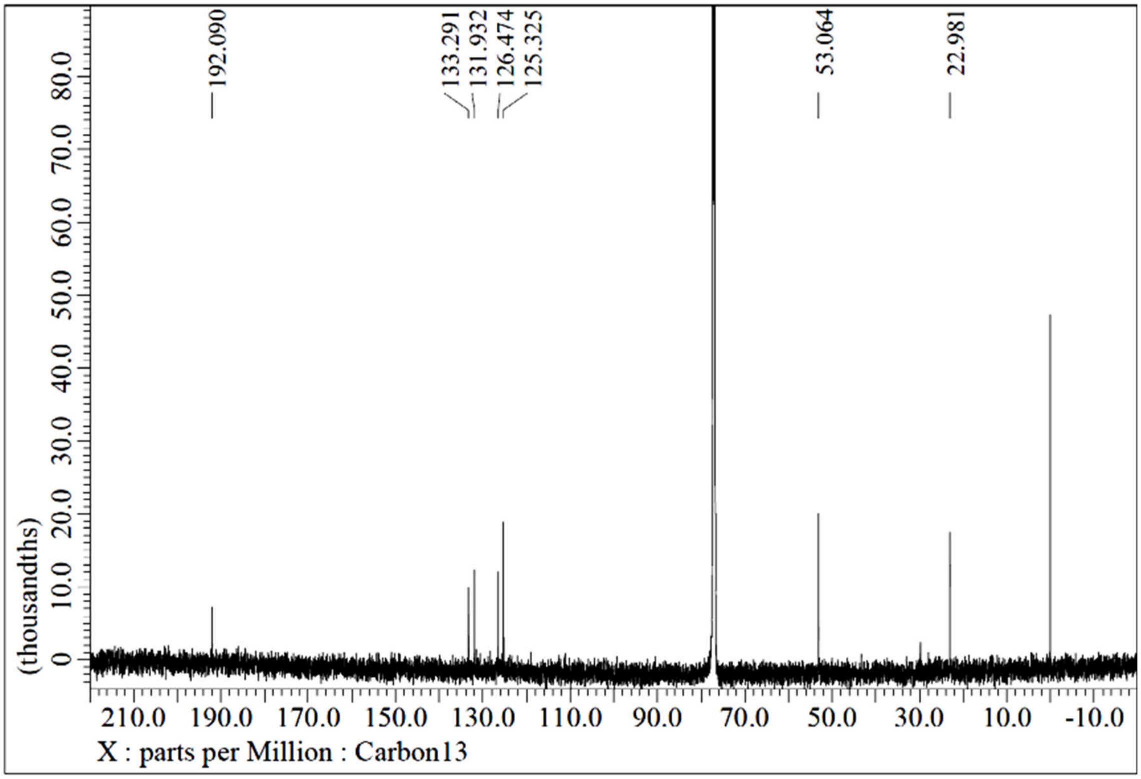

Supplementary Figure 23. ^{13}C NMR spectrum of **compound iv** in $CDCl_3$ at r.t..

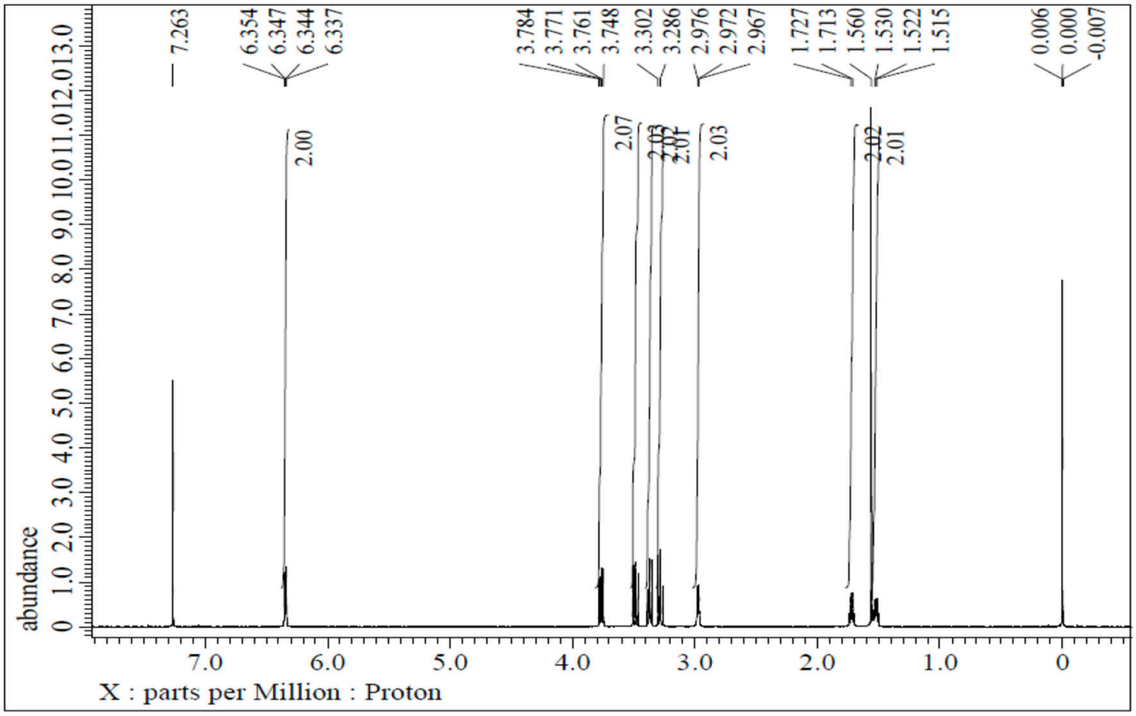

Supplementary Figure 24. ^{1}H NMR spectrum of **compound va** in $CDCl_3$ at r.t..

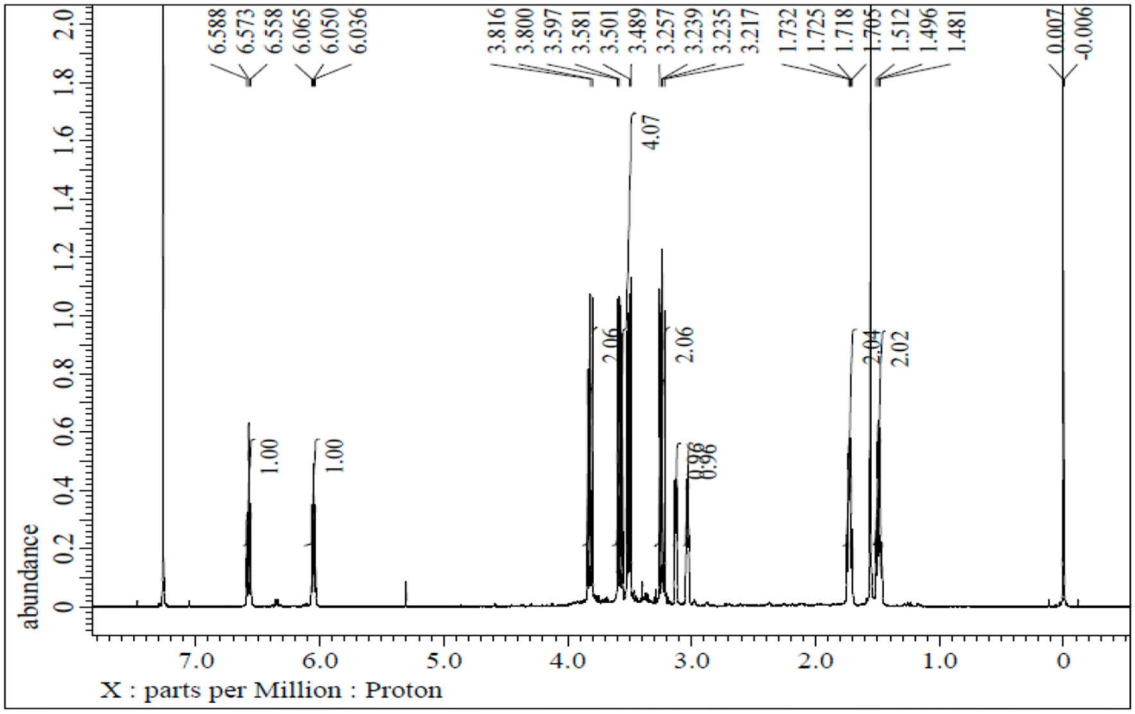

Supplementary Figure 25. ^{1}H NMR spectrum of **compound vb** in $CDCl_3$ at r.t..

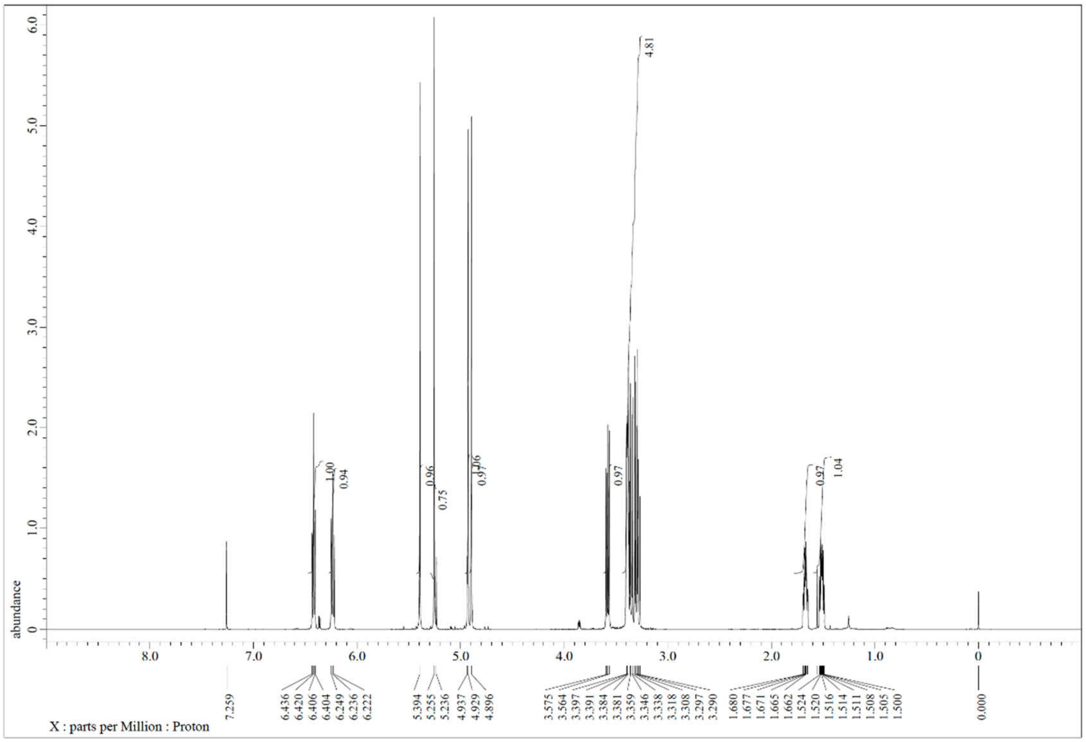

Supplementary Figure 26. ^{1}H NMR spectrum of **compound vi** in $CDCl_3$ at r.t..

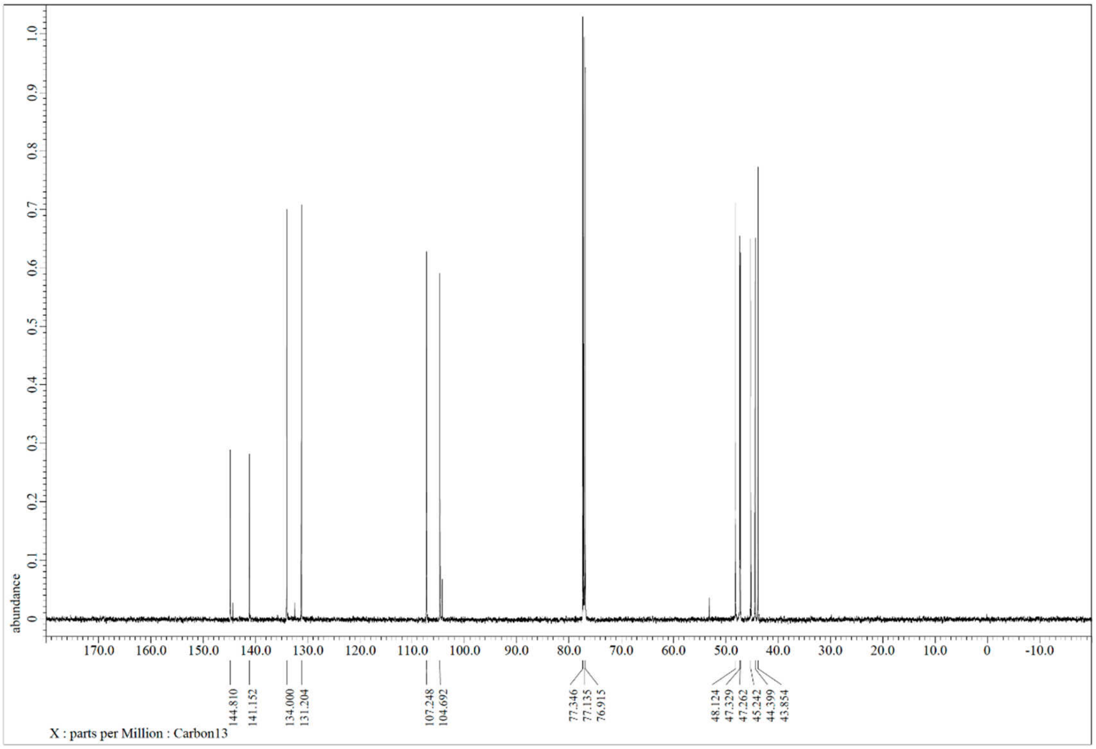

Supplementary Figure 27. ^{13}C NMR spectrum of **compound vi** in $CDCl_3$ at r.t..

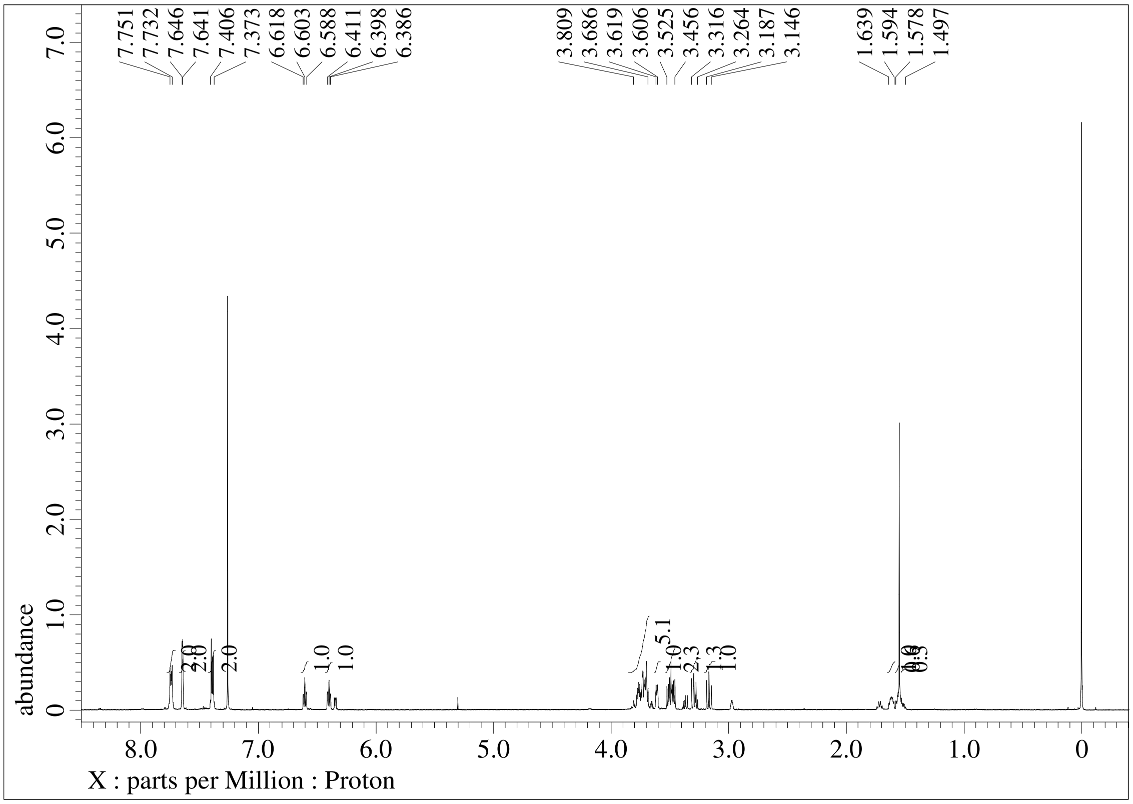

Supplementary Figure 28. ^{1}H NMR spectrum of **compound vii** in $CDCl_3$ at r.t..

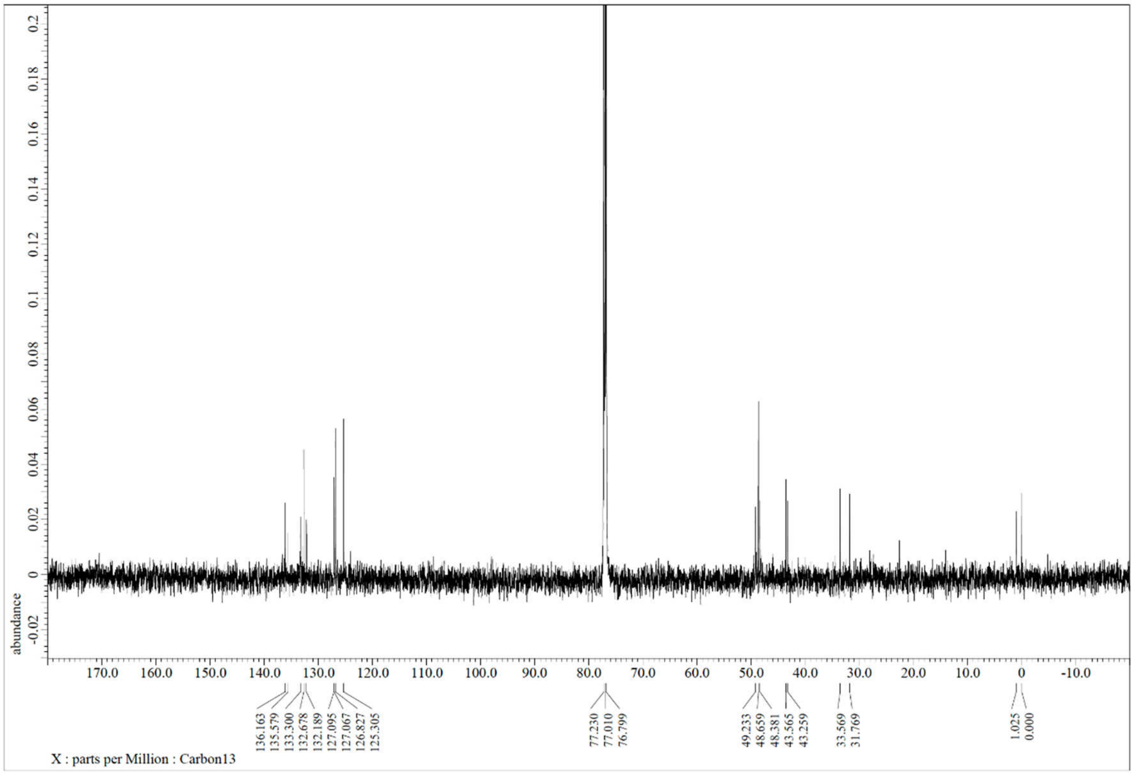

Supplementary Figure 29. ^{13}C NMR spectrum of **compound vii** in $CDCl_3$ at r.t..

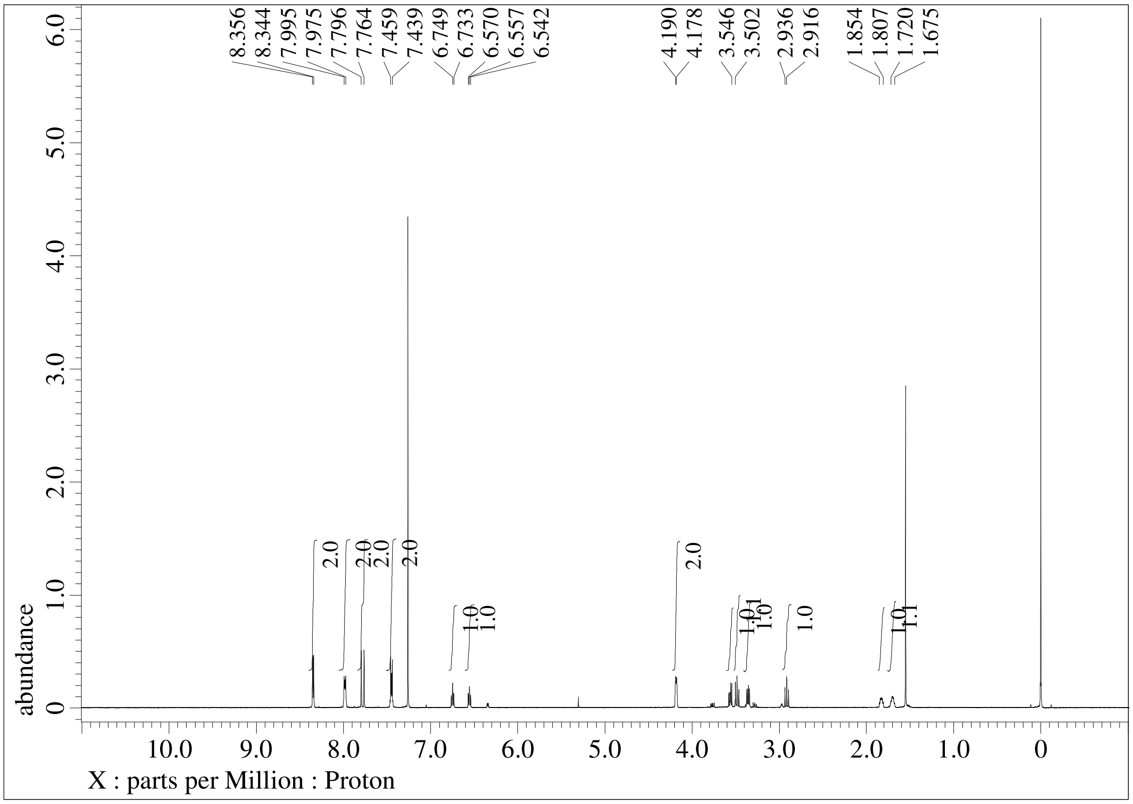

Supplementary Figure 30. ^{1}H NMR spectrum of **compound viii** in $CDCl_3$ at r.t..

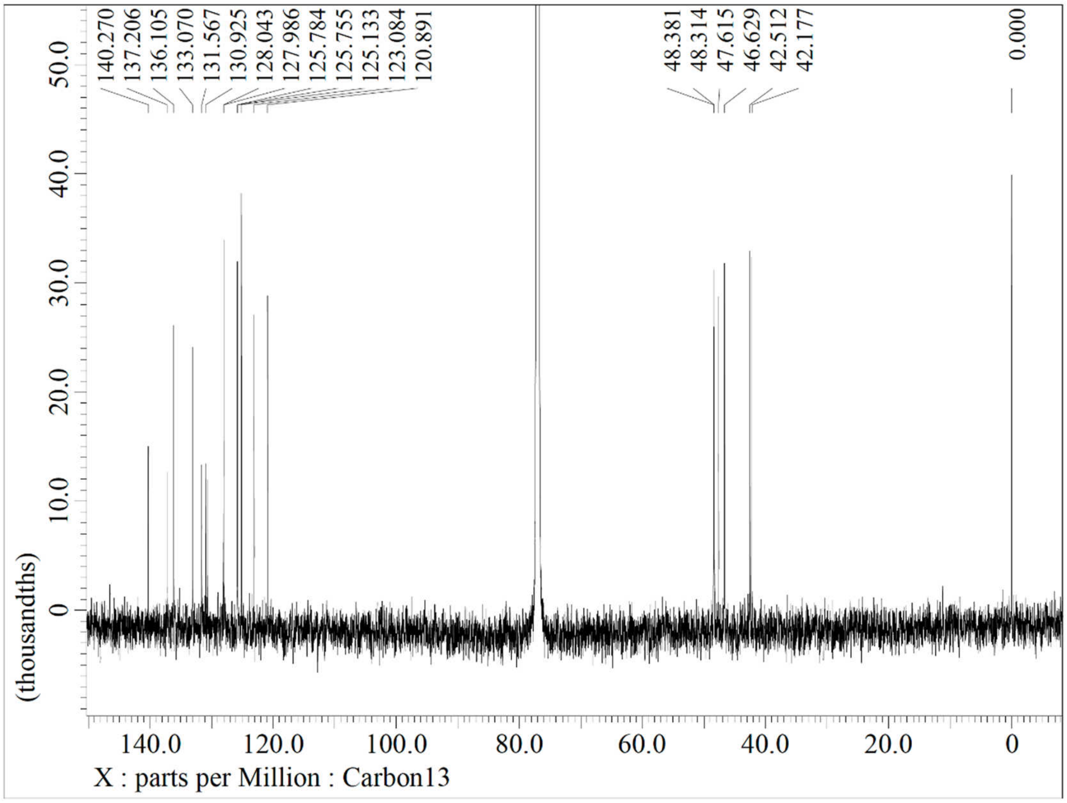

Supplementary Figure 31. ^{13}C NMR spectrum of **compound viii** in $CDCl_3$ at r.t..

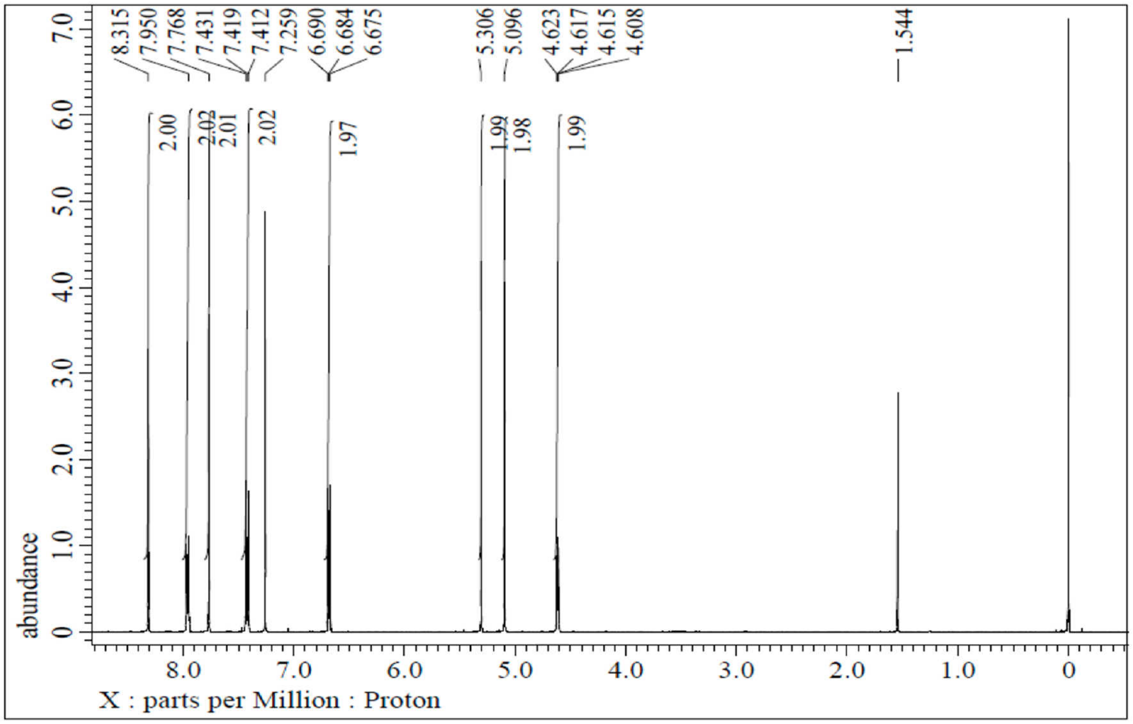

Supplementary Figure 32. ^{1}H NMR spectrum of **compound ix** in $CDCl_3$ at r.t..

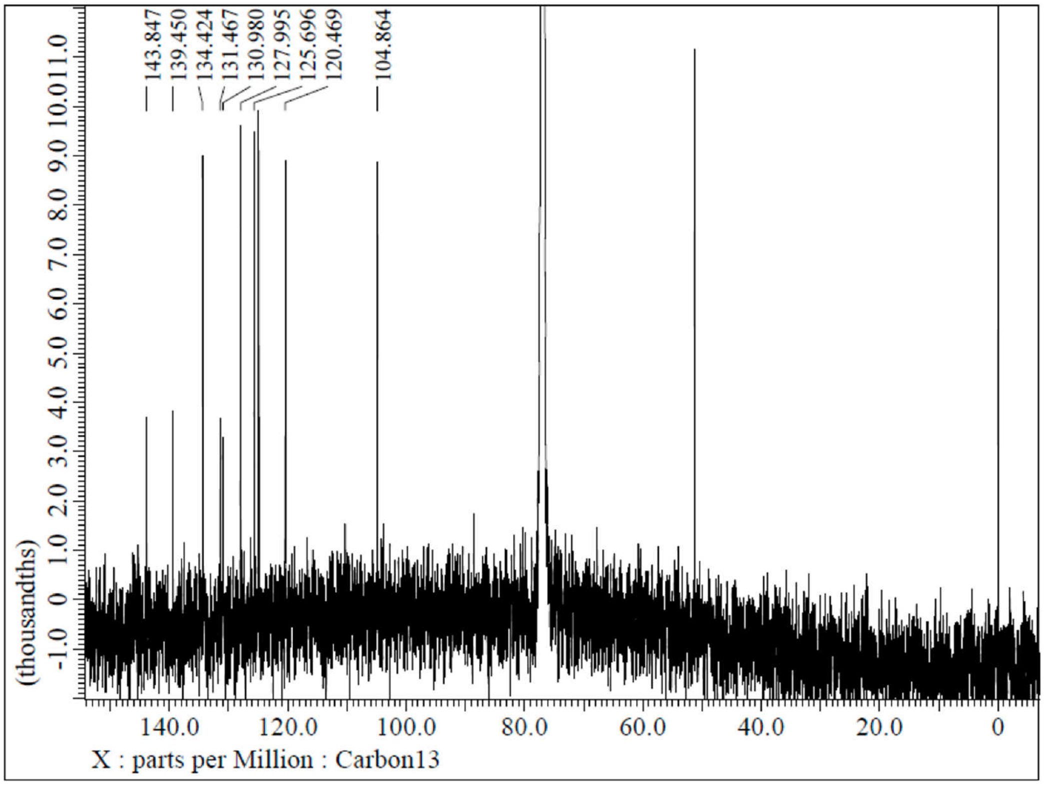

Supplementary Figure 33. ^{13}C NMR spectrum of **compound ix** in $CDCl_3$ at r.t..

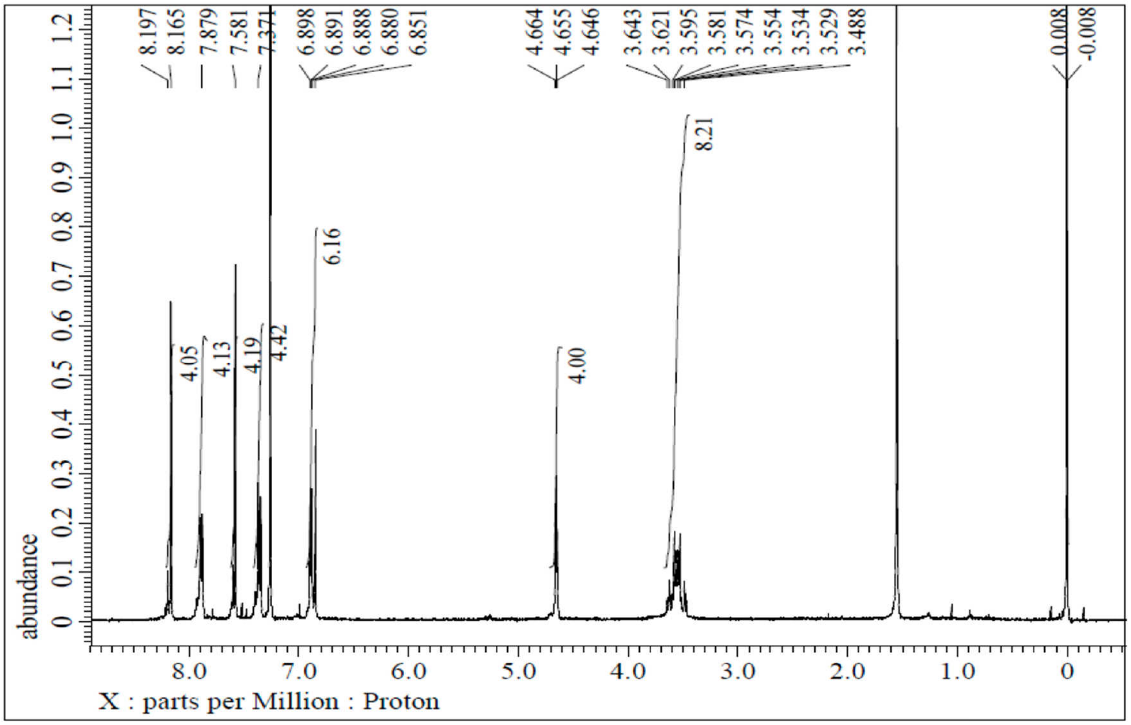

Supplementary Figure 34. ^{1}H NMR spectrum of **compound x** in $CDCl_3$ at r.t..

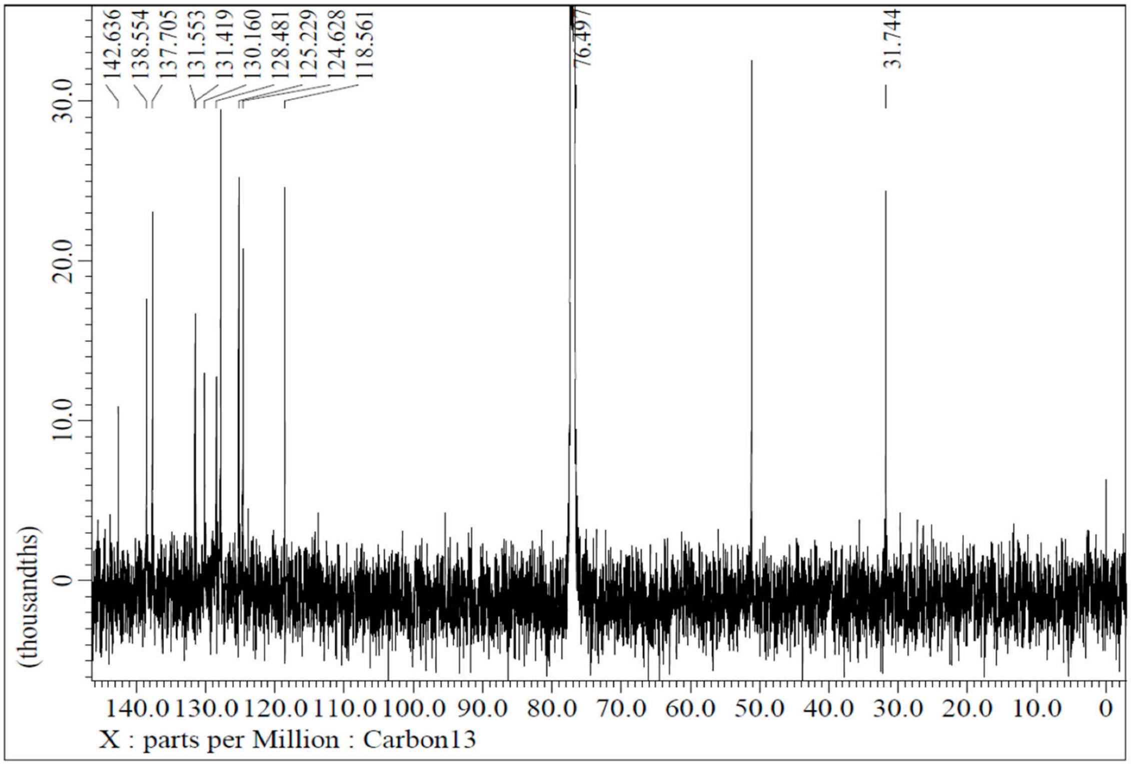

Supplementary Figure 35. ^{13}C NMR spectrum of **compound x** in $CDCl_3$ at r.t..

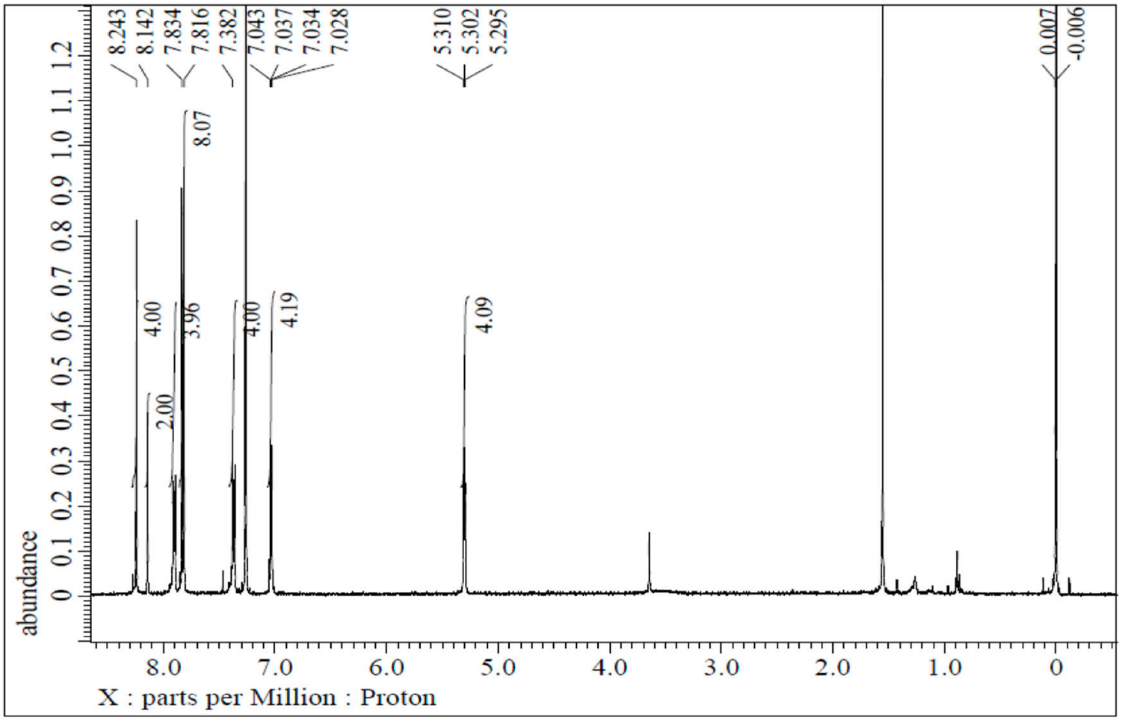

Supplementary Figure 36. ^{1}H NMR spectrum of **compound 6** in $CDCl_3$ at r.t..

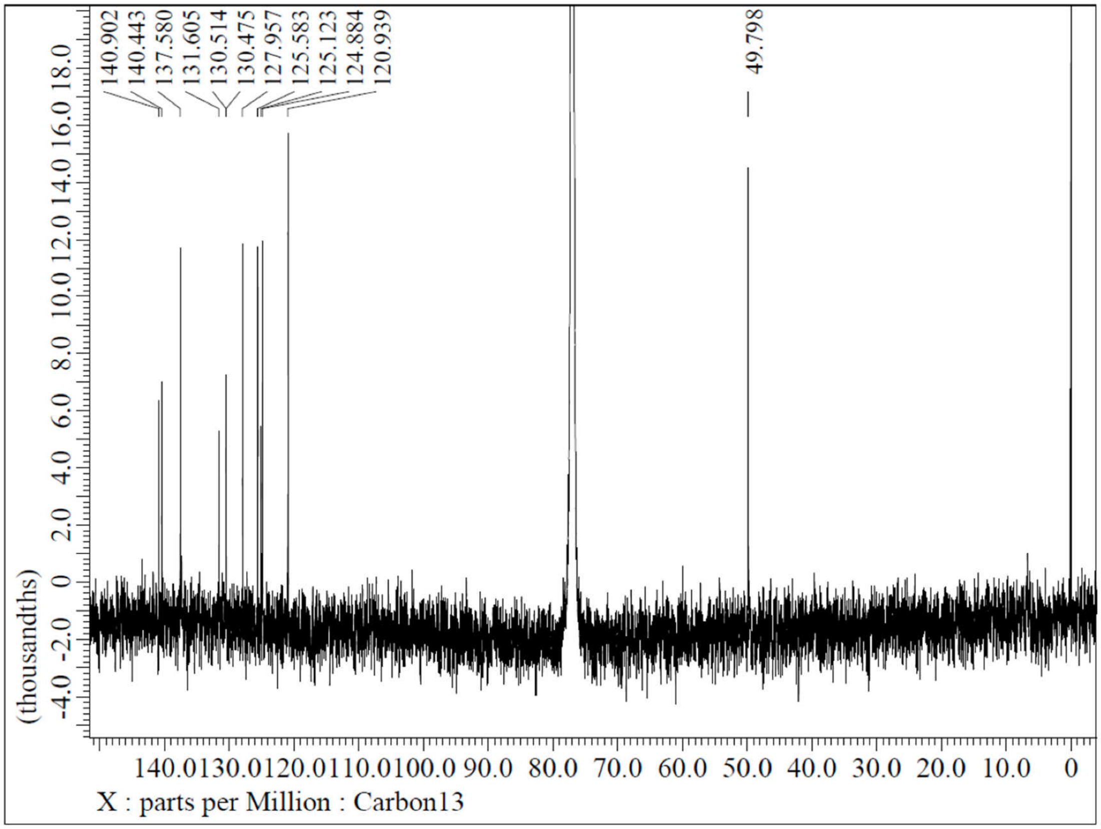

Supplementary Figure 37. ^{13}C NMR spectrum of **compound 6** in $CDCl_3$ at r.t..

5. Mass spectra

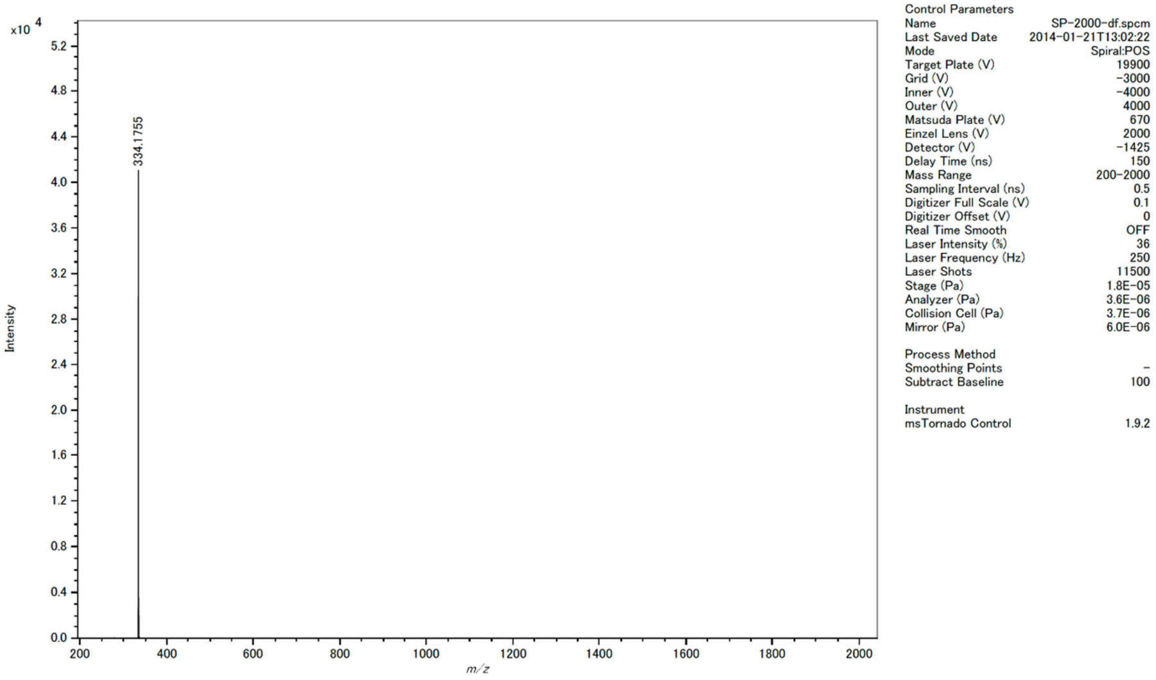

Supplementary Figure 38. MS spectrum of **compound ii**.

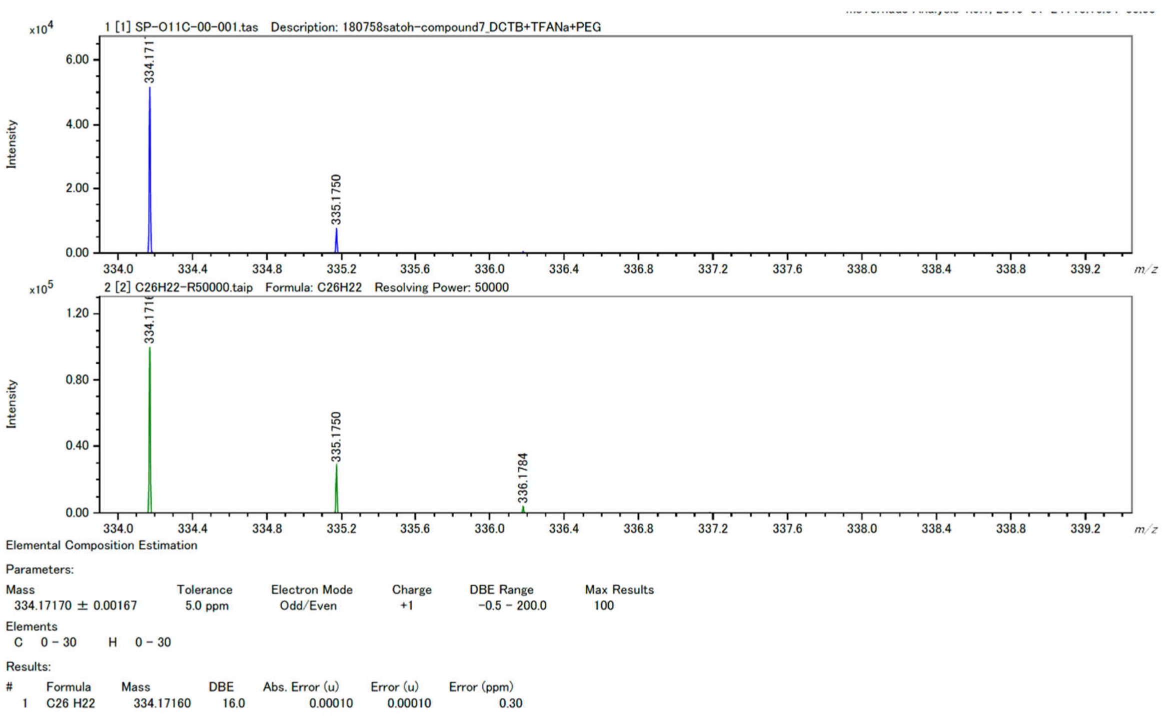

Supplementary Figure 39. HRMS spectrum of **compound ii**.

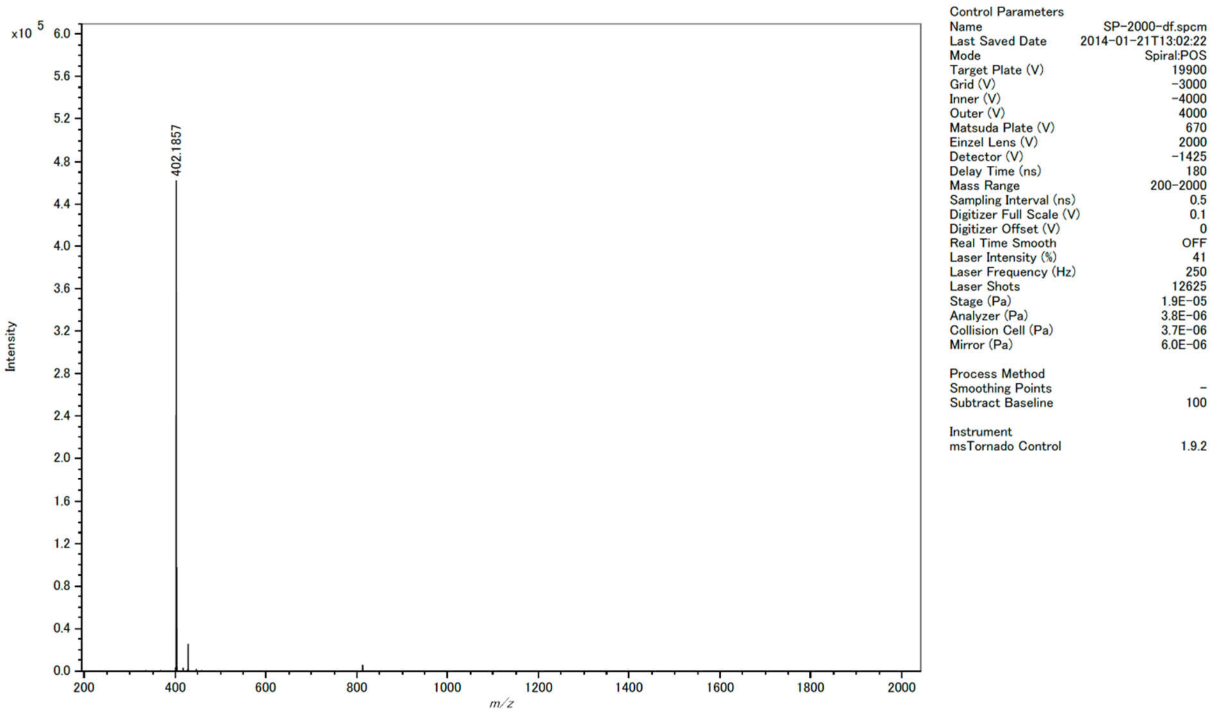

Supplementary Figure 40. MS spectrum of **compound iii**.

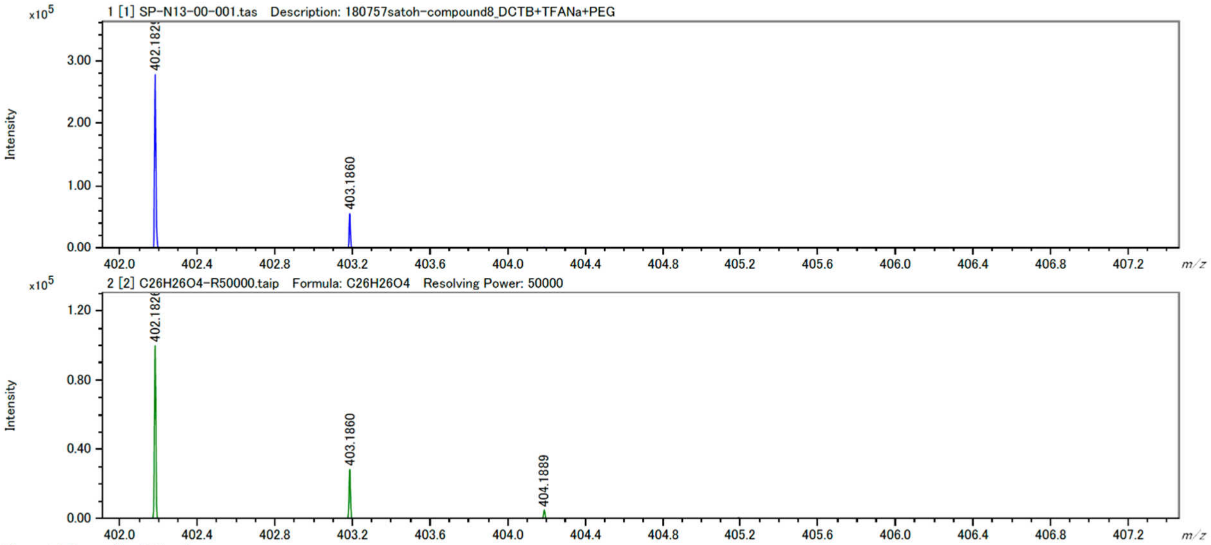

Elemental Composition Estimation

Parameters:

Mass	Tolerance	Electron Mode	Charge	DBE Range	Max Results
402.18287 ± 0.00201	5.0 ppm	Odd/Even	+1	-0.5 - 200.0	100

Elements

C 0 - 30 H 0 - 30 O 0 - 7

Results:

#	Formula	Mass	DBE	Abs. Error (u)	Error (u)	Error (ppm)
1	C26 H26 O4	402.18256	14.0	0.00031	0.00031	0.77

Supplementary Figure 41. HRMS spectrum of **compound iii**.

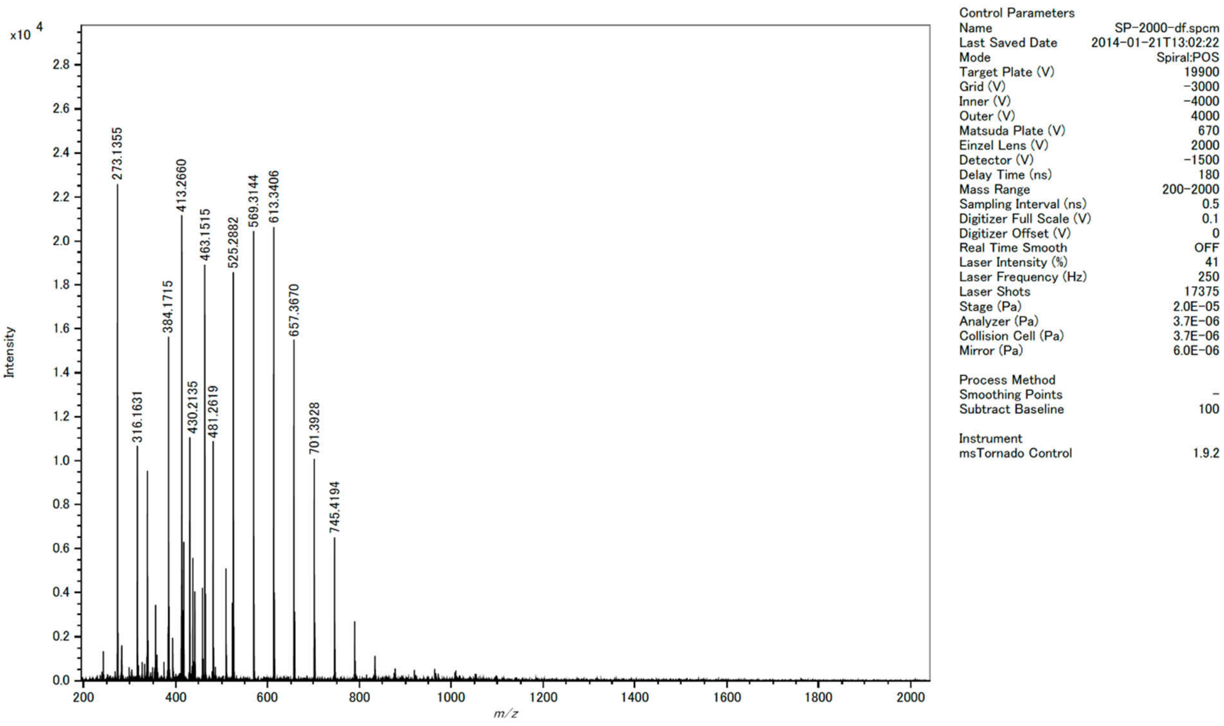

Supplementary Figure 42. MS spectrum of **compound iv**.

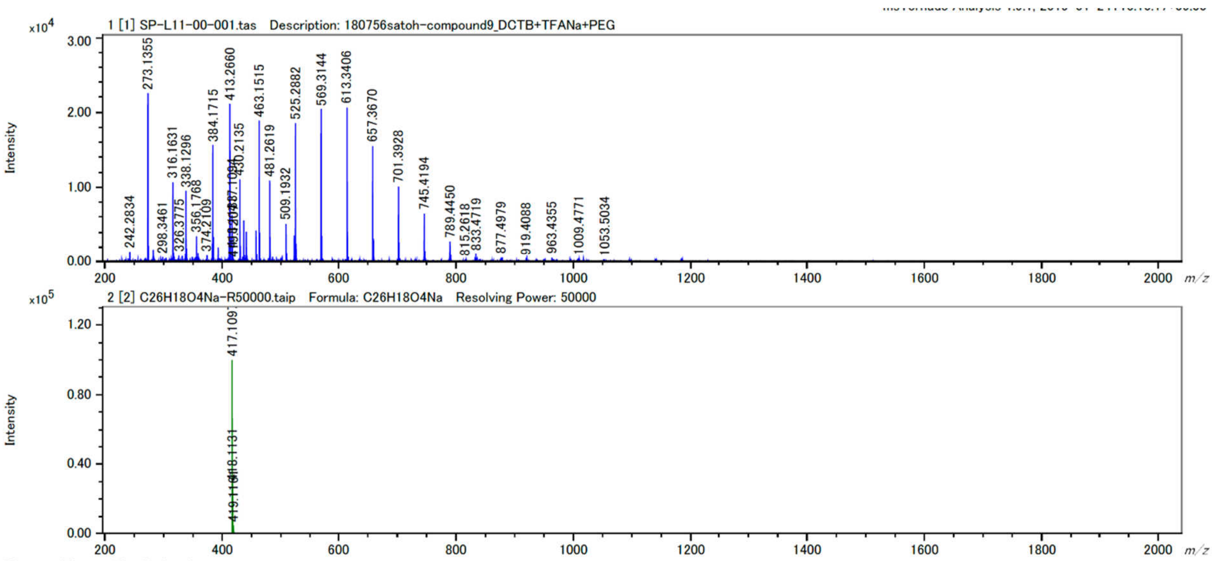

Elemental Composition Estimation

Parameters:

Mass	Tolerance	Electron Mode	Charge	DBE Range	Max Results
417.10945 ± 0.00209	5.0 ppm	Odd/Even	+1	-0.5 – 200.0	100

Elements

C 0 – 30 H 0 – 25 O 0 – 7 Na 0 – 1

Results:

#	Formula	Mass	DBE	Abs. Error (u)	Error (u)	Error (ppm)
1	C26 H18 O4 Na	417.10973	17.5	0.00028	-0.00028	-0.68

Supplementary Figure 43. MS spectrum of **compound iv**.

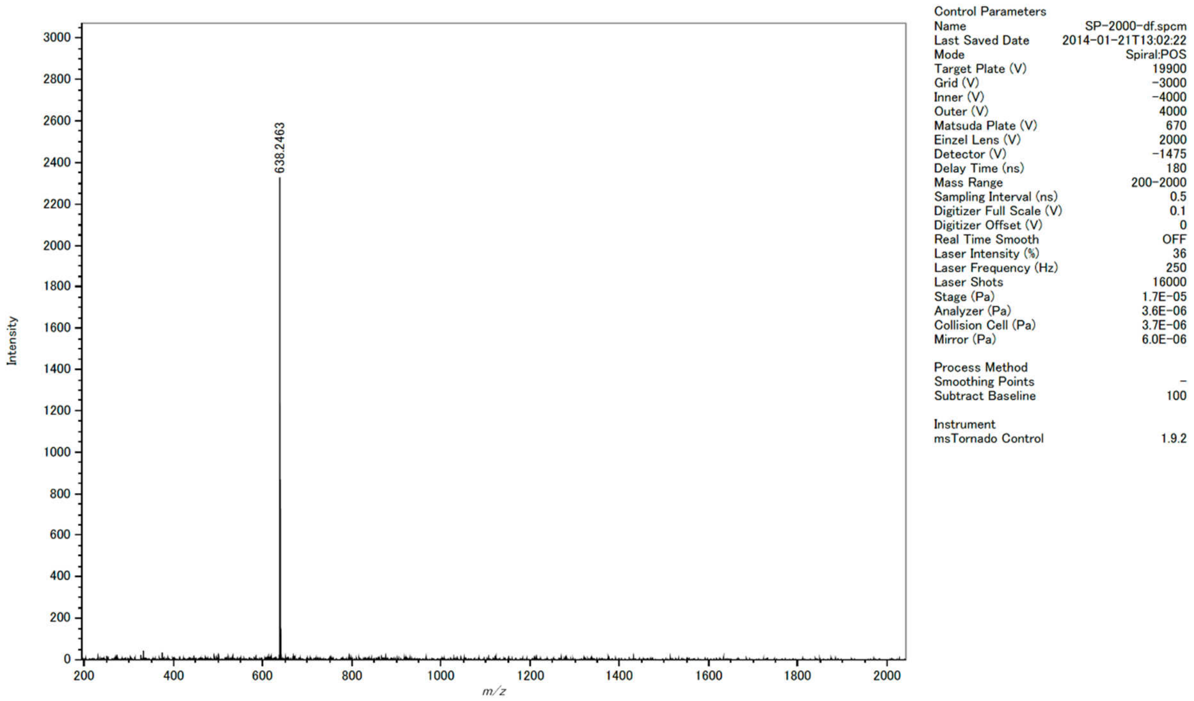

Supplementary Figure 44. MS spectrum of **compound 5**.

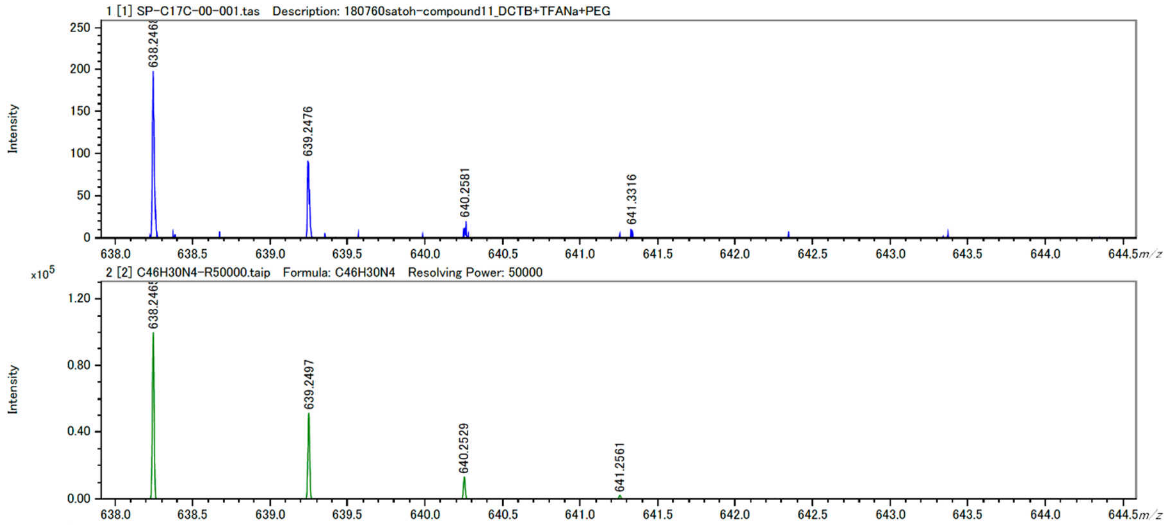

Elemental Composition Estimation

Parameters:

Mass	Tolerance	Electron Mode	Charge	DBE Range	Max Results
638.24678 ± 0.00319	5.0 ppm	Odd/Even	+1	-0.5 - 200.0	100

Elements

C 0 - 50 H 0 - 40 N 0 - 4

Results:

#	Formula	Mass	DBE	Abs. Error (u)	Error (u)	Error (ppm)
1	C46 H30 N4	638.24650	34.0	0.00028	0.00028	0.44

Supplementary Figure 45. HRMS spectrum of **compound 5**.

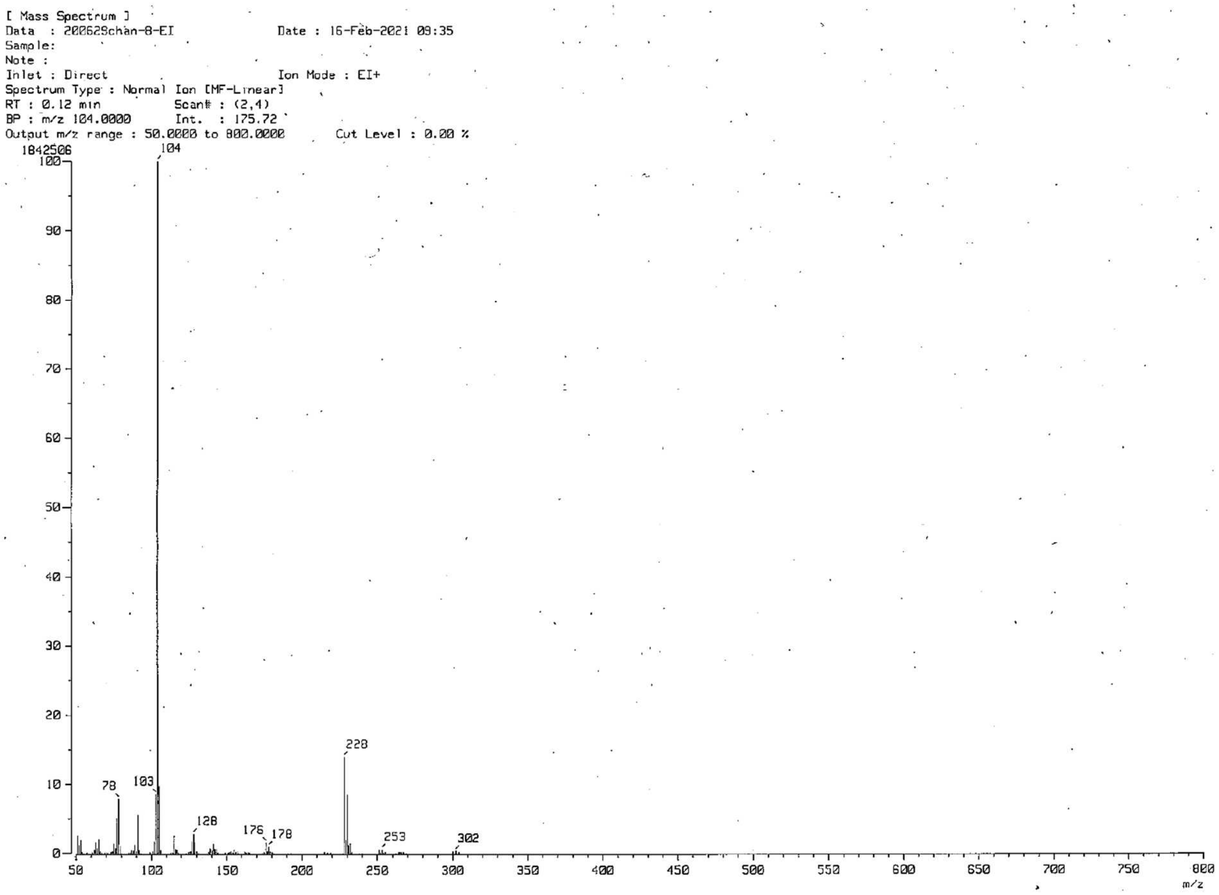

Supplementary Figure 46. MS spectrum of **compound vi**.

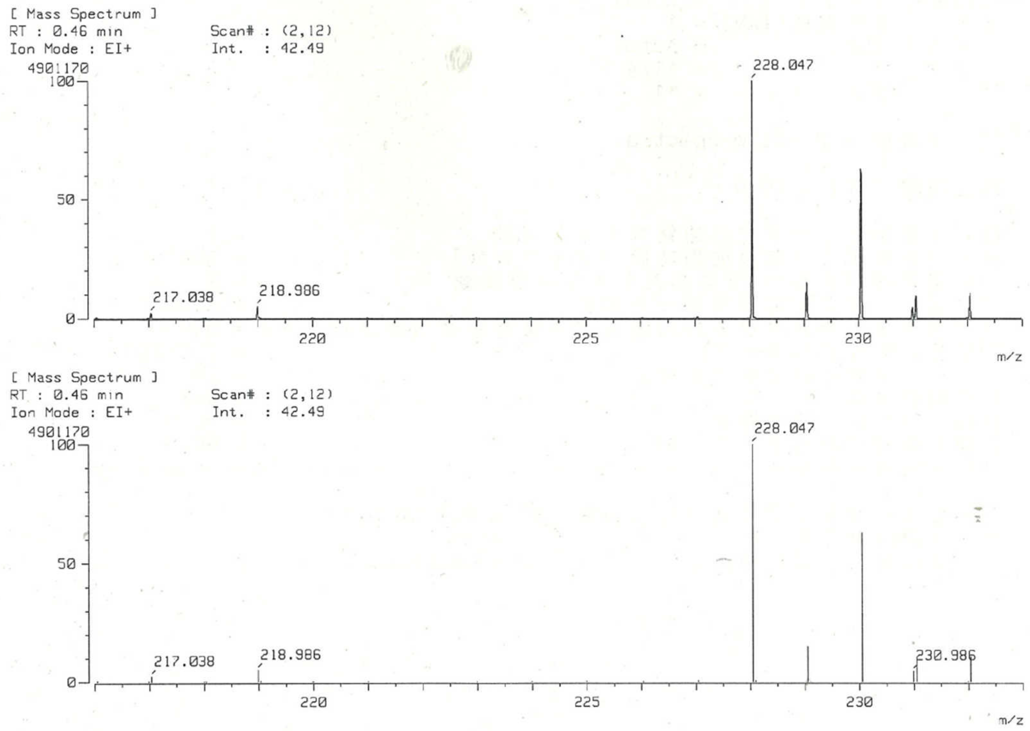

Supplementary Figure 47. HRMS spectrum of **compound vi**.

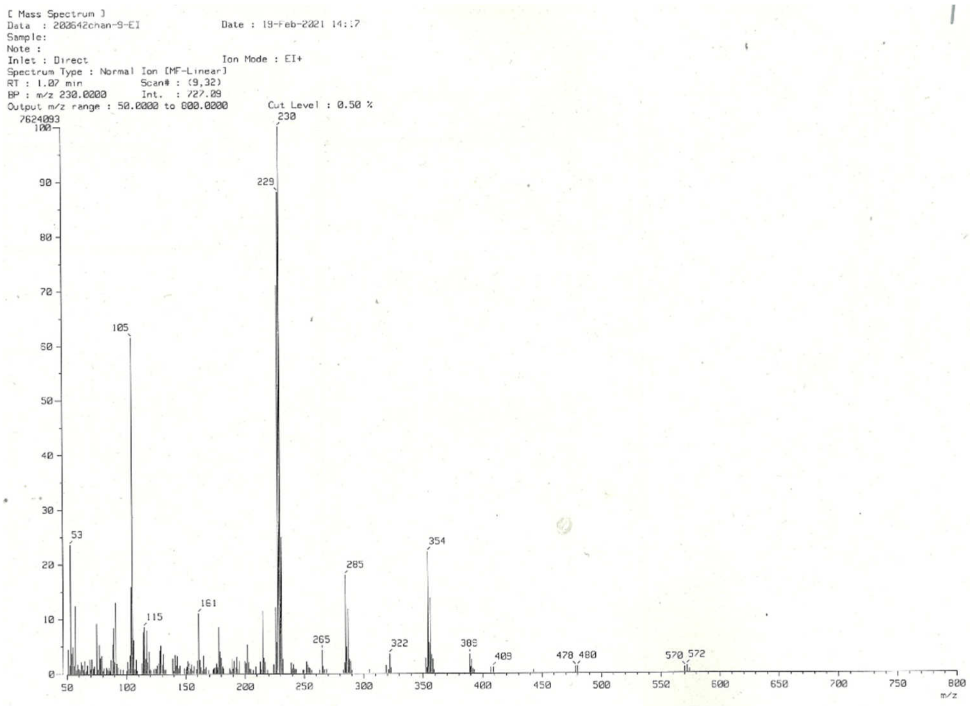

Supplementary Figure 48. MS spectrum of **compound vii**.

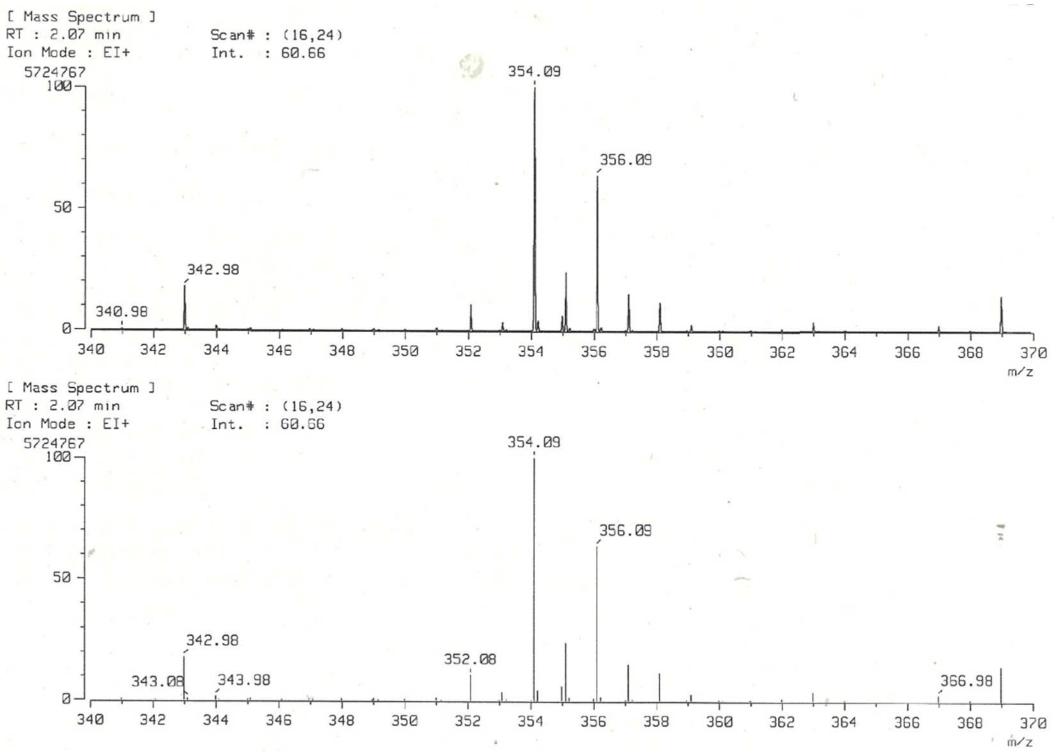

Supplementary Figure 49. HRMS spectrum of **compound vii**.

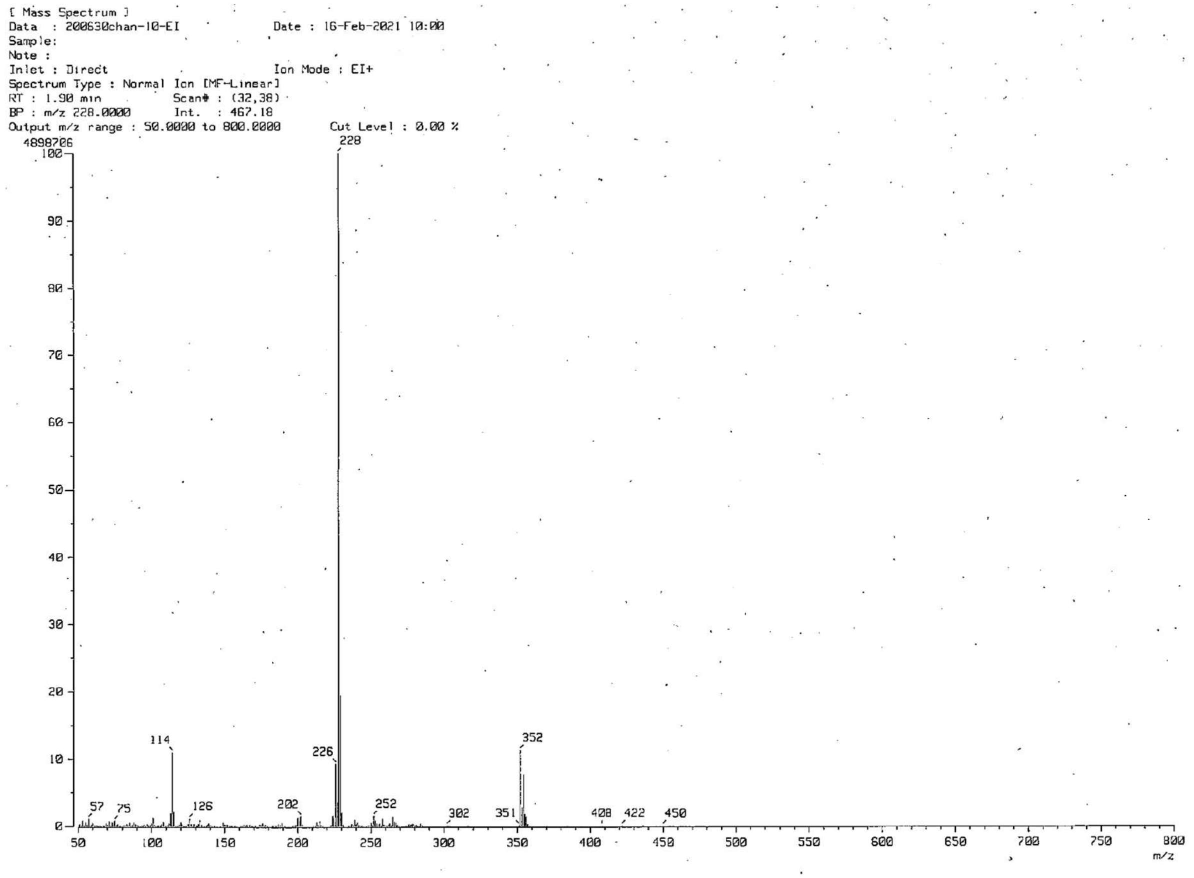

Supplementary Figure 50. MS spectrum of **compound viii**.

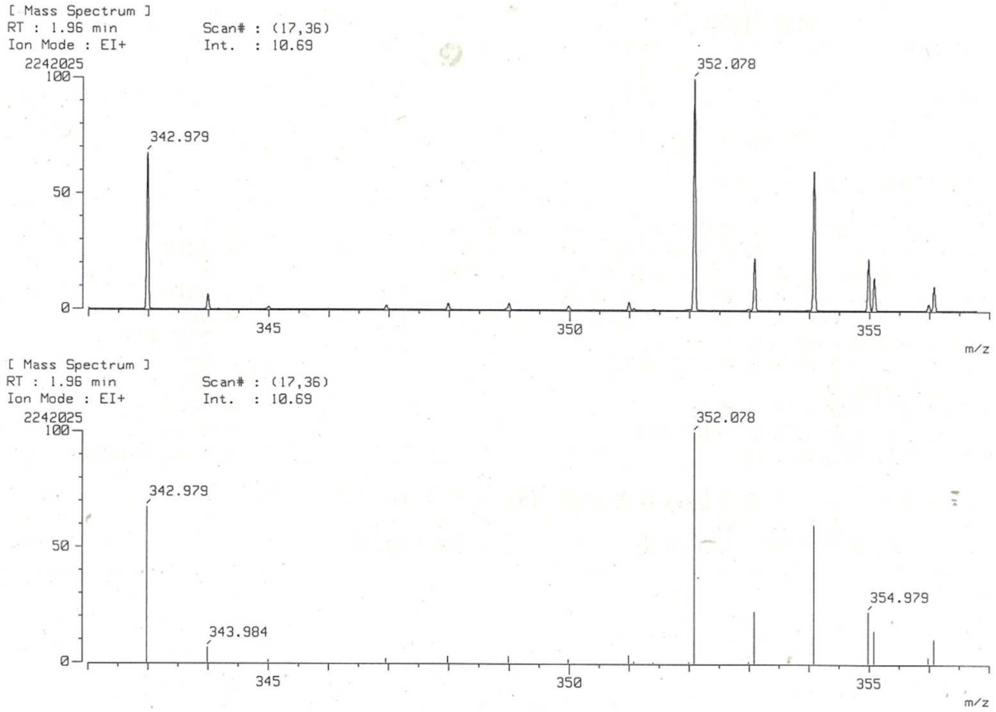

Supplementary Figure 51. HRMS spectrum of **compound viii**.

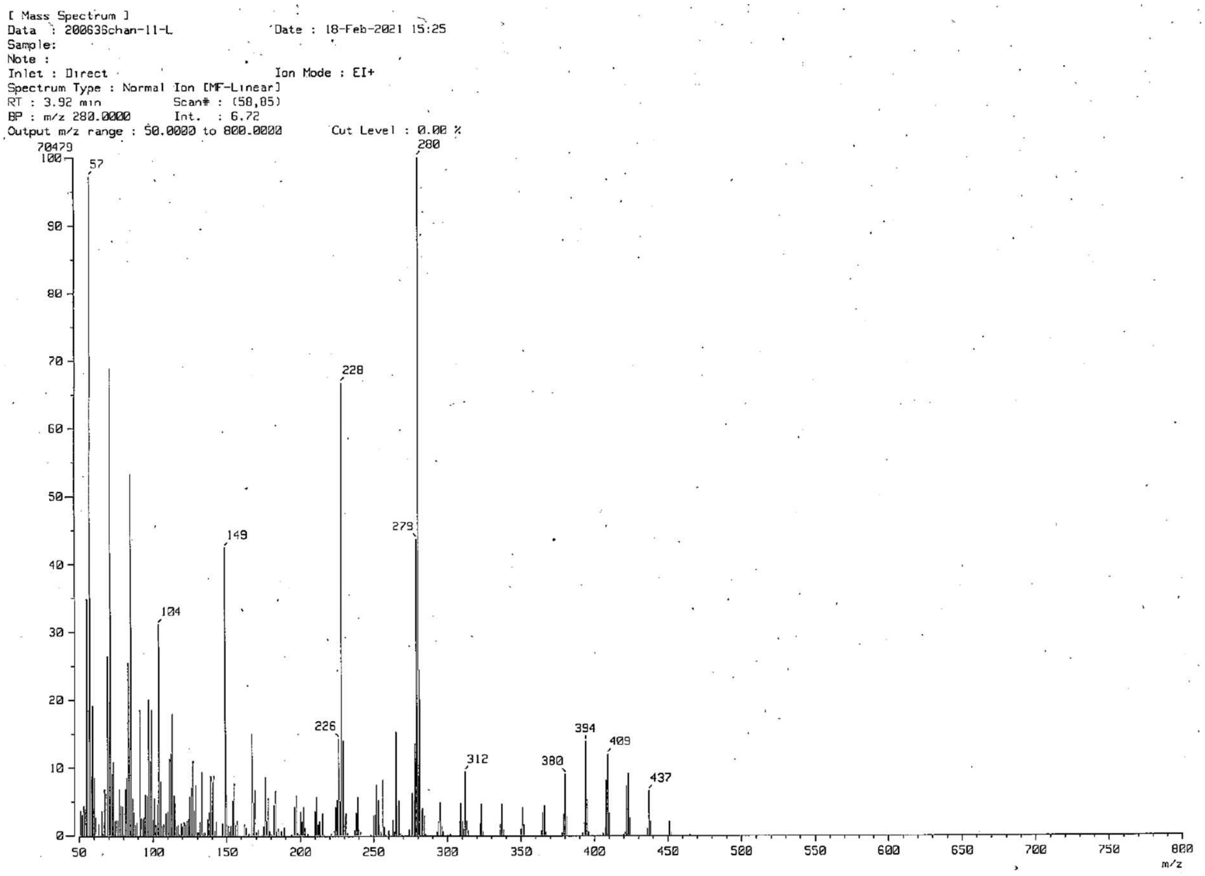

Supplementary Figure 52. MS spectrum of **compound ix**.

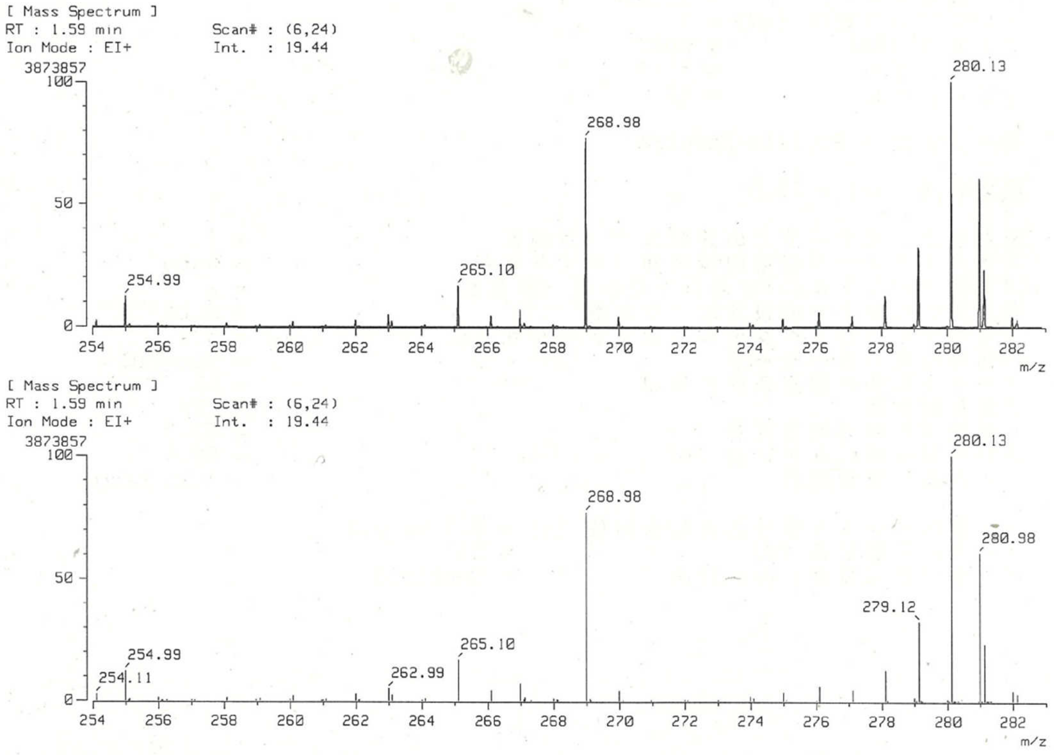

Supplementary Figure 53. HRMS spectrum of **compound ix**.

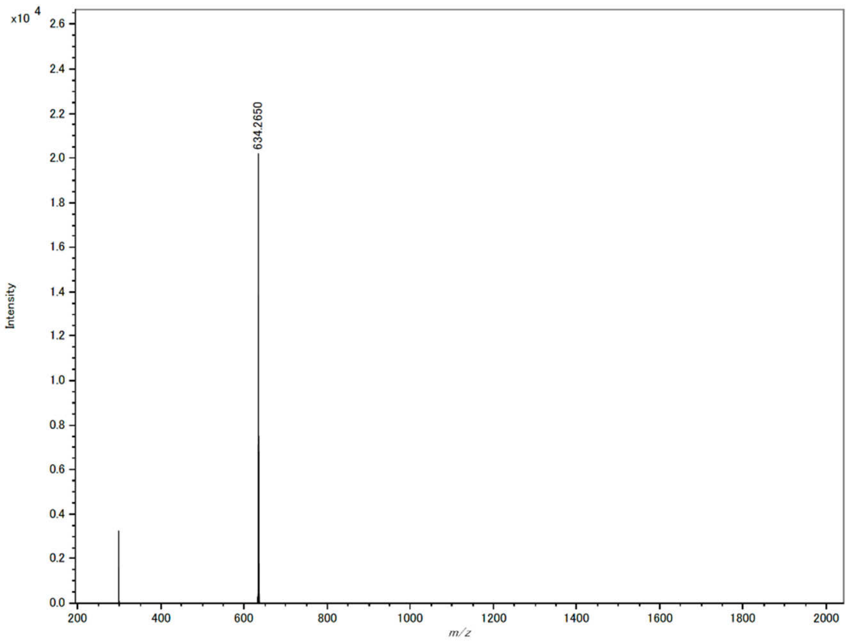

Control Parameters	
Name	SP-2000-df.spcm
Last Saved Date	2014-01-21T13:02:22
Mode	Spiral:POS
Target Plate (V)	19900
Grid (V)	-3000
Inner (V)	-4000
Outer (V)	4000
Matsuda Plate (V)	670
Einzel Lens (V)	2000
Detector (V)	-1350
Delay Time (ns)	200
Mass Range	200-2000
Sampling Interval (ns)	0.5
Digitizer Full Scale (V)	0.1
Digitizer Offset (V)	0
Real Time Smooth	OFF
Laser Intensity (%)	36
Laser Frequency (Hz)	250
Laser Shots	11750
Stage (Pa)	6.1E-05
Analyzer (Pa)	8.3E-06
Collision Cell (Pa)	8.1E-06
Mirror (Pa)	1.4E-05
Process Method	
Smoothing Points	-
Subtract Baseline	100
Instrument	
msTornado Control	1.9.2

Supplementary Figure 54. MS spectrum of **compound x**.

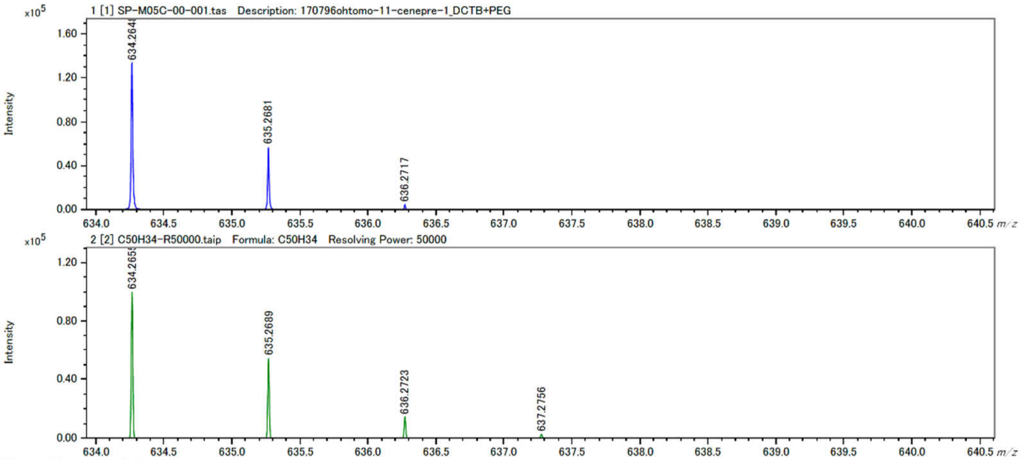

Elemental Composition Estimation

Parameters:

Mass	Tolerance	Electron Mode	Charge	DBE Range	Max Results
634.26483 ± 0.00317	5.0 ppm	Odd/Even	+1	-0.5 - 200.0	100

Elements

C 0 - 50 H 0 - 45

Results:

#	Formula	Mass	DBE	Abs. Error (u)	Error (u)	Error (ppm)
1	C50 H34	634.26550	34.0	0.00067	-0.00067	-1.05

Supplementary Figure 55. HRMS spectrum of **compound x**.

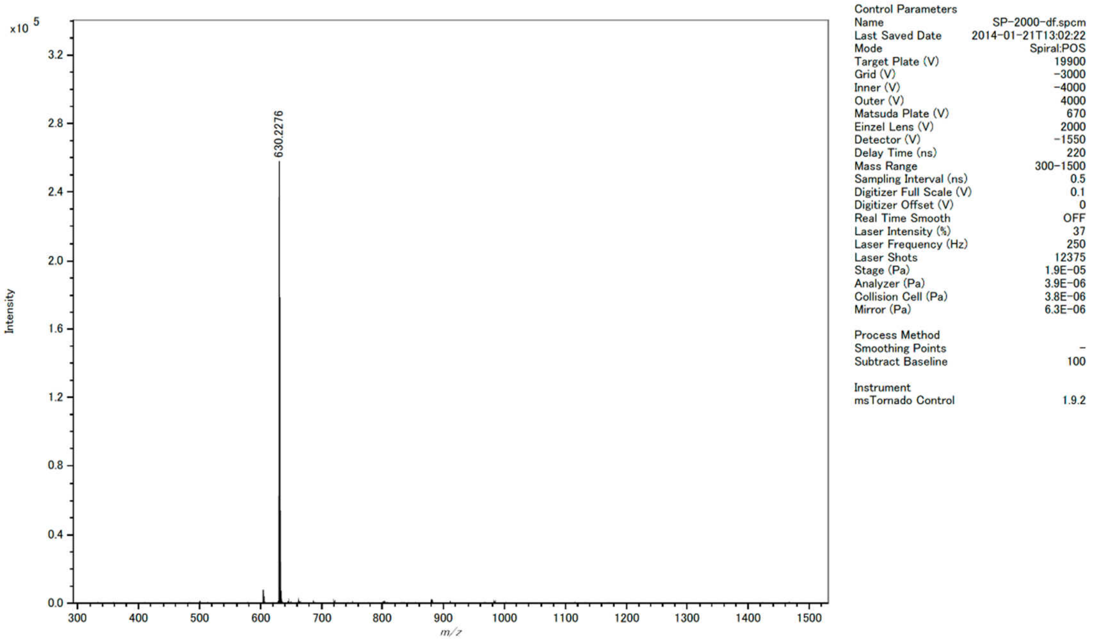

Supplementary Figure 56. MS spectrum of **compound 6**.

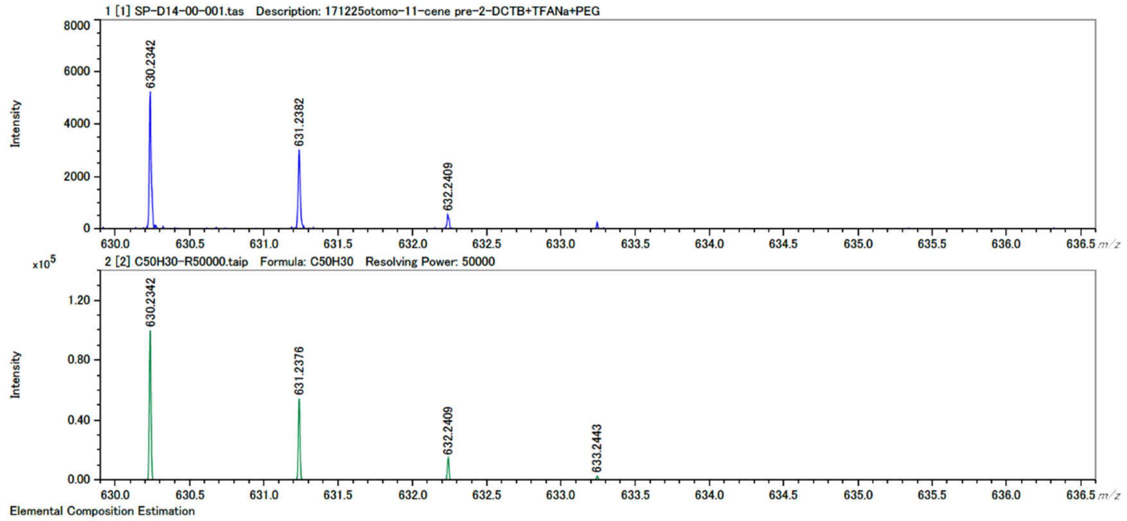

Elemental Composition Estimation

Parameters:

Mass	Tolerance	Electron Mode	Charge	DBE Range	Max Results
630.23423 ± 0.00315	5.0 ppm	Odd/Even	+1	-0.5 - 200.0	100

Elements

C 0 - 100 H 0 - 60 N 0 - 10 O 0 - 10 Na 0 - 0

Results:

#	Formula	Mass	DBE	Abs. Error (u)	Error (u)	Error (ppm)
1	C50 H30	630.23420	36.0	0.00003	0.00003	0.05
2	C36 H32 N5 O6	630.23471	23.5	0.00048	-0.00048	-0.76
3	C35 H36 N O10	630.23337	18.5	0.00086	0.00086	1.36
4	C34 H30 N8 O5	630.23337	24.0	0.00086	0.00086	1.37
5	C37 H28 N9 O2	630.23605	28.5	0.00182	-0.00182	-2.88
6	C38 H34 N2 O7	630.23605	23.0	0.00182	-0.00182	-2.89

Supplementary Figure 57. HRMS spectrum of **compound 6**.

Supplementary References

1. Hutter, J., Iannuzzi, M., Schiffmann, F. & VandeVondele, J. cp2k: atomistic simulations of condensed matter systems. *WIREs Comput. Mol. Sci.* **4**, 15–25 (2014).
2. Perdew, J. P., Burke, K. & Ernzerhof, M. Generalized Gradient Approximation Made Simple. *Phys. Rev. Lett.* **77**, 3865–3868 (1996).
3. Grimme, S., Antony, J., Ehrlich, S. & Krieg, H. A consistent and accurate ab initio parametrization of density functional dispersion correction (DFT-D) for the 94 elements H-Pu. *J. Chem. Phys.* **132**, 154104 (2010).
4. Goedecker, S., Teter, M. & Hutter, J. Separable dual-space Gaussian pseudopotentials. *Phys. Rev. B* **54**, 1703–1710 (1996).
5. VandeVondele, J. & Hutter, J. Gaussian basis sets for accurate calculations on molecular systems in gas and condensed phases. *J. Chem. Phys.* **127**, 114105 (2007).
6. Wilhelm, J., Golze, D., Talirz, L., Hutter, J. & Pignedoli, C. A. Toward GW Calculations on Thousands of Atoms. *J. Phys. Chem. Lett.* **9**, 306–312 (2018).
7. Wilhelm, J., Del Ben, M. & Hutter, J. GW in the Gaussian and Plane Waves Scheme with Application to Linear Acenes. *J. Chem. Theory Comput.* **12**, 3623–3635 (2016).
8. Neaton, J. B., Hybertsen, M. S. & Louie, S. G. Renormalization of Molecular Electronic Levels at Metal-Molecule Interfaces. *Phys. Rev. Lett.* **97**, 216405 (2006).
9. Kharche, N. & Meunier, V. Width and Crystal Orientation Dependent Band Gap Renormalization in Substrate-Supported Graphene Nanoribbons. *J. Phys. Chem. Lett.* **7**, 1526–1533 (2016).
10. Tersoff, J. & Hamann, D. R. Theory of the scanning tunneling microscope. *Phys. Rev. B* **31**, 805–813 (1985).
11. Tersoff, J. Method for the calculation of scanning tunneling microscope images and spectra. *Phys. Rev. B* **40**, 11990–11993 (1989).
12. Hapala, P. *et al.* Mechanism of high-resolution STM/AFM imaging with functionalized tips. *Phys. Rev. B* **90**, 085421 (2014).
13. Frisch, M. J. *et al. Gaussian software package*. (Gaussian, Inc., 2016).
14. Yamaguchi, K. Instability in chemical bonds: SCF, APUMP, APUCC, MR-CI AND MR-CC approaches. in *Self-Consistent Field: Theory and Applications* (eds. Carbo, R. & Klobukowski, M.) 727–823 (Elsevier, 1990).
15. Nakano, M. *et al.* (Hyper)polarizability density analysis for open-shell molecular systems based on natural orbitals and occupation numbers. *Theor. Chem. Acc.* **130**, 711–724 (2011).
16. Head-Gordon, M. Characterizing unpaired electrons from the one-particle density matrix. *Chem. Phys. Lett.* **372**, 508–511 (2003).
17. Chen, Z., Wannere, C. S., Corminboeuf, C., Puchta, R. & Schleyer, P. von R. Nucleus-Independent Chemical Shifts (NICS) as an Aromaticity Criterion. *Chem. Rev.* **105**, 3842–3888 (2005).
18. Corminboeuf, C., Heine, T., Seifert, G., Ragué Schleyer, P. von & Weber, J. Induced magnetic fields in aromatic [n]-annulenes—interpretation of NICS tensor components. *Phys. Chem. Chem. Phys.* **6**, 273–276 (2004).
19. Bohmann, J. A., Weinhold, F. & Farrar, T. C. Natural chemical shielding analysis of nuclear magnetic resonance shielding tensors from gauge-including atomic orbital calculations. *J. Chem. Phys.* **107**, 1173–1184 (1997).
20. Glendening, E. D., Landis, C. R. & Weinhold, F. NBO 6.0: Natural bond orbital analysis program. *J. Comput. Chem.* **34**, 1429–1437 (2013).
21. Geuenich, D., Hess, K., Köhler, F. & Herges, R. Anisotropy of the Induced Current Density (ACID), a General Method To Quantify and Visualize Electronic Delocalization. *Chem. Rev.* **105**, 3758–3772 (2005).
22. Keith, T. A. & Bader, R. F. W. Calculation of magnetic response properties using a continuous set of gauge transformations. *Chem. Phys. Lett.* **210**, 223–231 (1993).
23. Lu, T. & Chen, F. Multiwfn: A multifunctional wavefunction analyzer. *J. Comput. Chem.* **33**, 580–592 (2012).
24. Yakutovich, A. V. *et al.* AiiDAlab – an ecosystem for developing, executing, and sharing scientific workflows. *Comput. Mater. Sci.* **188**, 110165 (2021).
25. Tanaka, K. *et al.* A soluble bispentacenequinone precursor for creation of directly 6,6′-linked bispentacenes and a tetracyanobipentacenequinodimethane. *RSC Adv.* **3**, 15310–15315 (2013).
26. Urgel, J. I. *et al.* On-surface light-induced generation of higher acenes and elucidation of their open-shell character. *Nat. Commun.* **10**, 861 (2019).

27. Spek, A. L. PLATON, An Integrated Tool for the Analysis of the Results of a Single Crystal Structure Determination. *Acta Crystallogr. A* **46**, 34–34 (1990).
